# Inhibitory Activity of *N*- and *S*-Functionalized Monoterpene Diols Towards Monoamine Oxidases A and B

**DOI:** 10.3390/ijms26010097

**Published:** 2024-12-26

**Authors:** Alexandra V. Podturkina, Oleg V. Ardashov, Yuliya V. Soldatova, Darya A. Poletaeva, Anastasiya V. Smolina, Ekaterina P. Vasyuchenko, Yuri V. Vyatkin, Nikolai S. Li-Zhulanov, Irina I. Faingold, Nariman F. Salakhutdinov, Konstantin P. Volcho

**Affiliations:** 1 N.N. Vorozhtsov Novosibirsk Institute of Organic Chemistry, Siberian Branch of the Russian Academy of Sciences, Lavrentiev Ave. 9, 630090 Novosibirsk, Russia; podturkina@nioch.nsc.ru (A.V.P.); ardashov@nioch.nsc.ru (O.V.A.); lizhulan@nioch.nsc.ru (N.S.L.-Z.); anvar@nioch.nsc.ru (N.F.S.); 2 Federal Research Center of Problems of Chemical Physics and Medicinal Chemistry, Russian Academy of Sciences, Academician Semenov Ave. 1, 142432 Chernogolovka, Russia; soldatovayv@gmail.com (Y.V.S.); dapol@icp.ac.ru (D.A.P.); asmol@icp.ac.ru (A.V.S.); ifaingold@mail.ru (I.I.F.); 3 Institute for Artificial Intelligence, Lomonosov Moscow State University, Lomonosovsky Ave. 1 Bldg. 27, 119992 Moscow, Russia; e.vasyuchenko@iai.msu.ru (E.P.V.); y.vyatkin@iai.msu.ru (Y.V.V.)

**Keywords:** terpene, Parkinson’s disease, MAO, inhibitor, docking, artificial intelligence

## Abstract

Monoamine oxidase B (MAO-B) inhibitors are widely used as part of combination drug therapy for Parkinson’s disease. As demonstrated in both in vitro and in vivo experiments, the monoterpenoid Prottremine and some of its derivatives exhibit high antiparkinsonian activity. In this study, the inhibitory activity of Prottremine and its derivatives (including 14 new 9-*N*- and *S*-derivatives) against MAO-A and MAO-B enzymes has been investigated for the first time. Compounds containing fragments of substituted anilines have demonstrated the highest activity against MAO-A; for example, compound **28** had an IC_50_ of 178 ± 44 μM. A significant proportion of the compounds tested, including Prottremine, exhibited moderate inhibitory activity towards MAO-B, with the most active being the *o*-aminoacetophenone derivative, which had an IC_50_ of 95 ± 5 μM. A molecular docking method for studying murine MAO-A and -B enzymes was developed using AlphaFold2 (v2.3.2), with further improvements. For the MAO-B enzyme, a strong correlation was observed between the molecular docking data and the measured activity of the compounds, with the maximum binding affinity registered for the most active compound. It is conceivable that the antiparkinsonian activity of Prottremine and some of its derivatives may be partially mediated, among other mechanisms, by MAO-B enzyme inhibition.

## 1. Introduction

Parkinson’s disease (PD) is a progressive severe neurodegenerative disorder accompanied by hypokinesia, tremors, muscle rigidity, and postural instability that predominantly affects elderly patients; however, there are known cases in which individuals in their 30s and 40s develop it as well. So-called juvenile PD has also been diagnosed in adolescents under 20 years of age [1]. PD pathogenesis is still not fully understood but its key factors have been identified and include the loss of dopaminergic neurons mainly in the substantia nigra, which leads to dopamine deficiency in the brain and neurotransmitter system imbalance, as well as the accumulation of Lewy bodies, consisting mainly of improperly coiled aggregates of α-synuclein protein, both of which contribute to PD development and progression [2,3]. Molecular studies of postmortem brain samples from PD patients have found lesions associated with neuroinflammation [4]. Tissue damage, exposure to toxins, or the accumulation of altered proteins activate microglia, which begin to produce toxic cytokinins (IL-1, IL-6, TNF-α) and reactive oxygen species (superoxide and NO). In PD, α-synuclein aggregates increase the synthesis of cytokinins and ROS. The process becomes continuous and contributes to neurodegeneration [5]. In biological systems, the detoxification of such RO species as superoxide radicals (O_2_^•−^), hydrogen peroxide (H_2_O_2_), hydroxyl radicals (^•^OH), and singlet oxygen (^1^O_2_) occurs through various antioxidants, including glutathione (GSH), superoxide dismutase (SOD), and others. In PD, the capacity of the antioxidant system becomes insufficient, and free radicals cause severe damage and death of dopamine-producing cells [6].

**Figure 1:**
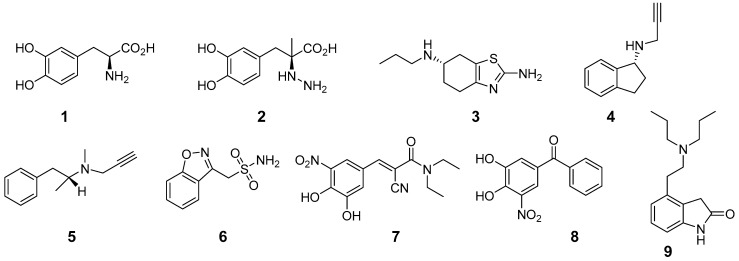
Anti-PD medications: Levodopa (**1**), Carbidopa (**2**), Pramipexole (**3**), Rasagiline (**4**), Selegiline (**5**), Zonisamide (**6**), Entacalone (**7**), Tolcalone (**8**), and Ropinirole (**9**).

Currently, there are no drugs that can prevent or at least slow the loss of dopamine neurons, and the primary goal of therapy is to manage symptoms to achieve an acceptable quality of life over the long term. The main drug for PD symptomatic therapy is Levodopa (**1**), a dopamine precursor, in combination with peripheral dopa decarboxylase inhibitors such as Carbidopa (**2**). The most common side effect of taking Levodopa during the initial stages of PD is nausea, but as the disease progresses, the effects increase to confusion, sleep disturbance, dyskinesia, and hallucinations [7]. Dopamine agonists are administered at the early stage of PD [8]. Research is also being conducted on the effects of different types of D-receptors to find new antiparkinsonian agents [9,10]. Some antiparkinsonian agents show neuroprotective effects [11]. However, about 40% of PD patients taking the agonists orally (Pramipexole (**3**), Ropinirole (**4**); see Figure 1) experience psychiatric side effects, particularly hallucinations and impulsive personality disorder. To slow down levodopa degradation, monoamine oxidase B (MAO-B) inhibitors are often included in the combination drug therapy for PD (Selegiline (**5**), Zonisamide (**6**)), as well as catechol-O-methyltransferase (COMT), which prevents the methylation of Levodopa to 3-methyldopa (Entacalone (**7**), Tolcalone (**8**)) (Figure 1) [12]. Rasagiline is a novel, highly potent irreversible monoamine oxidase (MAO-B) inhibitor [13]. The neuroprotective mechanism of its main metabolite is also being studied [14]. Zonisamide has been shown to prevent dopamine quinone formation [15] and reduce 1-methyl-4-phenyl-1,2,3,6-tetrahydropyridine (MPTP)-induced neurotoxicity in mice [16]. Selegiline is a non-competitive monoamine oxidase B inhibitor that has neuroprotective effects and has been administered to PD patients as monotherapy or in combination with Levodopa [17]. Selective MAO-B inhibitors can also be used individually during the early stages of PD [18,19,20,21].

**Scheme 1:**
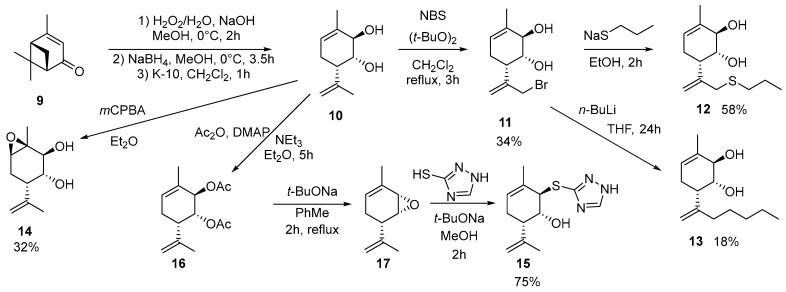
Synthesis of diol **10** (Prottremine) [22] and its 9*S*- and 9*C*-derivatives [28]; epoxydiol **14** [29], and triazole derivative **15** [36].

Earlier, using the commercially available (−)-verbenone (**9**), we prepared monoterpenoid (1*R*,2*R*,6*S*)-3-methyl-6-(prop-1-en-2-yl)cyclohex-3-en-1,2-diol (**10**) (Prottremine; see Scheme 1), which has shown antiparkinsonian activity in animal models towards various neurotoxins [22,23] and had low toxicity (LD_50_ = 4250 mg/kg) [24]. Tests for antiparkinsonian activity of all possible stereoisomers of compound **10** have shown that (1*R*,2*R*,6*S*)-stereoisomer exhibits the highest one [25]. Currently, Prottremine (**10**) is in clinical trials [26].

The presence of both hydroxyl groups and double bonds has been proven necessary for having a pronounced antiparkinsonian effect [27]. For that reason, further modification of Prottremine focused on the allylic bromination of compound **10** in which bromide **11** interacts with a set of *N-*, *S-*, and *C-*nucleophiles (Scheme 1). Among the compounds obtained in this way, compounds **12** and **13** with respective *n*-PrS- and *n*-Bu- substituents were found to be the most active [28].

The oxidation of Prottremine (**10**) using *m*CPBA enabled the synthesis of its active epoxy metabolite **14**, which was found to promote the survival of cultured dopamine neurons, protect dopamine neurons from toxin-induced degeneration, and trigger the mitogen-activated protein kinase (MAPK) signaling cascade in neuronal cells (Scheme 1). In a 1-methyl-4-phenyl-1,2,3,6-tetrahydropyridine (MPTP) neurotoxicity murine model, the compound increased the density of dopamine neuronal fibers in the striatum, highlighting its ability to stimulate striatal reinnervation and thus halt PD progression [29]. Epoxydiol **14** also protected dopamine neurons from degeneration in an in vitro and in vivo PD model based on Rotenone, a mitochondrial toxin [30].

Using another approach to the modification of **10**, we synthesized a sulfur derivative, compound **15**, containing a heterocyclic fragment **15**, which has been shown to improve potency in an in vitro survival assay for dopamine (DA) neurons compared to a sibling compound at an effective concentration of 1–10 nM. It retained its biological activity in both MPTP- [31,32,33] and haloperidol-induced PD models in animals [34,35], and readily penetrated the blood–brain barrier (BBB) [36]. The synthesis began with the preparation of acetate **16,** according to the procedure [37], which was further converted into epoxide **17**. The reaction of epoxide **17** with 1*H*-1,2,4-triazole-3-thiol, without additional purification, yielded the target compound **15** (Scheme 1).

Despite the large amount of research conducted and repeated confirmation of the high antiparkinsonian activity of this structural type of compounds, the molecular targets of their activity remain unknown. In this present study, we investigated for the first time whether Prottremine and its derivatives, both known and first-time synthesized, affect the catalytic activity of MAO-A and -B enzymes.

## 2. Results and Discussion

### 2.1. Chemistry

Using the previously developed techniques, initial diol **10** [22], its active metabolite **14** [29], and derivative **15** [36] were synthesized. In addition to these compounds, a set of new 9-*S*- and *N*-derivatives containing aliphatic, aromatic, and heteroaromatic substituents was obtained following the classical scheme of allylic bromination and nucleophilic substitution. There are various methods of allylic bromination, both radical and electrophilic, particularly in the case of a monoterpenoid with a *p*-menthane carvone backbone [38,39], as well as in synthetically equivalent allylic chlorination [40]. At the same time, the allylic chlorination reaction of limonene proceeds non-selectively [41]. In this case, anionic methods based on Li-based organic compounds are used [42].

**Scheme 2:**
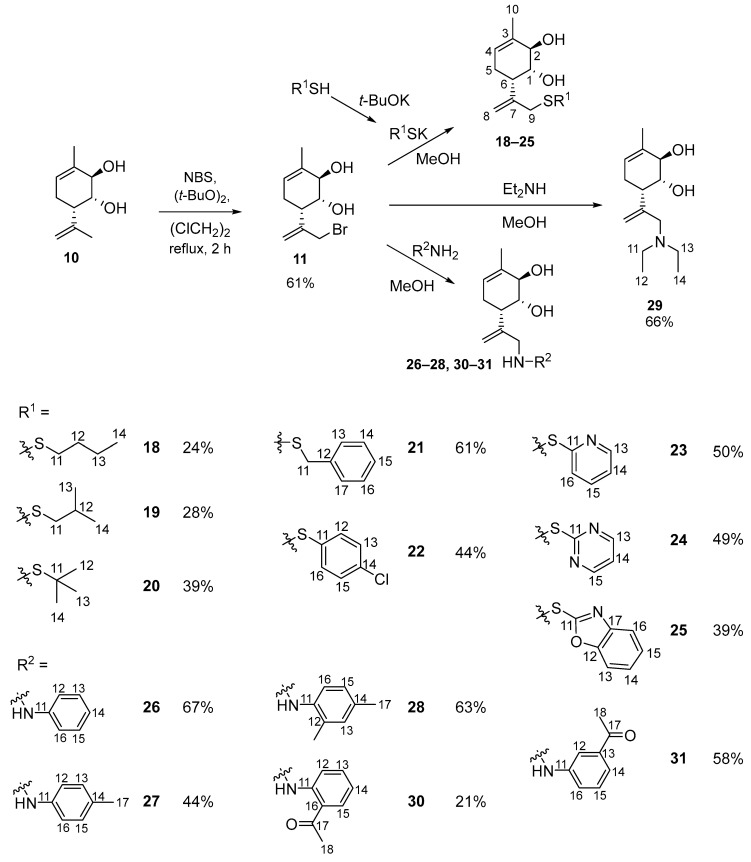
Synthesis of target compounds **18**–**31**.

Bromide **11** was synthesized following an improved procedure by boiling diol **10** and NBS-(*t*-BuO)_2_ in 1,2-dichloroethane, the product yield being 61%. Halogen substitution in allyl-containing compounds is a classical method of functionalization [43]. The bromide interacted with nucleophiles in MeOH; for amino derivatives, directly with amines; and for *S*-derivatives, through the potassium salts of corresponding mercaptans obtained by interaction with *t*-BuOK. The yields for **18**–**31** varied from 24% to 67% (Scheme 2).

### 2.2. Biology

In this study, we investigated the in vitro effects of Prottremine and its derivatives on MAO-A and -B enzymes in the crude mitochondrial fractions obtained from a murine brain. The inhibition data obtained (Table 1) demonstrated that most of the compounds, including Prottremine, were not potent inhibitors of MAO-A. Small but statistically significant activity was detected for compounds **22**, **24**, and **31** at a dose of 100 μM. The highest inhibitory effect was observed for **28** and **30**, which contained fragments of substituted anilines at position 9. Compound **28** had an IC_50_ value of 178 ± 44 μM for MAO-A inhibition (clorgiline, used as a positive control, had an IC_50_ of 1.36 nM). Conversely, compound **29**, which contains a diethylamine residue at position 9, slightly increased MAO-A activity at the dose of 100 μM. It is possible that compounds **28**, **30**, and **31** exhibit anxiolytic and antidepressant effects, but additional in vivo studies are needed to confirm this.

The tested compounds showed a better potency to inhibit the MAO-B enzyme, a well-known therapeutic target for the treatment of neurodegenerative disorders such as PD. In particular, both Prottremine and its active metabolite epoxide **14** showed weak activity at a dose of 100 µM. However, Prottremine retained its inhibitory activity even when the dose was reduced to 10 µM, so its antiparkinsonian effect may be attributed, to a certain degree, to MAO-B inhibition. The substituted derivatives **18**, **19**, and **21**–**25**, as well as the aniline derivatives **27**, **28**, and **31**, also showed slight activity. Compound **30** showed the greatest inhibitory activity towards MAO-B enzymes, reaching an IC_50_ value of 95 ± 5 µM (deprenyl as a positive control has IC_50_ of 0.1 nM).

### 2.3. Structure Analysis and Molecular Docking

#### 2.3.1. Three-Dimensional Enzyme Structures and Their Preparation for Docking

The predicted protein structures were obtained using the AlphaFold2 network and downloaded from the UniProt database [44] (ID: Q64133 and Q8BW75 for MAO-A and -B, respectively). Using UCSF Chimera software 1.16 [45], hydrogen was added to the structures, the partial charges were calculated according to the Amberff99SB force field [46], and the proteins were minimized using the steepest descent method for 1000 steps. For protein sequences, homologous structures were sought for using BLAST (Basic Local Alignment Search Tool, https://www.uniprot.org/blast/, access date: July 2024) to detect the closest proteins with the crystal structures of protein-ligand complexes.

AlphaFold2 creates protein structures without cofactors but reproduces the binding pocket for them well. The enzymes MAO-A and -B have a coenzyme FAD (flavin adenine dinucleotide) molecule in their structure, which is necessary for the enzyme function. The position of this cofactor is important for determining the binding pocket of inhibitor molecules to exclude the overlap of the inhibitor molecule and FAD. To determine the position of the coenzyme and the binding pocket for small molecules, the structures of 2Z5X (MAO-A Homo sapiens) and 7P4H (MAO-B Homo sapiens) [47,48] were selected from the PDB database; their RMSD for Cα-atoms was 0.419 and 0.262 Å for MAO-A and -B, respectively. To determine the active center of the murine enzyme, the enzymes from *Homo sapiens* and *Mus musculus* were structurally aligned with each other, followed by identification of the amino acids comprising the active center of ligand binding in PyMol-2.5.4 [49].

Preliminary calculations for molecular docking showed that the *Mus musculus* enzyme structures generated by AlphaFold2 had an insufficient pocket for binding small molecules and a FAD cofactor. To solve this problem, the following approach was used. Due to the high structural similarity, the FAD molecule and the ligand from the human structures (2Z5X and 7P4H) were placed in the structure of the murine enzymes by structural alignment of their protein parts. Then, the obtained complexes were reduced to the energy minimum by the steepest descent method in the UCSF Chimera program. This procedure allowed us to obtain the necessary pocket for binding the inhibitors.

#### 2.3.2. Preparation of Small-Molecule Structures

The small molecules were designed in DataWarrior software V06.01.04 [50], and their 3D structures were designed in the OpenBabel 3.1.1 package [51].

#### 2.3.3. Molecular Docking

PyRx’s integrated AutoDock Vina softwarewas employed for the molecular docking phase (PyRx-v0.8) [52]. Protein and ligand molecules were converted to a PDBQT format. The Autodock Vina algorithm does not take cofactor molecules into account during the docking process. The ligand-binding search space defined the found binding pocket so that it did not overlap with the enzyme’s FAD–cofactor binding site, which was located inside the protein cavity, and also did not allow inhibitor molecules to bind to the protein surface. The grid size was 18.7 Å × 17.2 Å × 16.3 Å for the MAO-A enzyme and 18.7 Å × 17.1 Å × 19.4 Å for the MAO-B enzyme with an exhaustiveness of 8. For each ligand, the best position was taken based on the calculated binding energy of the molecule to the enzyme.

#### 2.3.4. Analysis of Enzyme Active Centers

**Figure 2:**
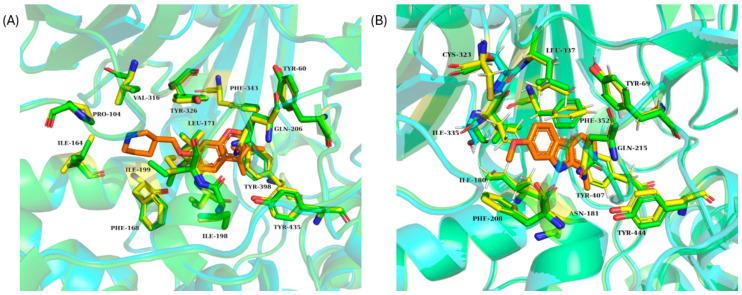
Comparison of MAO-A (**A**) and MAO-B (**B**) enzyme ligand-binding centers. The structures of the murine enzyme are colored turquoise; the amino acids being a part of the active center are colored yellow and their labels indicate residue name and number. The structures of human enzyme are colored green; the amino acids included in the active center are shown as sticks, and the rest as cartoons. The inhibitors from the complexes are colored orange and shown as sticks.

To identify the amino acids that form the active site, residues that are within 4 Å of the bound ligand were identified to find the corresponding residues in the murine enzyme structures (Figure 2). The results showed that there are 13 such residues for the human MAO-A protein, all of which are identical to those in the murine enzyme (100% identity). For the human MAO-B protein, 14 residues were identified, with the leucine residue at position 164 being replaced by isoleucine in the murine enzyme, and the isoleucine at position 316 being replaced by valine in the murine enzyme (85.71% identity).

An analysis of the active centers of MAO-A and -B enzymes from *Homo sapiens* and *Mus musculus* demonstrated that the two had a high percentage of homology and structural similarity, which suggested that the small molecule binding site in the *Mus musculus* enzymes corresponds to that in the *Homo sapiens* enzymes, and so, for molecular docking, the binding pocket described in Table 2 was selected.

#### 2.3.5. Molecular Docking Results

For each substance, eight conformations were generated in the active center of the protein for which the binding affinity had been calculated. For each molecule, a conformation with the minimum affinity value was selected (Table 3).

**Figure 3:**
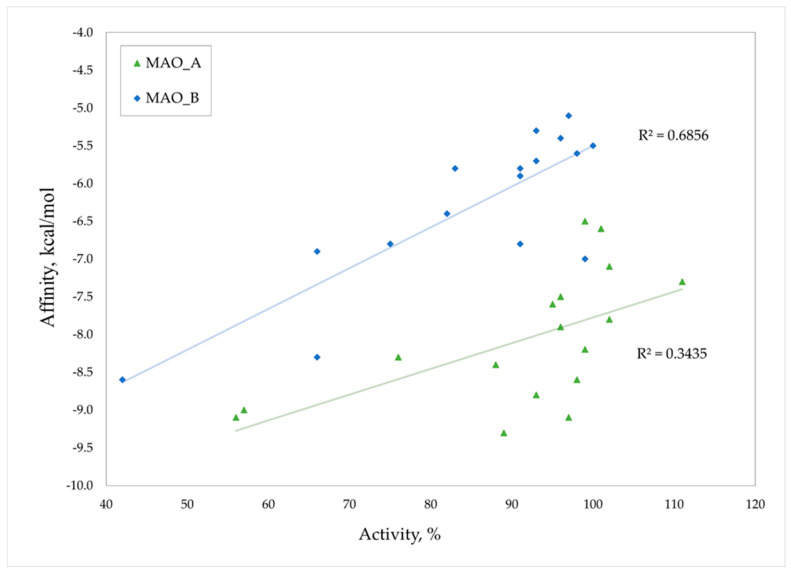
Correlation between the minimum ligand-to-protein affinity value and experimental enzyme activity in the presence of this ligand (the data do not include those for DMSO).

The predicted affinity was compared with the experimental data on changes in enzyme activity (Figure 3).

**Figure 4:**
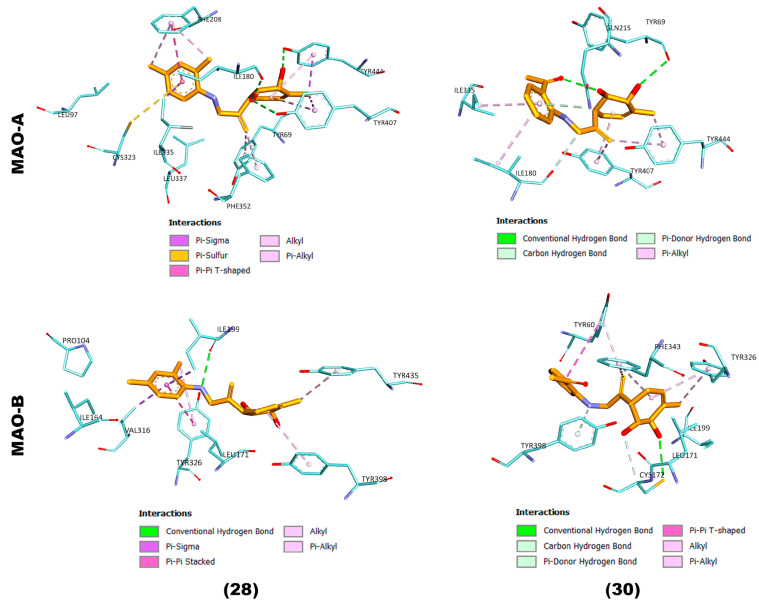
The site binding compounds **28** and **30** to MAO-A (**the top panel**) and to MAO-B (**the bottom panel**) (Created with BIOVIA Discovery Studio Visualizer v24.1.0.23298).

According to the docking results, the minimum affinity value of binding to MAO-B enzyme was observed for compound **30** (−8.60), having the highest activity in in vitro experiments. It is noteworthy that for the MAO-B enzyme, the synthesized set of compounds had a good correlation between the molecular docking data and the measured activity towards the enzyme (Pearson correlation coefficient = 0.586, *p*-value = 0.013, R^2^ = 0.685, Figure 3). As for the MAO-A enzyme, the correlation was not that high (Pearson correlation coefficient = 0.828, *p*-value = 4 × 10^−5^, R^2^ = 0.343, Figure 3); nevertheless, compound **28**, being the most active compound in in vitro experiments, had the second lowest affinity value (−9.10) based on the docking results, only slightly behind compound **27** (−9.30). The sites binding compound **30** to MAO-B and compound **28** to MAO-A are depicted in Figure 4. Overall, given the moderate activity of compound **30** towards MAO-B and the good correlation with mathematical significance by *p*-value of activity and affinity values, it can be assumed that a number of Prottremine derivatives may exhibit antiparkinsonian activity mediated by MAO-B enzyme inhibition among other action pathways.

Molecular docking is a widely used method in the study of interactions between proteins and small molecules. However, it has some limitations. First, the accuracy of its results depends on the quality of the protein structure used. In this work, the structure predicted by AlphaFold2, with the ligand binding site obtained through molecular modeling methods, was used as a spatial model of the MAO enzymes. Unlike experimentally determined protein–ligand complexes, this approach leaves uncertainty in the reliability of the obtained results and the position of the protein side chains inside the pocket. Although AlphaFold2 shows impressive results in predicting protein tertiary structure, the prediction of binding pockets remains a bottleneck. It is possible that, while using the structure of the MAO farmer complex with the ligand obtained by experimental methods, our results of the correlation between the experimental values of enzyme activity and the calculated binding energy could be improved; however, even with this approach, the correlation is significant. The laboratory mouse (*Mus musculus*) is a widely used model organism, so we would like to emphasize the importance of further work to obtain the experimentally determined MAO enzyme complexes.

## 3. Materials and Methods

### 3.1. Chemistry

All commercially available compounds and solvents were used without extra purification. General information: ^1^H- and ^13^C-NMR were recorded on a Bruker Avance III 600 spectrometer (Bruker Corporation, Karlsruhe, Germany) apparatus at 600.30 MHz (^1^H) and 150.95 MHz (^13^C) for ^1^H and ^13^C, respectively, in CDCl_3_. Chemical shifts are expressed in δ (ppm) related to residual CHCl_3_ (δ(H) 7.24, δ(C) 76.90 ppm); J are represented in Hz. Structure determinations were carried out by analyzing the ^1^H-NMR spectra, including ^1^H-^1^H double resonance spectra, ^1^H-^1^H 2D homonuclear correlation (COSY), NOESY spectra, J-modulated ^13^C-NMR spectra (JMOD), and ^13^C-^1^H 2D heteronuclear correlation with one bond and long-range spin–spin coupling constants (C-H COSY, HSQC, HMBC, HXCO). HR-MS: DFS Thermo Scientific spectrometer in a full scan mode (15–500 *m/z*, 70 eV electron-impact ionization, direct sample introduction) (Thermo Fisher Scientific, Waltham, MA, USA). Purity of compounds and products analysis was checked by GC/MS: Agilent 7890A (Agilent Technologies, Santa Clara, CA, USA) with a quadrupole mass spectrometer Agilent 5975C as a detector, HP-5MS quartz column, 30,000 × 0.25 mm, and He (1 atm) as carrier gas. Optical rotation was measured with polAAr 3005 spectrometer (Optical Activity Ltd., Huntingdon, UK, CHCl_3_ solvent). All the target compounds reported in this paper have a purity of at least 95%. ((1*R*,2*R*,6*S*)-3-Methyl-6-(prop-1-en-2-yl)cyclohex-3-ene-1,2-diol (**1**) (${\left[\alpha \right]}_{D}^{31}$ −49.1 (*c* 2.6, CHCl_3_)) was prepared from (−)-verbenone (Sigma-Aldrich, St. Louis, MO, USA) (${\left[\alpha \right]}_{D}^{25}$ −210.5 (*c* 0.77, CHCl_3_)) according to previously described procedure [53]. (1*S*,2*S*,3*R*,4*S*,6*R*)-1-Methyl-4-(prop-1-en-2-yl)-7-oxabicyclo[4.1.0]heptane-2,3-diol (**14**) was synthesized according to a previously described procedure [29]. (1*R*,2*R*,6*S*)-2-(1*H*-1,2,4-Triazol-3-ylthio)-3-methyl-6-(prop-1-en-2-yl)cyclohex-3-enol (**15**) was synthesized according to a previously described procedure [36]. Please be aware that the numbering of atoms in the compounds is intended solely for the purpose of assigning signals in the NMR spectra and it does not correspond with the nomenclature used for naming the compounds (Copies of ^1^H and ^13^C NMR Spectra can be found in the Supplementary Materials).

**(1*R*,2*R*,6*S*)-6-(3-Bromoprop-1-en-2-yl)-3-methylcyclohex-3-ene-1,2-diol** (**11**). Freshly recrystallized *N*-bromosuccinimide (2.27 g, 12.8 mmol) was added to a solution of (1*R*,2*R*,6*S*)-3-methyl-6-(prop-1-en-2-yl)cyclohex-3-ene-1,2-diol (**10**) (2.05 g, 12.2 mmol) and (*t*-BuO)_2_ (1.97 g, 13.5 mmol) in (CH_2_Cl)_2_ (60 mL). The reaction mixture was boiled for 1.5 h. The solvent was evaporated and the residue was chromatographed using hexane solutions of ether and chloroform as eluent (0–100% gradient) to yield the product, compound **11** (1.84 g, 7.45 mmol, 61%). The spectral data of compound **11** agreed with the literature data [28].

**Method A. General procedure for *S*-derivatives 18–25**. Potassium *t*-butoxide (*t*-BuOK, 0.54 mmol) was added to a solution of corresponding thiol (RSH, 0.50 mmol) in MeOH (5 mL), and the reaction mixture was stirred for 10 min. Then, a solution of (1*R*,2*R*,6*S*)-6-(3-bromoprop-1-en-2-yl)-3-methylcyclohex-3-ene-1,2-diol (**11**, 0.44 mmol) in MeOH (5 mL) was added and the reaction mixture was stirred for 24 h. The solvent was evaporated, and water (1 mL) and brine (9 mL) were added to the residue. The product was extracted with ethylacetate (3 × 10 mL). The combined extracts were dried over Na_2_SO_4_. The desiccant was filtered off. The solvent was evaporated. The residue was purified by CC on silica gel (4.5 g) using 0–100% ethylacetate gradient in hexane as eluent.

**Method B. General procedure for *N*-derivatives 26–31**. Corresponding amine (RNH_2_ or R_2_NH, 7.0 mmol) was added to a solution of (1*R*,2*R*,6*S*)-6-(3-bromoprop-1-en-2-yl)-3-methylcyclohex-3-ene-1,2-diol (**11**, 0.70 mmol) in MeOH (10 mL). The reaction mixture was stirred for 24 h. The solvent was evaporated, and 1 M NaOH solution (10 mL) was added to the residue. The product was extracted with CHCl_3_ (3 × 10 mL). The combined extracts were dried over Na_2_SO_4_. The desiccant was filtered off. The solvent was evaporated. The residue was purified by CC on silica gel (4.5 g) using 0–100% ethylacetate gradient in hexane as eluent.

**(1*R*,2*R*,6*S*)-6-(3-(Butylthio)prop-1-en-2-yl)-3-methylcyclohex-3-ene-1,2-diol** (**18**). Method A. Yield: 24%. ${\left[\alpha \right]}_{D}^{26.3}$ = −4.8 (*c* 0.167, CHCl_3_). ^1^H-NMR (CDCl_3_, δ_H_): 0.87–0.91 (m, 3H, H-14), 1.32–1.42 (m, 2H, H-13), 1.49–1.58 (m, 2H, H-12), 1.80–1.82 (m, 3H, H-10), 2.00 (dm, 1H, ^2^J 17.8 Hz, H-5e), 2.21–2.28 (m, 1H, H-5a), 2.37–2.46 (m, 2H, H-11), 2.74 (br.dd, 1H, J_6a,5a_ 11.0, J_6a,5e_ 5.6 Hz, H-6a), 3.19 (d., 1H, ^2^J 13.5 Hz, H-9), 3.22 (d, 1H, ^2^J 13.5 Hz, H-9′), 3.89 (br.d., 1H, J_2,1_ 3.2 Hz, H-2), 3.91 (dd, 1H, J_1,2_ 3.2, J_1,6a_ 2.0 Hz, H-1), 5.04 (s, 2H, 2H-8), 5.62–5.65 (m, 1H, H-4). ^13^C-NMR, δ_C_: 71.63 (d; C-1), 72.05 (d; C-2), 131.83 (s; C-3), 125.04 (d; C-4), 25.29 (t; C-5), 37.24 (d; C-6), 145.19 (s; C-7), 114.32 (t; C-8), 37.26 (t; C-9), 20.66 (q; C-10), 31.10 (t; C-11), 30.68 (t; C-12), 21.90 (t; C-13), 13.56 (q; C-14). HR-MS: 256.1490 (*M*^+^, C_14_H_24_O_2_S; calc. 256.1492).

**(1*R*,2*R*,6*S*)-6-(3-(Isobutylthio)prop-1-en-2-yl)-3-methylcyclohex-3-ene-1,2-diol** (**19**). Method A. Yield: 28%. ${\left[\alpha \right]}_{D}^{26.3}$ = −11.6 (*c* 0.447, CHCl_3_). ^1^H-NMR (CDCl_3_, δ_H_): 0.956 and 0.946 (2d, 6H, J_13,12_ = J_14,12_ = 6.6 Hz, 3H-13 and 3H-14), 1.71–1.81 (m, 1H, H-12), 1.80–1.82 (m, 3H, 3H-10), 1.93 (br.s., 1H, OH), 2.00 (dm, 1H, ^2^J 17.8 Hz, H-5e), 2.20–2.28 (m, 1H, H-5a), 2.27 (dd, 1H, ^2^J 12.6, J_11,12_ 7.1 Hz, H-11), 2.33 (dd, 1H, ^2^J 12.6, J_11,12_ 7.1 Hz, H-11′), 2.74 (br.dd, 1H, J_6a,5a_ 11.0, J_6a,5e_ 5.3 Hz, H-6a), 3.18 (br.s., 2H, 2H-9), 3.89 (br.d., 1H, J_2,1_ 3.0 Hz, H-2), 3.91 (dd, 1H, J_1,2_ 3.0, J_1,6a_ 1.9 Hz, H-1), 5.03 (br.s., 1H, H-8), 5.03–5.05 (m, 1H, H-8′), 5.62–5.66 (m, 1H, H-4). ^13^C-NMR, δ_C_: 71.63 (d; C-1), 72.04 (d; C-2), 131.81 (s; C-3), 124.84 (d; C-4), 25.27 (t; C-5), 37.23 (d; C-6), 145.21 (s; C-7), 114.40 (t; C-8), 37.73 (t; C-9), 20.66 (q; C-10), 40.15 (t; C-11), 28.05 (d; C-12), 21.89 and 22.05 (2q; C-13 and C-14). HR-MS: 256.1491 (*M*^+^, C_14_H_24_O_2_S; calc. 256.1492).

**(1*R*,2*R*,6*S*)-6-(3-(*tert*-Butylthio)prop-1-en-2-yl)-3-methylcyclohex-3-ene-1,2-diol** (**20**). Method A. Yield: 39%. ${\left[\alpha \right]}_{D}^{23}$= −20.4 (*c* 0.52, CHCl_3_). ^1^H-NMR (CDCl_3_, δ_H_): 1.31 (s, 9H, 3H-12, 3H-13 and 3H-14), 1.78–1.81 (m, 3H, 3H-10), 1.99 (dm, 1H, ^2^J 17.8 Hz, H-5e), 2.13 (br.s., 1H, OH), 2.20–2.28 (m, 1H, H-5a), 2.71 (br.dd, 1H, J_6a,5a_ 10.9, J_6a,5e_ 5.3 Hz, H-6a), 3.24 (br.d., 1H, ^2^J 12.3 Hz, H-9), 3.31 (d, 1H, ^2^J 12.3 Hz, H-9′), 3.88 (br.d., 1H, J_2,1_ 3.0 Hz, H-2), 3.91 (dd, 1H, J_1,2_ 3.0, J_1,6a_ 2.0 Hz, H-1), 5.01–5.03 (m, 1H, H-8), 5.20 (br.s., 1H, H-8′), 5.61–5.64 (m, 1H, H-4). ^13^C-NMR, δ_C_: 71.62 (d; C-1), 71.99 (d; C-2), 131.75 (s; C-3), 125.05 (d; C-4), 25.20 (t; C-5), 38.08 (d; C-6), 145.98 (s; C-7), 114.79 (t; C-8), 33.81 (t; C-9), 20.65 (q; C-10), 42.83 (s; C-11), 20.65 (q; 3C, C-12, C-13 and C-14). HR-MS: 238.1382 (*M*^+^-H_2_O, C_14_H_22_OS; calc. 238.1386).

**(1*R*,2*R*,6*S*)-6-(3-(Benzylthio)prop-1-en-2-yl)-3-methylcyclohex-3-ene-1,2-diol** (**21**). Method A. Yield: 39%. ${\left[\alpha \right]}_{D}^{26.3}$ = +5.5 (*c* 0.110, CHCl_3_). ^1^H-NMR (CDCl_3_, δ_H_): 1.79–1.82 (m, 3H, 3H-10), 1.99 (dm, 1H, ^2^J 17.8 Hz, H-5e), 2.23 (ddm, 1H, ^2^J 17.8, J_5a,6a_ 11.0 Hz, H-5a), 2.71 (br.dd, 1H, J_6a,5a_ 11.0, J_6a,5e_ 5.3 Hz, H-6a), 3.10–3.16 (m, 2H, 2H-9), 3.62 (d, 1H, ^2^J 13.5 Hz, H-11), 3.64 (d, 1H, ^2^J 13.5 Hz, H-11′), 3.86 (dd, 1H, J_1,2_ 3.0, J_1,6a_ 1.9 Hz, H-1), 3.87 (br.d., 1H, J_2,1_ 3.0 Hz, H-2), 5.06 (br.s., 1H, H-8), 5.07–5.09 (m, 1H, H-8′), 5.61–5.65 (m, 1H, H-4), 7.20–7.25 (m, 1H, H-15), 7.27–7.32 (m, 4H, H-13, H-14, H-16, H-17). ^13^C-NMR, δ_C_: 71.49 (d; C-1), 72.02 (d; C-2), 131.77 (s; C-3), 125.04 (d; C-4), 25.25 (t; C-5), 37.19 (d; C-6), 144.83 (s; C-7), 114.69 (t; C-8), 36.81 (t; C-9), 20.65 (q; C-10), 35.37 (t; C-11), 137.87 (s; C-12), 128.37 and 128.92 (2d; 4C, C-13, C-14, C-16 and C-17), 126.90 (d; C-15). HR-MS: 272.1232 (*M*^+^-H_2_O, C_17_H_20_OS; calc. 272.1229).

**(1*R*,2*R*,6*S*)-6-(3-(4-Chlorophenylthio)prop-1-en-2-yl)-3-methylcyclohex-3-ene-1,2-diol** (**22**). Method A. Yield: 44%. ${\left[\alpha \right]}_{D}^{26.3}$ = −6.0 (*c* 0.43, CHCl_3_). ^1^H-NMR (CDCl_3_, δ_H_): 1.79–1.82 (m, 3H, 3H-10), 2.00 (dm, 1H, ^2^J 17.8 Hz, H-5e), 2.17–2.26 (m, 1H, H-5a), 2.76 (br.dd, 1H, J_6a,5a_ 10.6, J_6a,5e_ 5.3 Hz, H-6a), 3.57 (d, 1H, ^2^J 13.6 Hz, H-9), 3.60 (d, 1H, ^2^J 12.3 Hz, H-9′), 3.89 (br.d., 1H, J_2,1_ 3.0 Hz, H-2), 3.92 (dd, 1H, J_1,2_ 3.0, J_1,6a_ 2.0 Hz, H-1), 4.99 (br.s., 1H, H-8), 5.00 (br.s., 1H, H-8′), 5.61–5.65 (m, 1H, H-4), 7.22 (dm, 2H, J_13,12_ = J_15,16_ 8.7 Hz, H-13 and H-15), 7.24 (dm, 2H, J_12,13_ = J_16,15_ 8.7 Hz, H-12 and H-16). ^13^C-NMR, δ_C_: 71.58 (d; C-1), 72.08 (d; C-2), 131.80 (s; C-3), 125.00 (d; C-4), 25.36 (t; C-5), 37.31 (d; C-6), 144.22 (s; C-7), 115.12 (t; C-8), 40.78 (t; C-9), 20.58 (q; C-10), 134.09 (s; C-11), 131.93 (d; 2C, C-12 and C-16), 128.87 (d; 2C, C-13 and C-15), 132.63 (s; C-14). HR-MS: 292.0678 (*M*^+^-H_2_O, C_16_H_17_OClS; calc. 292.0683).

**(1*R*,2*R*,6*S*)-3-Methyl-6-(3-(pyridin-2-ylthio)prop-1-en-2-yl)cyclohex-3-ene-1,2-diol** (**23**). Method A. Yield: 50%. ${\left[\alpha \right]}_{D}^{26.3}$ = −9.5 (*c* 0.147, CHCl_3_). ^1^H-NMR (CDCl_3_, δ_H_): 1.77–1.80 (m, 3H, 3H-10), 2.01 (dm, 1H, ^2^J 17.8 Hz, H-5e), 2.27–2.35 (m, 1H, H-5a), 2.71–2.76 (m, 1H, H-6a), 3.85 (d, 1H, ^2^J 15.4 Hz, H-9), 3.92 (br.d., 1H, J_2,1_ 4.1 Hz, H-2), 3.95 (d, 1H, ^2^J 15.4 Hz, H-9′), 4.00 (dd, 1H, J_1,2_ 4.1, J_1,6a_ 2.7 Hz, H-1), 5.02 (br.s., 1H, H-8), 5.34–5.36 (m, 1H, H-8′), 5.56–5.60 (m, 1H, H-4), 6.99 (ddd, 1H, J_14,15_ 7.4, J_14,13_ 5.1, J_14,18_ 0.9 Hz, H-14), 7.20 (dm, 2H, J_16,15_ 8.1 Hz, H-16), 7.48 (ddd, 1H, J_15,16_ 8.1, J_15,14_ 7.4, J_15,13_ 1.9 Hz, H-14), 8.36 (dm, 2H, J_13,14_ 5.1 Hz, H-13). ^13^C-NMR, δ_C_: 72.95 (d; C-1), 72.22 (d; C-2), 132.37 (s; C-3), 124.17 (d; C-4), 26.15 (t; C-5), 39.30 (d; C-6), 145.55 (s; C-7), 113.59 (t; C-8), 34.81 (t; C-9), 20.24 (q; C-10), 158.22 (s; C-11), 148.85 (d; C-13), 119.64 (d; C-14), 136.29 (d; C-15), 122.68 (d; C-16). HR-MS: 277.1129 (*M*^+^, C_15_H_19_O_2_NS; calc. 277.1131).

**(1*R*,2*R*,6*S*)-3-Methyl-6-(3-(pyrimidin-2-ylthio)prop-1-en-2-yl)cyclohex-3-ene-1,2-diol** (**24**). Method A. Yield: 50%. ${\left[\alpha \right]}_{D}^{26.3}$ = −21.1 (*c* 0.493, CHCl_3_). ^1^H-NMR (CDCl_3_, δ_H_): 1.75–1.79 (m, 3H, 3H-10), 2.06 (dm, 1H, ^2^J 17.8 Hz, H-5e), 2.29 (ddm, 1H, ^2^J 17.8, J_5a,6a_ 9.8 Hz, H-5a), 2.65–2.71 (m, 1H, H-6a), 3.84–3.91 (m, 2H, 2H-9), 3.89 (br.d., 1H, J_2,1_ 3.5 Hz, H-2), 4.01 (dd, 1H, J_1,2_ 3.5, J_1,6a_ 2.4 Hz, H-1), 5.05 (br.s., 1H, H-8), 5.41 (br.s., 1H, H-8′), 5.57–5.60 (m, 1H, H-4), 6.95 (t, 1H, J_14,13_ = J_14,15_ 4.9 Hz, H-14), 8.46 (d, 2H, J_13,14_ = J_15,14_ = 5.1 Hz, H-13 and H-15). ^13^C-NMR, δ_C_: 72.38 (d; C-1), 72.01 (d; C-2), 132.03 (s; C-3), 124.47 (d; C-4), 25.86 (t; C-5), 38.82 (d; C-6), 144.87 (s; C-7), 113.98 (t; C-8), 35.43 (t; C-9), 20.42 (q; C-10), 171.77 (s; C-11), 157.09 (d; 2C, C-13 and C-15), 116.48 (d; C-14). HR-MS: 278.1080 (*M*^+^, C_14_H_18_O_2_N_2_S; calc. 278.1084).

**(1*R*,2*R*,6*S*)-6-(3-(Benzo[*d*]oxazol-2-ylthio)prop-1-en-2-yl)-3-methylcyclohex-3-ene-1,2-diol** (**25**). Method A. Yield: 39%. ${\left[\alpha \right]}_{D}^{26.3}$ = +48.9 (*c* 0.090, CHCl_3_). ^1^H-NMR (CDCl_3_, δ_H_): 1.78–1.81 (m, 3H, 3H-10), 2.10 (dm, 1H, ^2^J 17.8 Hz, H-5e), 2.28–2.36 (m, 1H, H-5a), 2.72–2.77 (m, 1H, H-6a), 3.96 (br.d., 1H, J_2,1_ 3.8 Hz, H-2), 4.02 (d, 1H, ^2^J 15.0 Hz, H-9), 4.05 (d, 1H, ^2^J 15.0 Hz, H-9′), 4.07 (dd, 1H, J_1,2_ 3.8, J_1,6a_ 2.5 Hz, H-1), 5.13 (br.s., 1H, H-8), 5.42 (br.s., 1H, H-8′), 5.58–5.62 (m, 1H, H-4), 7.22 (td, 1H, J_15,14_ = J_15,16_ = 7.7, J_15,13_ 1.3 Hz, H-15), 7.25 (td, 1H, J_14,13_ = J_14,15_ = 7.7, J_14,16_ 1.3 Hz, H-14), 7.40 (dd, 1H, J_16,15_ 7.7, J_16,14_ 1.3 Hz, H-16), 7.55 (dd, 1H, J_13,14_ 7.7, J_13,15_ 1.3 Hz, H-16). ^13^C-NMR, δ_C_: 72.46 (d; C-1), 72.24 (d; C-2), 132.28 (s; C-3), 124.22 (d; C-4), 25.83 (t; C-5), 38.91 (d; C-6), 144.26 (s; C-7), 114.89 (t; C-8), 36.66 (t; C-9), 20.34 (q; C-10), 165.13 (s; C-11), 151.65 (s; C-12), 118.08 (d; C-13), 124.35 (d; C-14), 124.04 (d; C-15), 109.84 (d; C-16), 141.10 (d; C-17). HR-MS: 317.1077 (*M*^+^, C_17_H_19_O_3_NS; calc. 317.1080).

**(1*R*,2*R*,6*S*)-3-Methyl-6-(3-(phenylamino)prop-1-en-2-yl)cyclohex-3-ene-1,2-diol** (**26**). Method B. Yield: 67%. ${\left[\alpha \right]}_{D}^{20}$ = −29.6 (*c* 1.12, CHCl_3_). ^1^H-NMR (CDCl_3_, δ_H_): 1.77–1.81 (m, 3H, 3H-10), 2.02 (dm, 1H, ^2^J 17.2 Hz, H-5e), 2.24–2.35 (m, 1H, H-5a), 2.63 (br.dd, 1H, J_6a,5a_ 10.4, J_6a,5e_ 5.5 Hz, H-6a), 3.73 (d, 1H, ^2^J 14.8 Hz, H-9), 3.79 (d, 1H, ^2^J 14.8 Hz, H-9′), 3.84–3.89 (m, 2H, H-1 and H-2), 5.09 (s, 1H, H-8), 5.22 (br.s., 1H, H-8′), 5.60–5.64 (m, 1H, H-4), 6.64 (td, 2H, J_12,13_ = J_16,15_ = 8.4, J_12,14_ = J_16,14_ = 1.0 Hz, H-12 and H-16), 6.74 (tt, 1H, J_14,13_ = J_14,15_ = 7.4, J_14,12_ = J_14,16_ = 1.0 Hz, H-14), 7.40 (dd, 2H, J_13,12_ = J_15,16_ = 8.4, J_13,14_ = J_15,14_ = 7.4 Hz, H-13 and H-15). ^13^C-NMR, δ_C_: 72.17 (d; C-1), 72.01 (d; C-2), 131.84 (s; C-3), 124.83 (d; C-4), 25.41 (t; C-5), 38.10 (d; C-6), 147.15 (s; C-7), 113.60 (t; C-8), 48.90 (t; C-9), 20.57 (q; C-10), 147.66 (s; C-11), 113.60 (d; 2C, C-12 and C-16), 129.10 (d; 2C, C-13 and C-15), 118.25 (d; C-14). HR-MS: 259.1563 (*M*^+^, C_16_H_21_O_2_N; calc. 259.1567).

**(1*R*,2*R*,6*S*)-3-Methyl-6-(3-(*p*-tolylamino)prop-1-en-2-yl)cyclohex-3-ene-1,2-diol** (**27**). Method B. Yield: 44%. ${\left[\alpha \right]}_{D}^{20}$ = −36.9 (*c* 0.363, CHCl_3_). ^1^H-NMR (CDCl_3_, δ_H_): 1.77–1.80 (m, 3H, 3H-10), 1.99 (dm, 1H, ^2^J 17.7 Hz, H-5e), 2.22 (s, 3H, 3H-17), 2.24–2.35 (m, 1H, H-5a), 2.63 (br.dd, 1H, J_6a,5a_ 10.3, J_6a,5e_ 5.3 Hz, H-6a), 3.10 (br.s., 3H, 2OH, NH), 3.68 (d, 1H, ^2^J 14.4 Hz, H-9), 3.75 (d, 1H, ^2^J 14.4 Hz, H-9′), 3.83–3.87 (m, 2H, H-1 and H-2), 5.08 (s, 1H, H-8), 5.19 (br.s., 1H, H-8′), 5.59–5.63 (m, 1H, H-4), 6.58 (br.d., 2H, J_12,13_ = J_16,15_ = 8.2 Hz, H-12 and H-16), 6.97 (br.d., 2H, J_13,12_ = J_15,16_ = 8.2 Hz, H-13 and H-15). ^13^C-NMR, δ_C_: 72.32 (d; C-1), 72.00 (d; C-2), 131.90 (s; C-3), 124.71 (d; C-4), 25.49 (t; C-5), 38.52 (d; C-6), 147.41 (s; C-7), 113.95 (t; C-8), 49.38 (t; C-9), 20.56 (q; C-10), 145.20 (s; C-11), 114.09 (d; 2C, C-12 and C-16), 129.58 (d; 2C, C-13 and C-15), 127.84 (s; C-14), 20.26 (q; C-17). HR-MS: 273.1727 (*M*^+^, C_17_H_23_O_2_N; calc. 273.1723).

**(1*R*,2*R*,6*S*)-6-(3-(2,4-Dimethylphenylamino)prop-1-en-2-yl)-3-methylcyclohex-3-ene-1,2-diol** (**28**). Method B. Yield: 63%. ${\left[\alpha \right]}_{D}^{24.6}$ = −24.0 (*c* 0.500, CHCl_3_). ^1^H-NMR (CDCl_3_, δ_H_): 1.79–1.81 (m, 3H, 3H-10), 2.03 (dm, 1H, ^2^J 17.8 Hz, H-5e), 2.13 (s, 3H, 3H-17), 2.21 (s, 3H, 3H-18), 2.28–2.35 (m, 1H, H-5a), 2.66 (br.dd, 1H, J_6a,5a_ 10.5, J_6a,5e_ 5.3 Hz, H-6a), 3.75 (d, 1H, ^2^J 14.4 Hz, H-9), 3.81 (d, 1H, ^2^J 14.4 Hz, H-9′), 3.87 (br.d., 1H, J_2,1_ 3.4 Hz, H-2), 3.89 (dd, 1H, J_1,2_ 3.4, J_1,6a_ 1.9 Hz, H-1), 5.11 (br.s., 1H, H-8), 5.20–5.22 (m, 1H, H-8′), 5.61–5.64 (m, 1H, H-4), 6.56 (d, 1H, J_16,15_ 8.1 Hz, H-16), 6.88 (br.s., 1H, H-13), 6.90 (d, 1H, J_15,16_ 8.1 Hz, H-15). ^13^C-NMR, δ_C_: 72.35 (d; C-1), 72.10 (d; C-2), 131.92 (s; C-3), 124.82 (d; C-4), 25.57 (t; C-5), 38.59 (d; C-6), 147.46 (s; C-7), 113.85 (t; C-8), 49.01 (t; C-9), 20.55 (q; C-10), 143.12 (s; C-11), 122.98 (s; C-12), 130.94 (d; C-13), 127.34 (s; C-14), 127.22 (d; C-15), 111.21 (d; C-16), 17.33 (q; C-17), 20.23 (q; C-18). HR-MS: 287.1884 (*M*^+^, C_18_H_25_O_2_N; calc. 287.1880).

**(1*R*,2*R*,6*S*)-6-(3-(Diethylamino)prop-1-en-2-yl)-3-methylcyclohex-3-ene-1,2-diol** (**29**). Method B. Yield: 66%. ${\left[\alpha \right]}_{D}^{24.1}$ = +2.5 (*c* 1.28, CHCl_3_). ^1^H-NMR (CDCl_3_, δ_H_): 1.08 (t, 6H, J_12,11_ = J_12,11′_ = J_14,13_= J_14,13′_ = 7.2 Hz, 3H-12 and 3H-14), 1.77–1.79 (m, 3H, 3H-10), 1.94 (dm, 1H, ^2^J 17.2 Hz, H-5e), 2.28 (br.dd, 1H, ^2^J 17.2, J_5a,6a_ 10.0 Hz, H-5a), 2.55 (dq, 2H, ^2^J 14.0, J_11,12_ = J_13,14_ = 7.2 Hz, H-11 and H-13), 2.72–2.79 (m, 3H, H-6a, H-11′ and H-13′), 2.87 (d, 1H, ^2^J 12.8 Hz, H-9), 3.29 (br. d., 1H, ^2^J 12.8 Hz, H-9′), 3.70 (br. dd., 1H, J_1,2_ 3.8, J_1,6a_ 2.0 Hz, H-1), 3.83 (br.d., 1H, J_2,1_ 3.8 Hz, H-2), 4.98 (br.s., 1H, H-8), 5.16–5.18 (m, 1H, H-8′), 5.54–5.57 (m, 1H, H-4). ^13^C-NMR, δ_C_: 73.28 (d; C-1), 72.43 (d; C-2), 132.67 (s; C-3), 123.91 (d; C-4), 26.53 (t; C-5), 42.56 (d; C-6), 145.90 (s; C-7), 119.52 (t; C-8), 57.07 (t; C-9), 20.47 (q; C-10), 45.26 (t; 2C, C-11 and C-13), 9.82 (q; 2C, C-12 and C-14). HR-MS: 238.1804 (*M*^+^-H, C_14_H_24_O_2_N; calc. 238.1802).

**1-(2-(2-((1*S*,5*R*,6*R*)-5,6-Dihydroxy-4-methylcyclohex-3-enyl)allylamino)phenyl)ethanone** (**30**). Method B. Yield: 21%. ${\left[\alpha \right]}_{D}^{24.1}$ = −54.5 (*c* 0.22, CHCl_3_). ^1^H-NMR (CDCl_3_, δ_H_): 1.79–1.81 (m, 3H, 3H-10), 1.94 (br.s., 2H, 2OH), 2.05 (dm, 1H, ^2^J 17.9 Hz, H-5e), 2.26–2.34 (m, 1H, H-5a), 2.55–2.57 (m, 1H, H-6a), 2.56 (s, 3H, 3H-18), 3.86–3.96 (m, 4H, H-1, H-2 and 2H-9), 5.07 (s, 1H, H-8), 5.20 (s, 1H, H-8′), 5.62–5.65 (m, 1H, H-4), 6.58 (dd, 1H, J_14,15_ 8.0, J_14,13_ 7.0 Hz, H-14), 6.63 (d, 1H, J_12,13_ 8.6 Hz, H-12), 7.31 (dd, 1H, J_13,12_ 8.6, J_13,14_ 7.0 Hz, H-13), 7.73 (br.d., 1H, J_15,14_ 8.0 Hz, H-15), 9.07 (br.s., 1H, NH). ^13^C-NMR, δ_C_: 71.83 and 72.05 (2d, 2C; C-1 and C-2), 131.81 (s; C-3), 124.95 (d; C-4), 25.29 (t; C-5), 37.28 (d; C-6), 145.76 (s; C-7), 112.04 (t; C-8), 47.28 (t; C-9), 20.57 (q; C-10), 150.84 (s; C-11), 111.95 (d; C-12), 134.95 (d; C-13), 114.31 (d; C-14), 132.62 (d; C-15), 117.67 (s; C-16), 201.04 (s; C-17), 27.83 (q; C-18). HR-MS: 301.1677 (*M*^+^, C_18_H_23_O_3_N; calc. 301.1673).

**1-(3-(2-((1*S*,5*R*,6*R*)-5,6-Dihydroxy-4-methylcyclohex-3-enyl)allylamino)phenyl)ethanone** (**31**). Method B. Yield: 58%. ${\left[\alpha \right]}_{D}^{26.8}$ = −17.4 (*c* 0.367, CHCl_3_). ^1^H-NMR (CDCl_3_, δ_H_): 1.76–1.79 (m, 3H, 3H-10), 2.02 (dm, 1H, ^2^J 18.0 Hz, H-5e), 2.24–2.32 (m, 1H, H-5a), 2.51 (s, 3H, 3H-18), 2.60 (br.dd, 1H, J_6a,5a_ 9.9, J_6a,5e_ 5.3 Hz, H-6a), 3.81 (s, 2H, 2H-9), 3.89 (br.d., 1H, J_2,1_ 3.0 Hz, H-2), 3.92 (dd, 1H, J_1,2_ 3.0, J_1,6a_ 2.0 Hz, H-1), 5.06 (s, 1H, H-8), 5.17 (s, 1H, H-8′), 5.58–5.62 (m, 1H, H-4), 6.78 (br. dd, 1H, J_16,15_ 7.8, J_16,14_ 1.7 Hz, H-16), 7.17 (br.s., 1H, H-12), 7.20 (t, 1H, J_15,14_ = J_15,16_ = 7.8 Hz, H-15), 7.24 (br.d., 1H, J_14,15_ 7.8 Hz, H-14). ^13^C-NMR, δ_C_: 72.17 (d, 2C; C-1 and C-2), 131.95 (s; C-3), 124.78 (d; C-4), 25.54 (t; C-5), 37.69 (d; C-6), 146.55 (s; C-7), 112.76 (t; C-8), 48.51 (t; C-9), 20.47 (q; C-10), 148.05 (s; C-11), 111.84 (d; C-12), 137.86 (d; C-13), 118.08 (d; C-14), 129.15 (d; C-15), 118.02 (s; C-16), 198.90 (s; C-17), 26.55 (q; C-18). HR-MS: 301.1675 (*M*^+^, C_18_H_23_O_3_N; calc. 301.1673).

### 3.2. Biology

#### 3.2.1. Isolation of Mitochondria from Mouse Brain

This study was carried out in accordance with Directive 2010/63/EU and approved by the Ethical Committee of FRC PCPMC RAS (Approval No 87 from 26 December 2023). Male BDF1 (10-week-old) were collected and euthanized by decapitation. Mitochondria were isolated from the mouse brain according to the protocol [54]. The extracted brains were washed in ice-cold medium containing 0.32 M sucrose in K-Na phosphate buffer (pH 7.4). Brain tissues were homogenized in four volumes of the medium and then spun in a centrifuge at 900× *g* at 4 °C for 5 min. The supernatant was collected and then centrifuged at 12,500× *g* at 4 °C for 15 min. The pellets were resuspended and recentrifuged. The crude mitochondrial fraction obtained in the sediment was resuspended in isolation buffer (isotonized with KCl) and frozen in liquid nitrogen. The protein content in mitochondrial suspension was estimated using the Lowry protein assay [55].

#### 3.2.2. MAO Activity Assay

Fluorescence assay was used to study the MAO activity. The method is based on the original procedure of M. Krajl [56], using clorgiline and (*R*)-deprenyl as positive controls of MAO-A and MAO-B selective inhibition, respectively. A Cary Eclipse fluorometer (Agilent, Santa Clara, CA, USA) was used. Clorgyline and selegiline (both at a concentration of 0.25 µM) were used as selective irreversible inhibitors to assay two isoforms of MAO. Mitochondrial protein concentration was 1 mg/mL. A few minutes of preincubation of the selective inhibitors and the mitochondria was made, followed by the simultaneous addition of tested compounds (10 μM and 100 μM). Then, kynuramine, a non-selective substrate, was added at concentrations of 90 mM for MAO-A and 60 mM for MAO-B. Samples were subjected to 30 min incubation at 37 °C. After that, the reaction was terminated by addition of trichloroacetic acid (10% *w/v*). After further centrifugation at 3000× *g* for 15 min, the supernatant was mixed with 1M NaOH. The content of 4-hydroxyquinoline was then estimated at 380 nm. The excitation wavelength was 315 nm. MAO-A and MAO-B activity was signified as concentration of 4-hydroxyquinoline formed per mg of protein per minute.

#### 3.2.3. Statistical Analysis

All biological experiments were performed in triplicate and repeated several times. Standard deviation (SD) was used to indicate dispersion of the data from the mean and the results were expressed as mean ± SD. Statistical analysis was performed using Student’s *t*-test (Microsoft Excel), and the *p*-values were provided (*—*p* < 0.05; **—*p* < 0.01; ***—*p* < 0.001).

## 4. Conclusions

This study focused on the synthesis and investigation of the effect of the 9-derivatives of (1*R*,2*R*,6*S*)-3-methyl-6-(prop-1-en-2-yl)cyclohex-3-en-1,2-diol (Prottremine) on the catalytic activity of MAO-A and -B enzymes. Prottremine is known to exhibit high antiparkinsonian activity and is in the second phase of clinical trials. The synthesis of the new derivatives was conducted in two steps. Bromide **11** was synthesized following the improved technique, yielding 61%. Its interaction with *N-* and *S-*nucleophiles resulted in the formation of target products **18**–**31** with yields ranging from 24% to 67%.

MAO-B inhibitors are widely used as part of combination drug therapy in the early stages of PD to slow Levodopa degradation. For the synthesized compounds, their effect on the catalytic activity of MAO-A and -B enzymes in murine mitochondrial suspension at concentrations of 10 and 100 μM was investigated.

Regarding MAO-A, the tested compounds showed no or low activity, with an IC_50_ value of 178 ± 44 µM for compound **28**. The results appear more promising in terms of MAO-B activity, which is more relevant when it comes to antiparkinsonian activity. In particular, Prottremine showed little inhibitory activity when the dose was reduced to 10 µM. However, its sulfur-containing derivatives **18**, **19**, and **21**–**25**, as well as the aniline **27**, **28**, and **31** derivatives exhibited higher efficacy, with compound 30 demonstrating an IC_50_ of 95 ± 5 µM.

According to the docking results, for the MAO-B enzyme, the minimum binding energy was observed for compound **30,** which also exhibited the highest activity in in vitro experiments. It is important to note that relative to the MAO-B enzyme, the synthesized set of compounds showed a good correlation between the molecular docking data and the measured activity towards the enzyme (R^2^ = 0.685, Figure 3). As for the MAO-A enzyme, the correlation has not been that high (R^2^ = 0.343, Figure 3). However, compound **28** being the most active compound in in vitro experiments has demonstrated the second lowest affinity value (−9.10) based on the docking results, only slightly behind compound **27** (−9.30). The obtained data indicate that some Prottremine derivatives may exhibit antiparkinsonian activity mediated, among other action pathways, by MAO-B inhibition.

## Figures and Tables

**Table 1:** Inhibition of MAO-A and -B enzymes by Prottremine and its derivatives.

Structure	N	Concentration, µM	MAO-A, % ± SD	MAO-B, % ± SD
Control			100 ± 2	100 ± 3
DMSO		1.4%	107 ± 5	99 ± 3
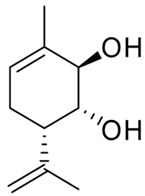	**10**	100	101 ± 2	93 ± 1 *
		10	103 ± 2	95 ± 1 *
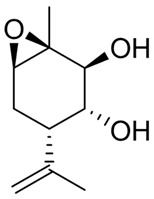	**14**	100	99 ± 4	93 ± 0.5 *
		10	104 ± 3	96 ± 2
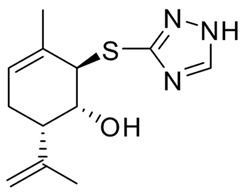	**15**	100	102 ± 4	99 ± 3
		10	104 ± 4	97 ± 3
Control			100 ± 4	100 ± 1
DMSO		1.4%	105 ± 4	99 ± 3
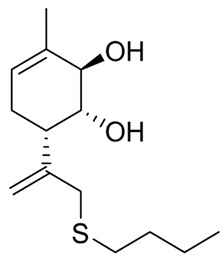	**18**	100	96 ± 1	96 ± 1 *
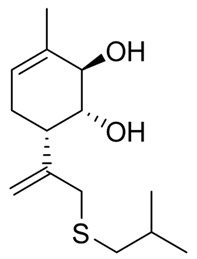	**19**	100	102 ± 3	97 ± 0.4 *
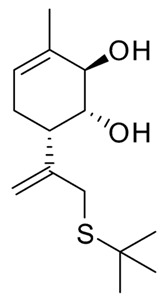	**20**	100	95 ± 6	98 ± 0.6
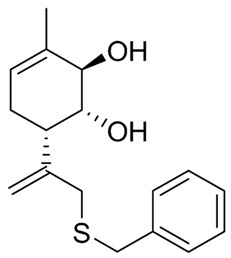	**21**	100	98 ± 2	82 ± 2 ***
		10	-	97 ± 2
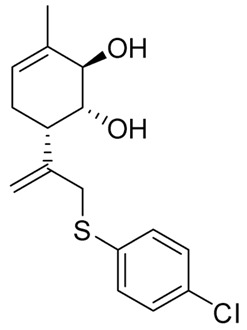	**22**	100	88 ± 3 **	83 ± 2 **
		10	-	97 ± 2
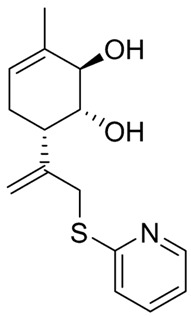	**23**	100	99 ± 4	91 ± 1 **
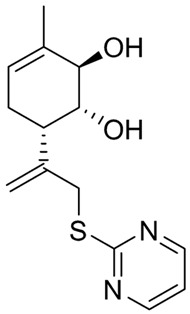	**24**	100	96 ± 2 *	91 ± 2 ^**^
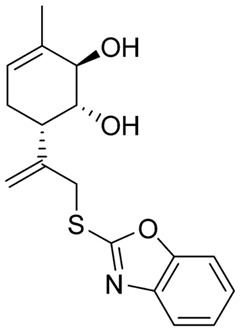	**25**	100	97 ± 4	91 ± 1 **
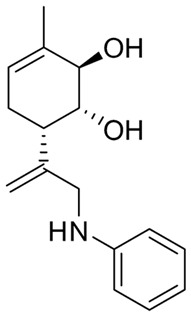	**26**	100	93 ± 5	91 ± 3 *
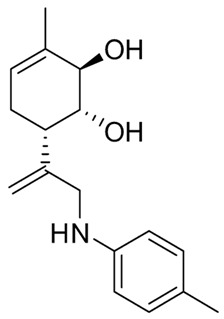	**27**	100	89 ± 2	66 ± 0.1 ***
		10	-	94 ± 2 *
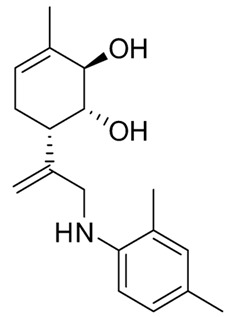	**28**	100	56 ± 3 ***	75 ± 2 **
		10	99 ± 3	98 ± 0.4
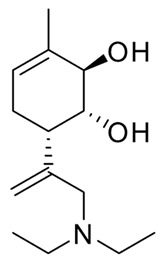	**29**	100	111 ± 2 **	100 ± 1
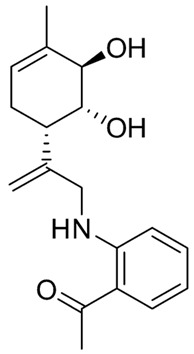	**30**	100	57 ± 1 ***	42 ± 1 ***
		10	106 ± 2	94 ± 2 *
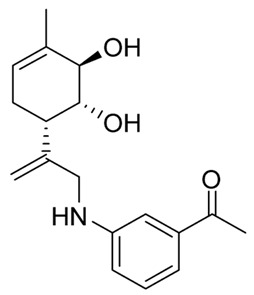	**31**	100	76 ± 3 **	66 ± 2 ***
		10	99 ± 3	101 ± 1

*—*p* < 0.05; **—*p* < 0.01; ***—*p* < 0.001 from control by *t*-test.

**Table 2:** Comparison of the protein sequences of MAO-A and -B enzymes from *Homo sapiens* (human enzymes) and *Mus musculus* (murine enzymes).

Enzyme	Sequence Identity with the Human Homolog	Structural RMSD, Å	Key Amino Acids in the Murine Enzyme Active Center for Murine Enzyme
MAO-A	88.1%	0.375	Y69, I180, N181, F208, Q215, C323, I335, L337, F352, Y407, Y444
MAO-B	89%	0.313	Y60, P104, I164, F168, L171, C172, I198, I199, G206, V316, Y326, P343, Y398, Y435

**Table 3:** Minimum binding affinity (kcal/mol) to MAO enzymes.

Compound	MAO-A	MAO-B
DMSO	−2.40	−2.50
**10**	−6.60	−5.70
**14**	−6.50	−5.30
**15**	−7.10	−7.00
**18**	−7.50	−5.40
**19**	−7.80	−5.10
**20**	−7.60	−5.60
**21**	−8.60	−6.40
**22**	−8.40	−5.80
**23**	−8.20	−5.90
**24**	−7.90	−5.80
**25**	−9.10	−5.90
**26**	−8.80	−6.80
**27**	−9.30	−6.90
**28**	−9.10	−6.80
**29**	−7.30	−5.50
**30**	−9.00	−8.60
**31**	−8.30	−8.30

## Data Availability

The original contributions presented in this study are included in the article/Supplementary Materials. Further inquiries can be directed to the corresponding author(s).

## Supplementary Materials

The supporting information can be downloaded at: https://www.mdpi.com/article/10.3390/ijms26010097/s1.
