# Supplementary material for: Inhibitory Activity of N- and S-Functionalized Monoterpene Diols Towards Monoamine Oxidases A and B
Source: Int J Mol Sci. 2024 Dec 26;26(1):97. doi: 10.3390/ijms26010097 (PMC11720030; doi:10.3390/ijms26010097)

SUPPORTING INFORMATION

Inhibitory Activity of *N*- and *S*-Functionalized Monoterpene Diols Towards Monoamine Oxidases A and B

^{1}H NMR spectrum of (1R,2R,6S)-6-(3-(butylthio)prop-1-en-2-yl)-3-methylcyclohex-3-ene-1,2-diol (**18**).

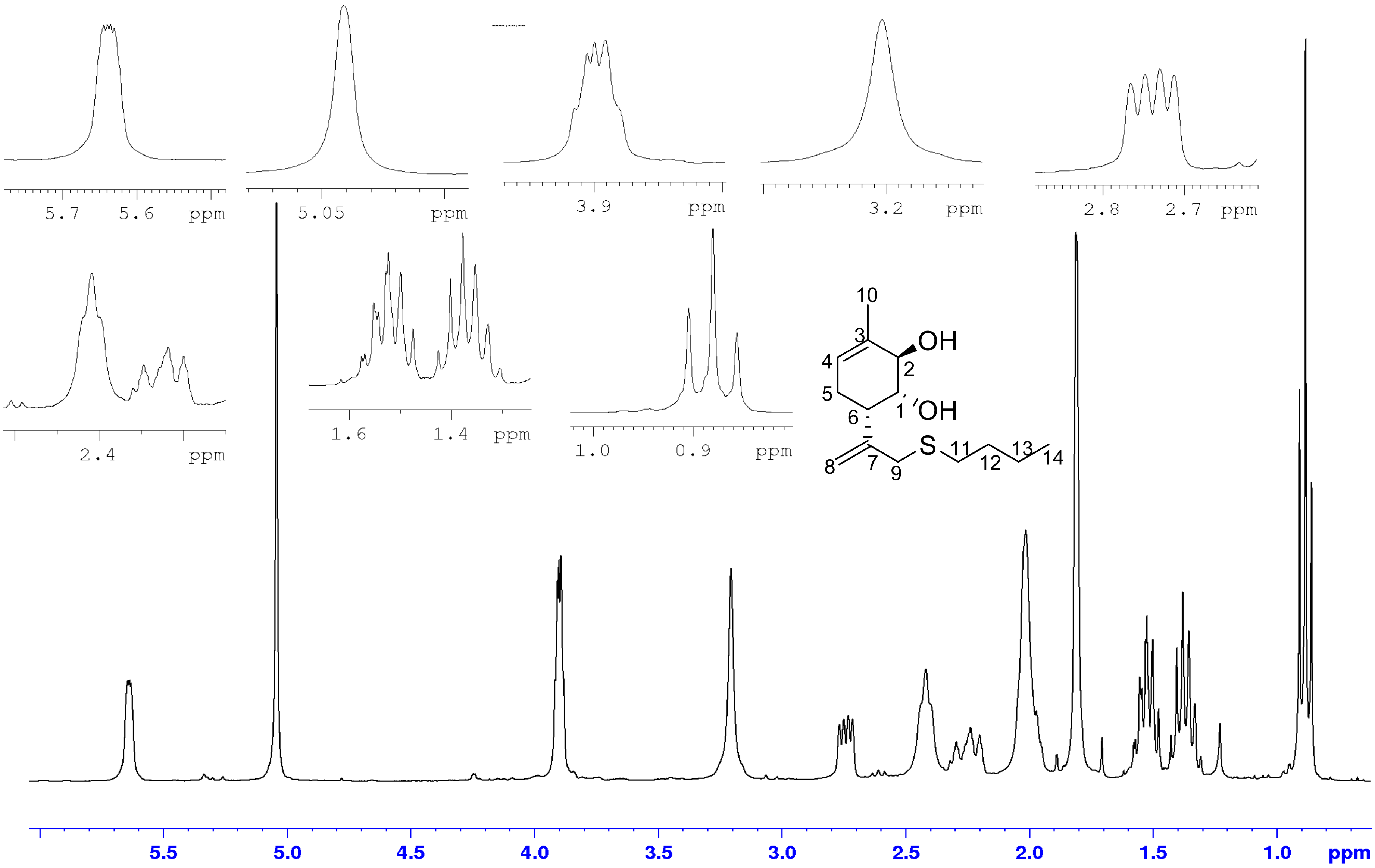

J-modulated ^{13}C NMR spectrum of (1R,2R,6S)-6-(3-(butylthio)prop-1-en-2-yl)-3-methylcyclohex-3-ene-1,2-diol (**18**).

AV-600, 13C-jmod, Ardashov,
A 1056-1-1 in CDCl3, ~20mg

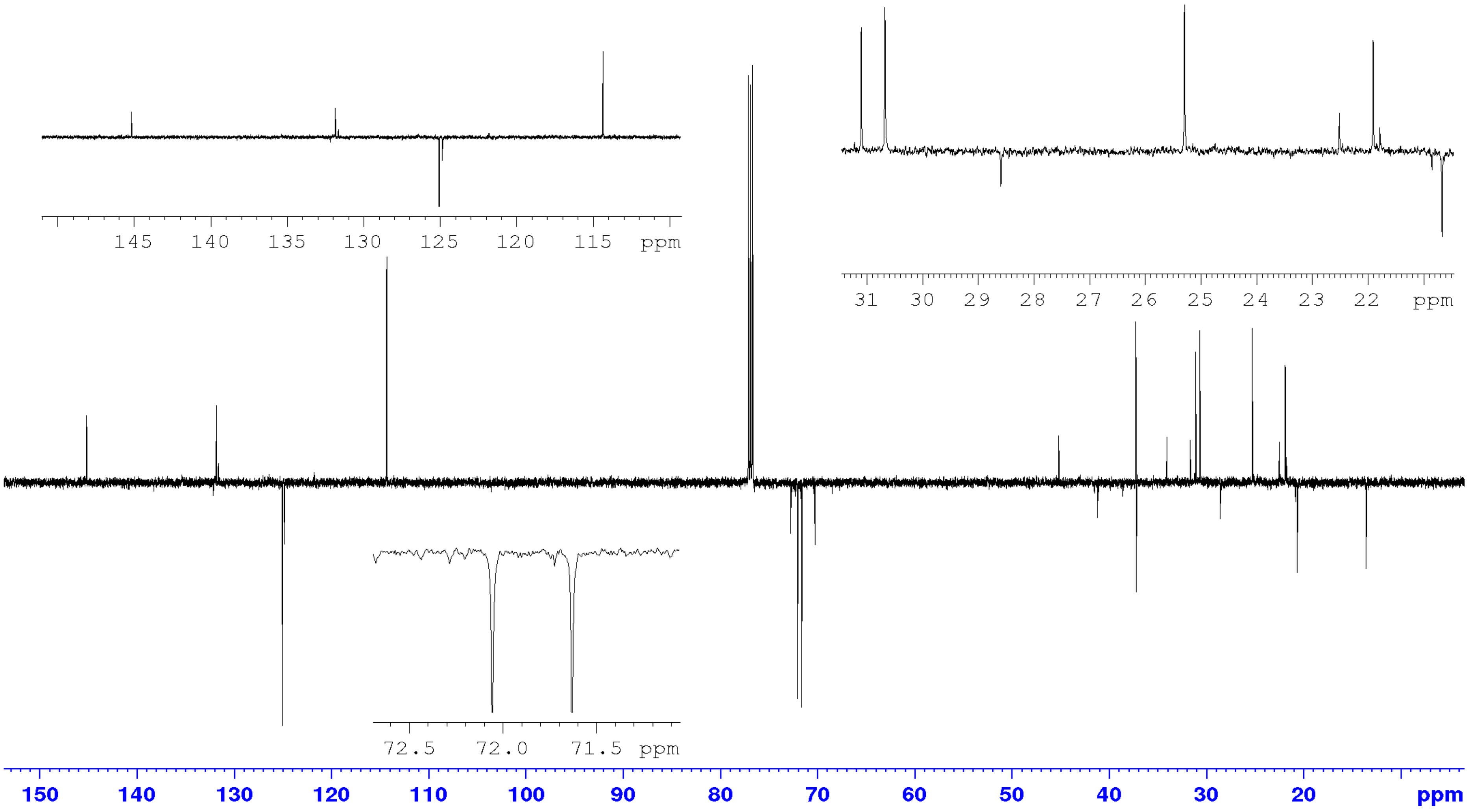

^{1}H-^{1}H 2D homonuclear correlation (COSY) spectrum of (1R,2R,6S)-6-(3-(butylthio)prop-1-en-2-yl)-3-methylcyclohex-3-ene-1,2-diol (**18**).

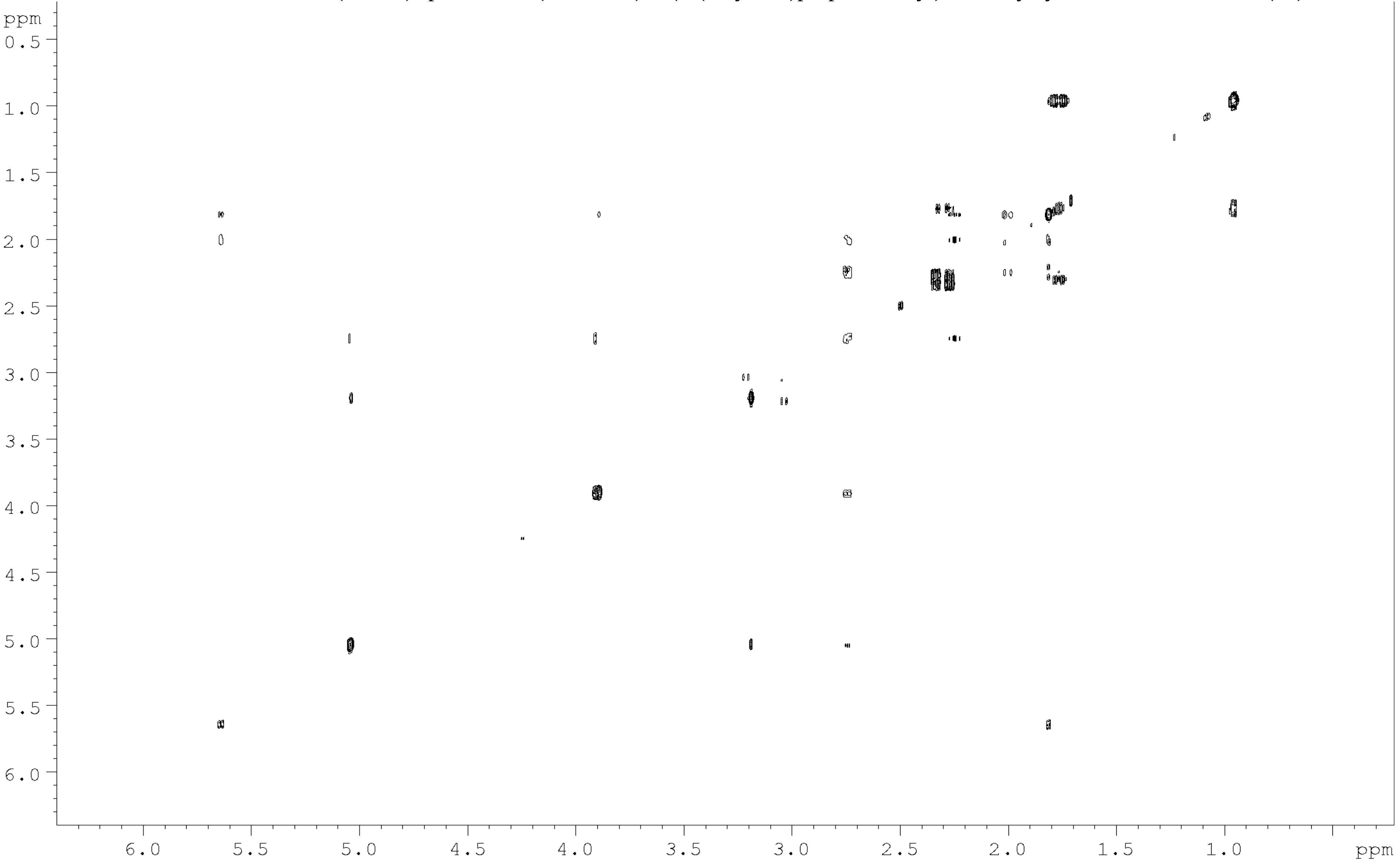

HXCO ^{13}C-^{1}H 2D heteronuclear correlation (C-H COSY) spectrum of (1R,2R,6S)-6-(3-(butylthio)prop-1-en-2-yl)-3-methylcyclohex-3-ene-1,2-diol (**18**).

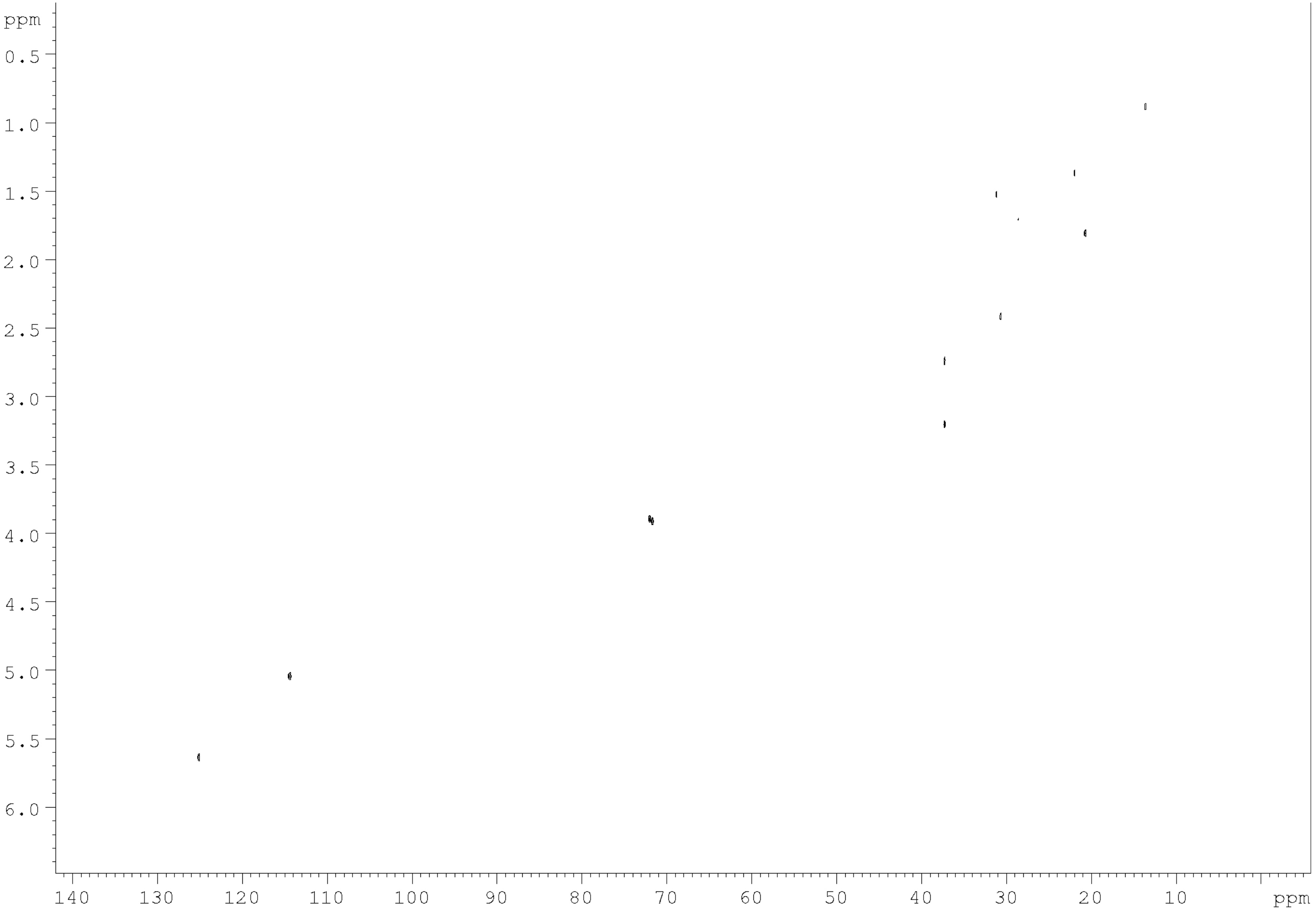

HMBC ^{13}C-^{1}H 2D heteronuclear correlation (C-H COSY) spectrum of (1R,2R,6S)-6-(3-(butylthio)prop-1-en-2-yl)-3-methylcyclohex-3-ene-1,2-diol (**18**).

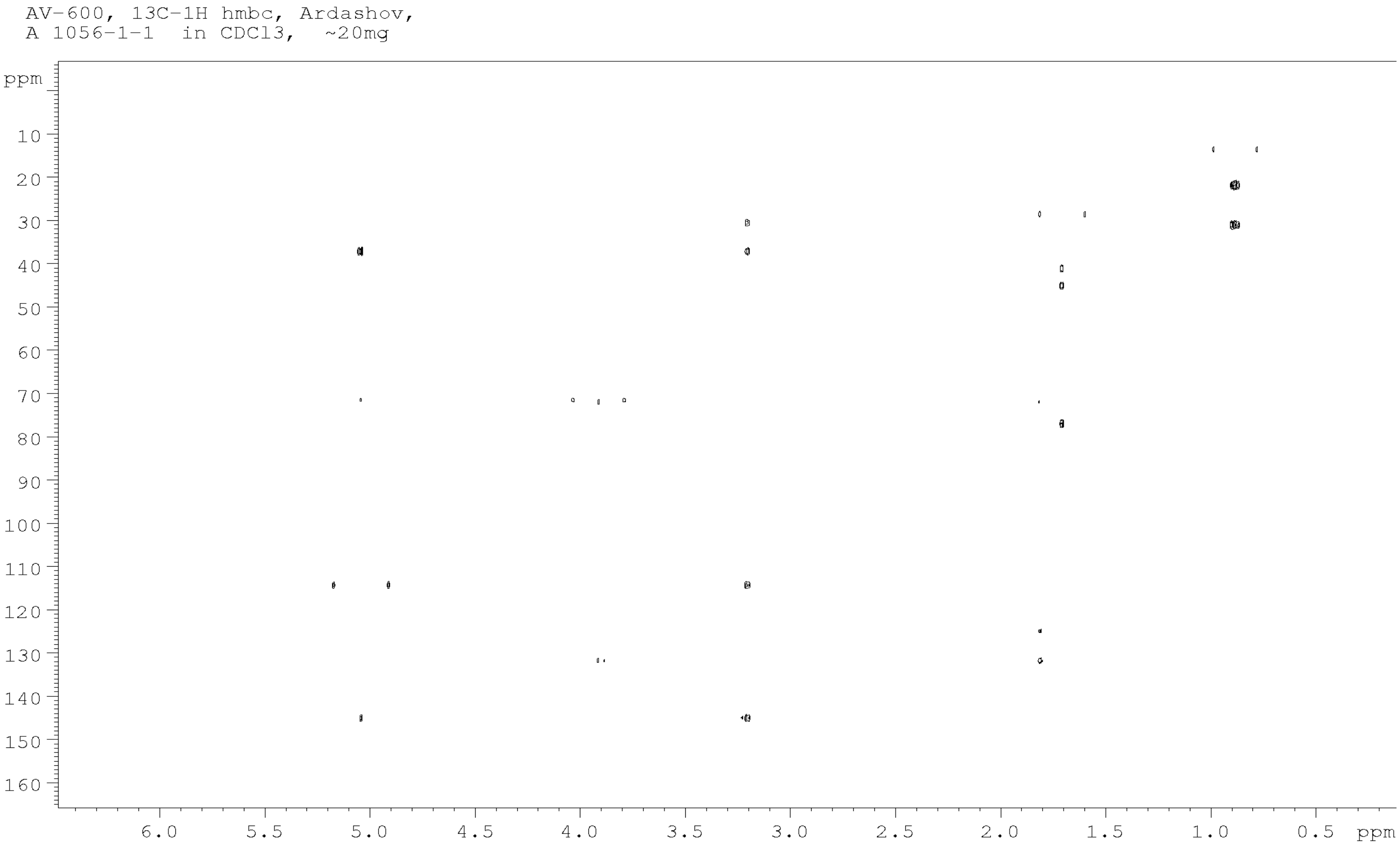

^{1}H NMR spectrum of (1R,2R,6S)-6-(3-(isobutylthio)prop-1-en-2-yl)-3-methylcyclohex-3-ene-1,2-diol (**19**)

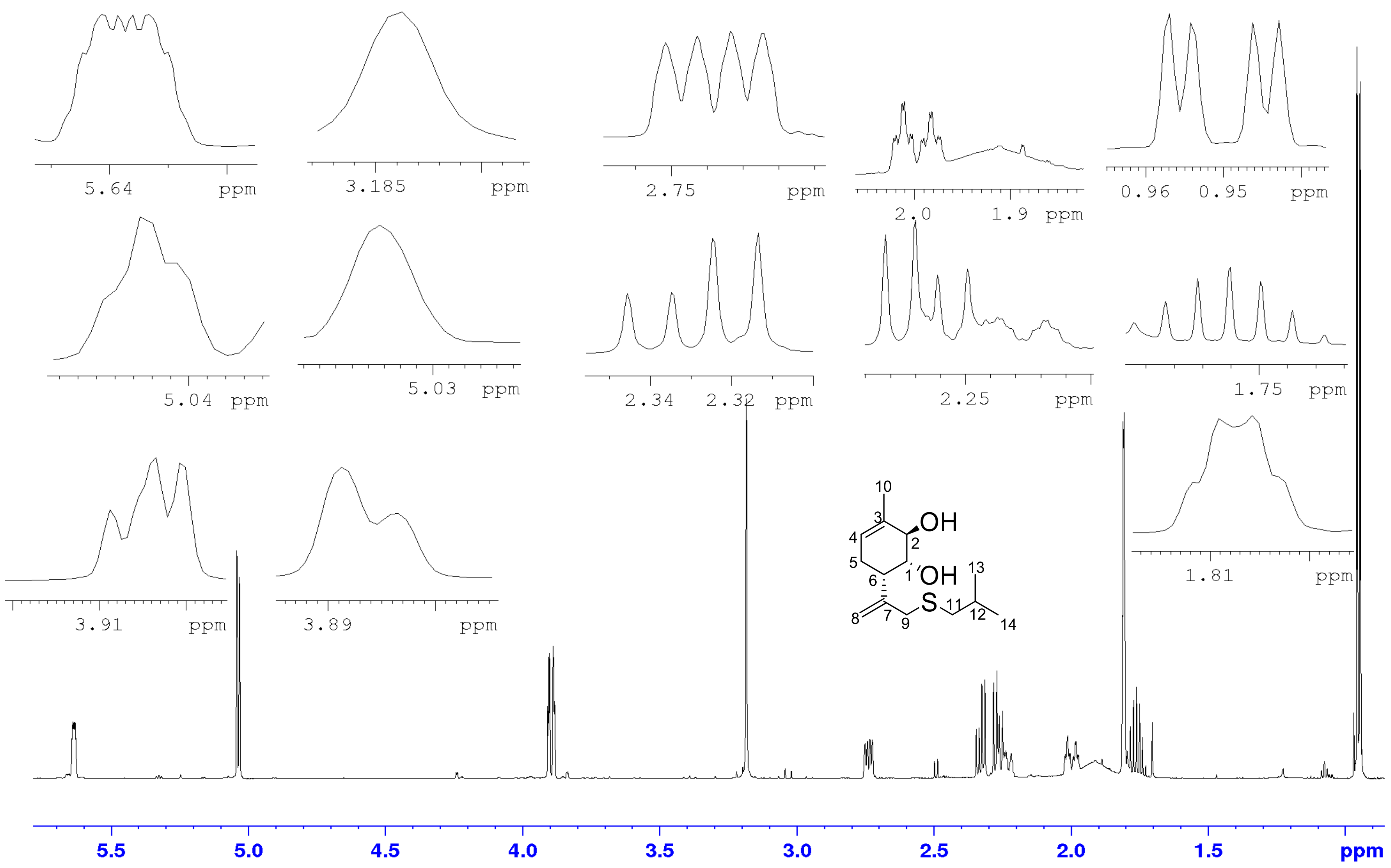

J-modulated ^{13}C NMR spectrum of (1R,2R,6S)-6-(3-(isobutylthio)prop-1-en-2-yl)-3-methylcyclohex-3-ene-1,2-diol (**19**)

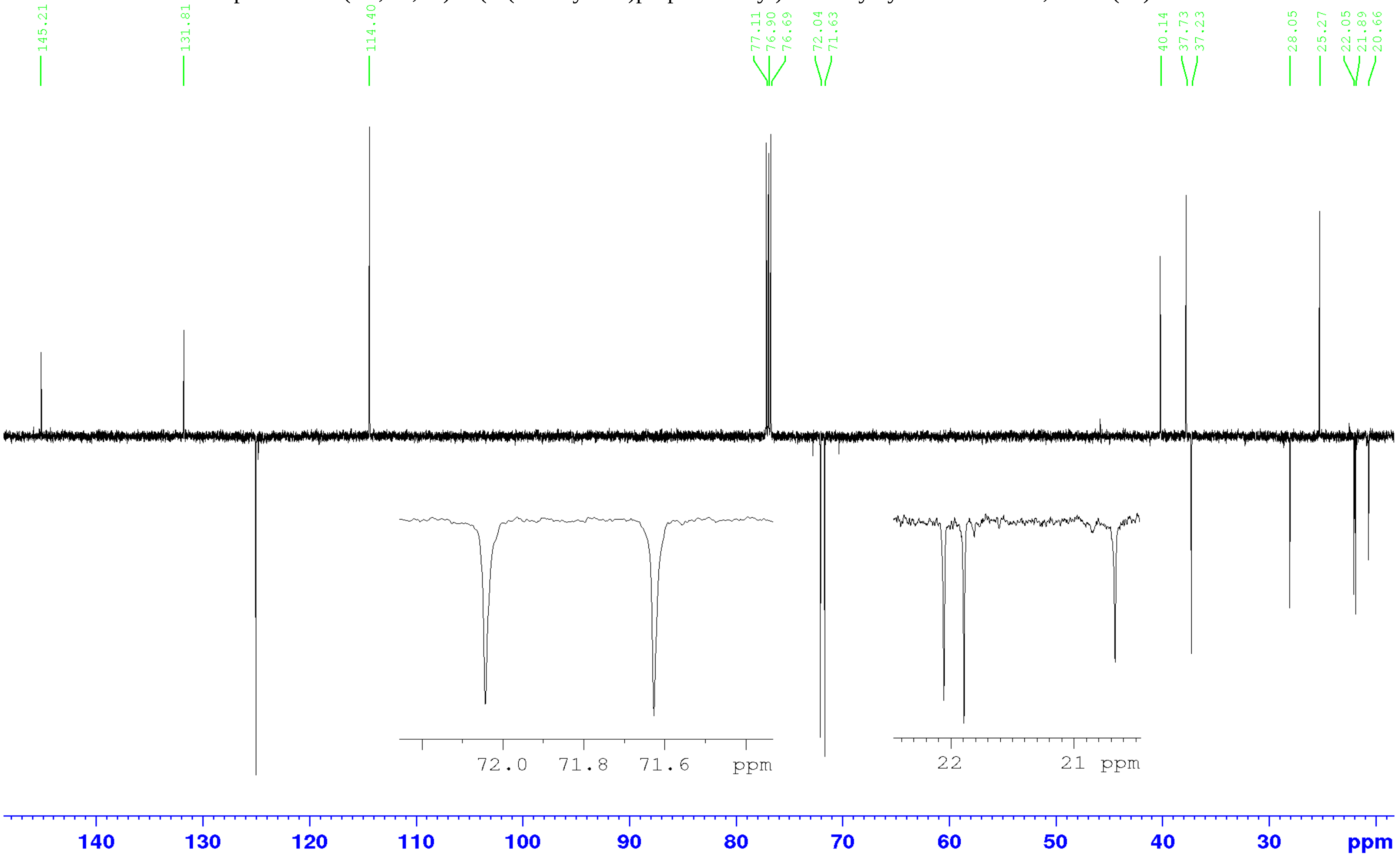

^{1}H-^{1}H 2D homonuclear correlation (COSY) spectrum of (1R,2R,6S)-6-(3-(isobutylthio)prop-1-en-2-yl)-3-methylcyclohex-3-ene-1,2-diol (**19**)

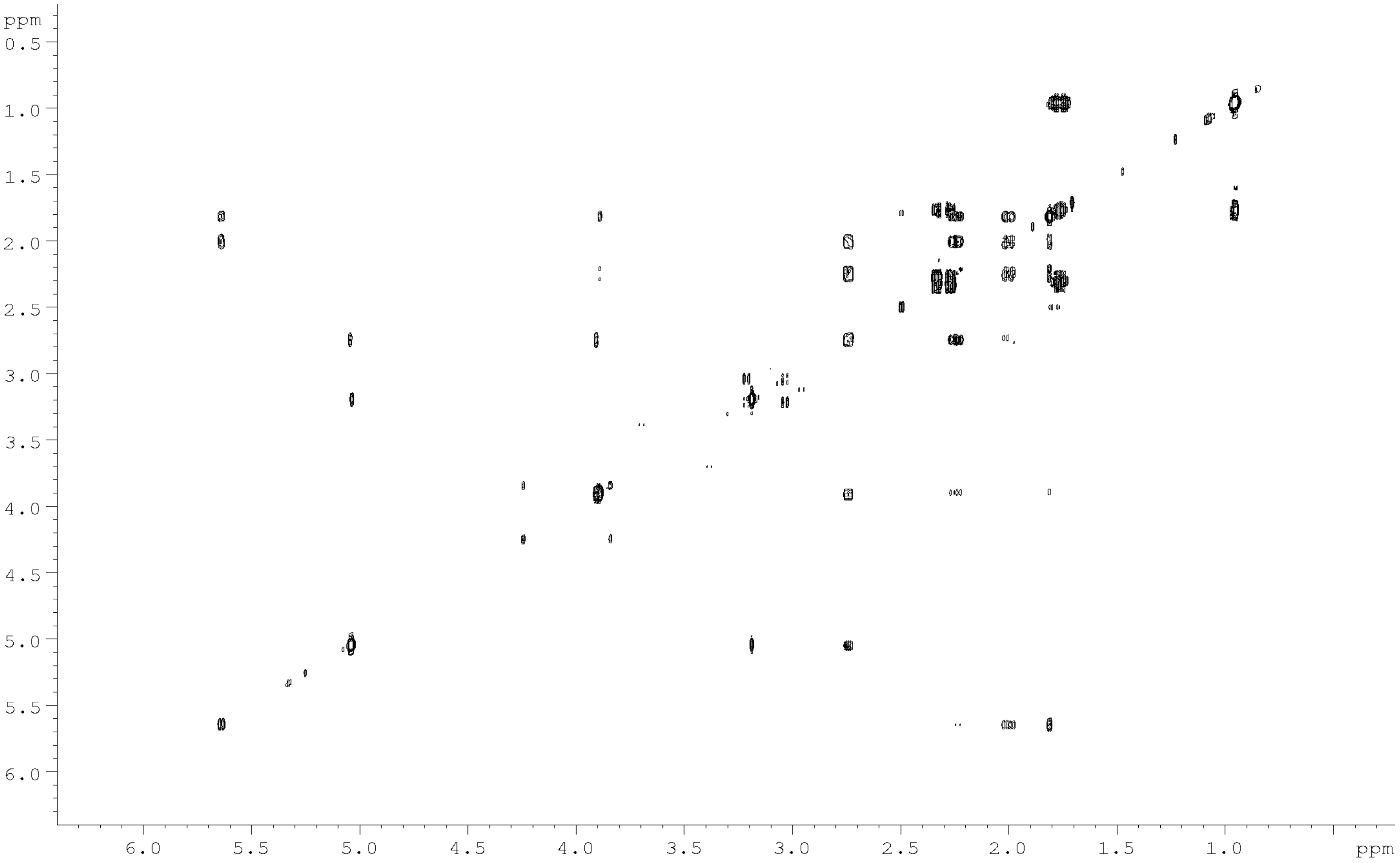

HSQC ^{13}C-^{1}H 2D heteronuclear correlation (C-H COSY) spectrum of (1R,2R,6S)-6-(3-(isobutylthio)prop-1-en-2-yl)-3-methylcyclohex-3-ene-1,2-diol (**19**)

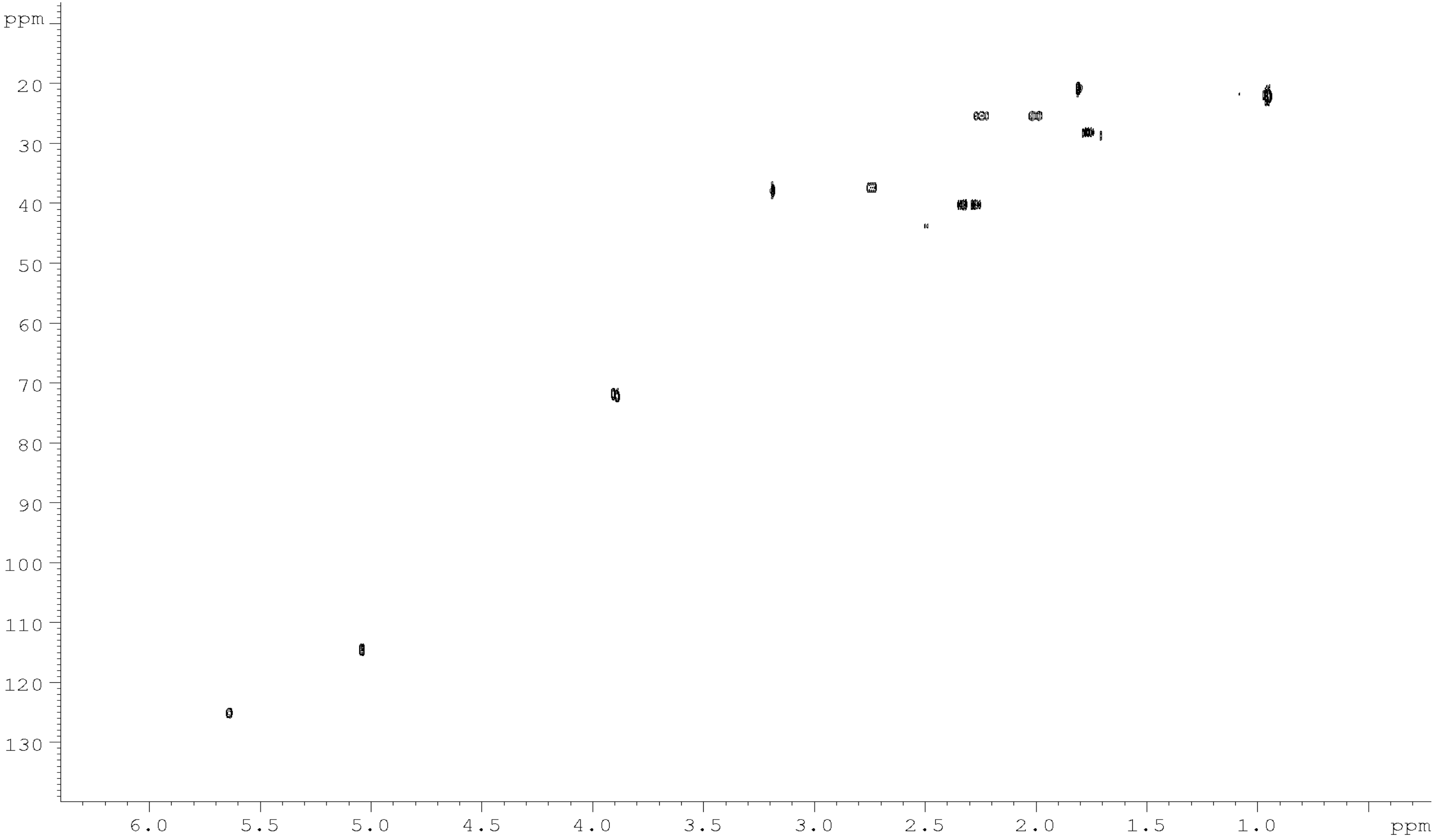

HMBC ^{13}C-^{1}H 2D heteronuclear correlation (C-H COSY) spectrum of (1R,2R,6S)-6-(3-(isobutylthio)prop-1-en-2-yl)-3-methylcyclohex-3-ene-1,2-diol (**19**)

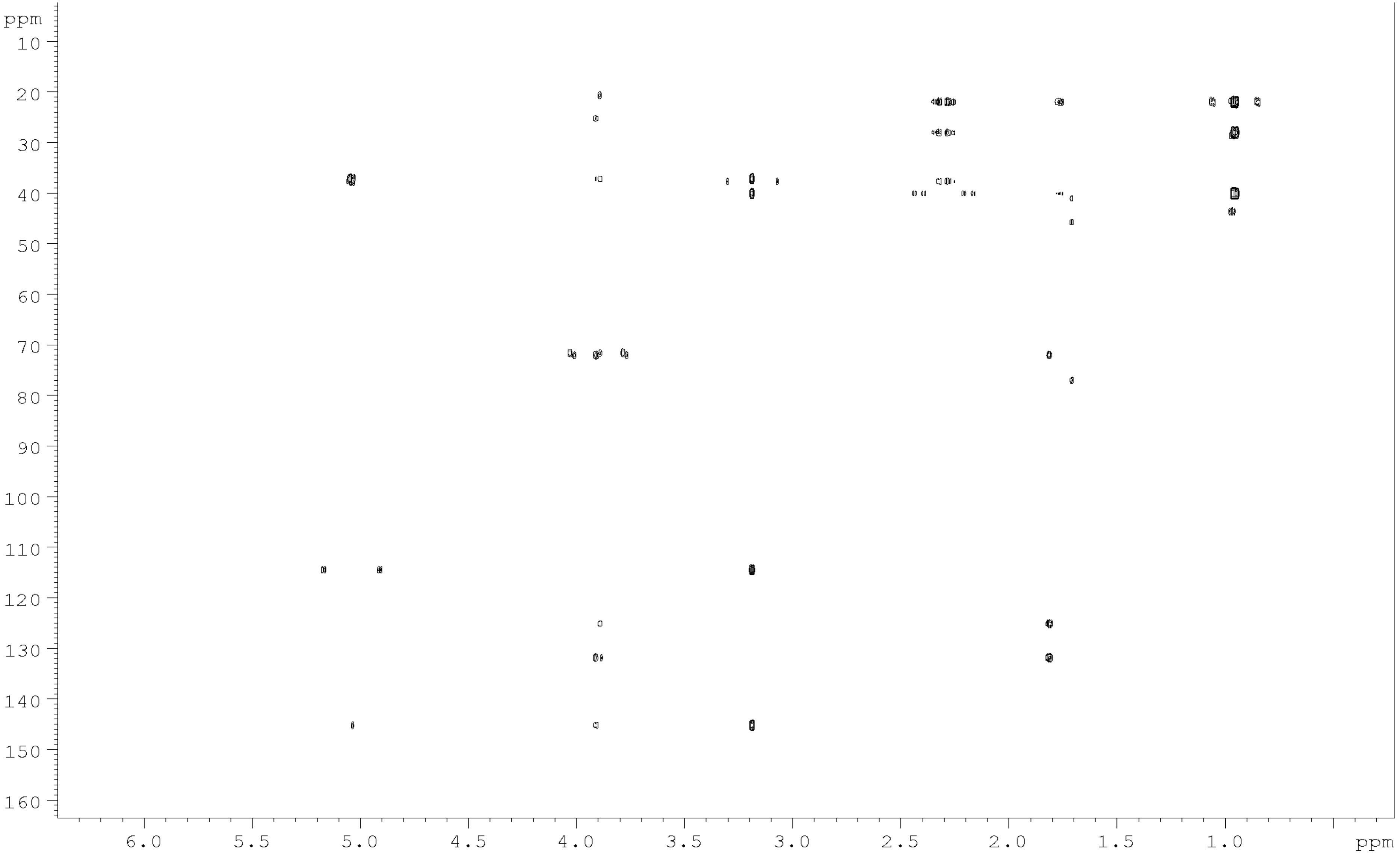

HXCO ^{13}C-^{1}H 2D heteronuclear correlation (C-H COSY) spectrum of (1R,2R,6S)-6-(3-(isobutylthio)prop-1-en-2-yl)-3-methylcyclohex-3-ene-1,2-diol (**19**)

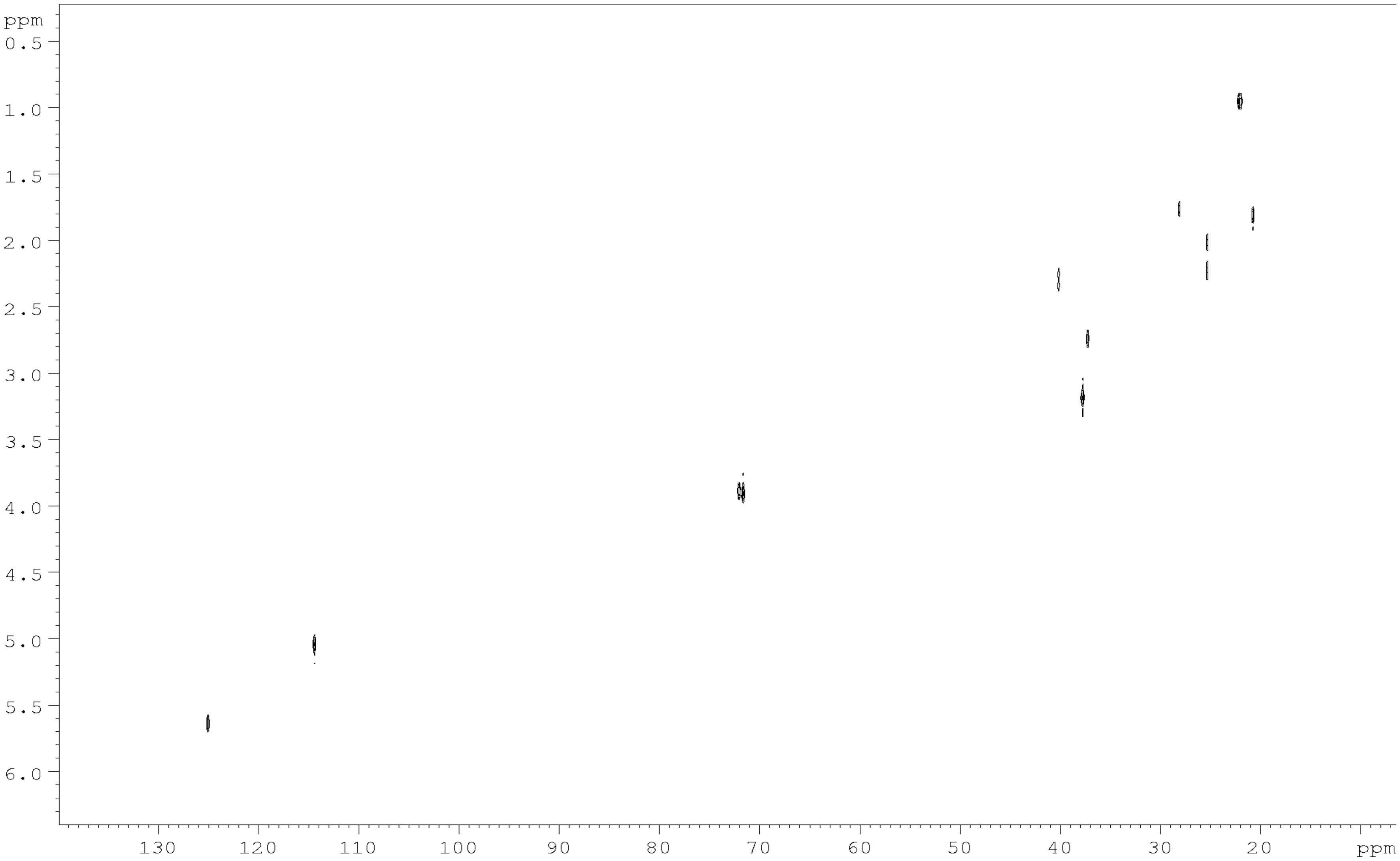

NOESY spectrum of (1R,2R,6S)-6-(3-(isobutylthio)prop-1-en-2-yl)-3-methylcyclohex-3-ene-1,2-diol (**19**)

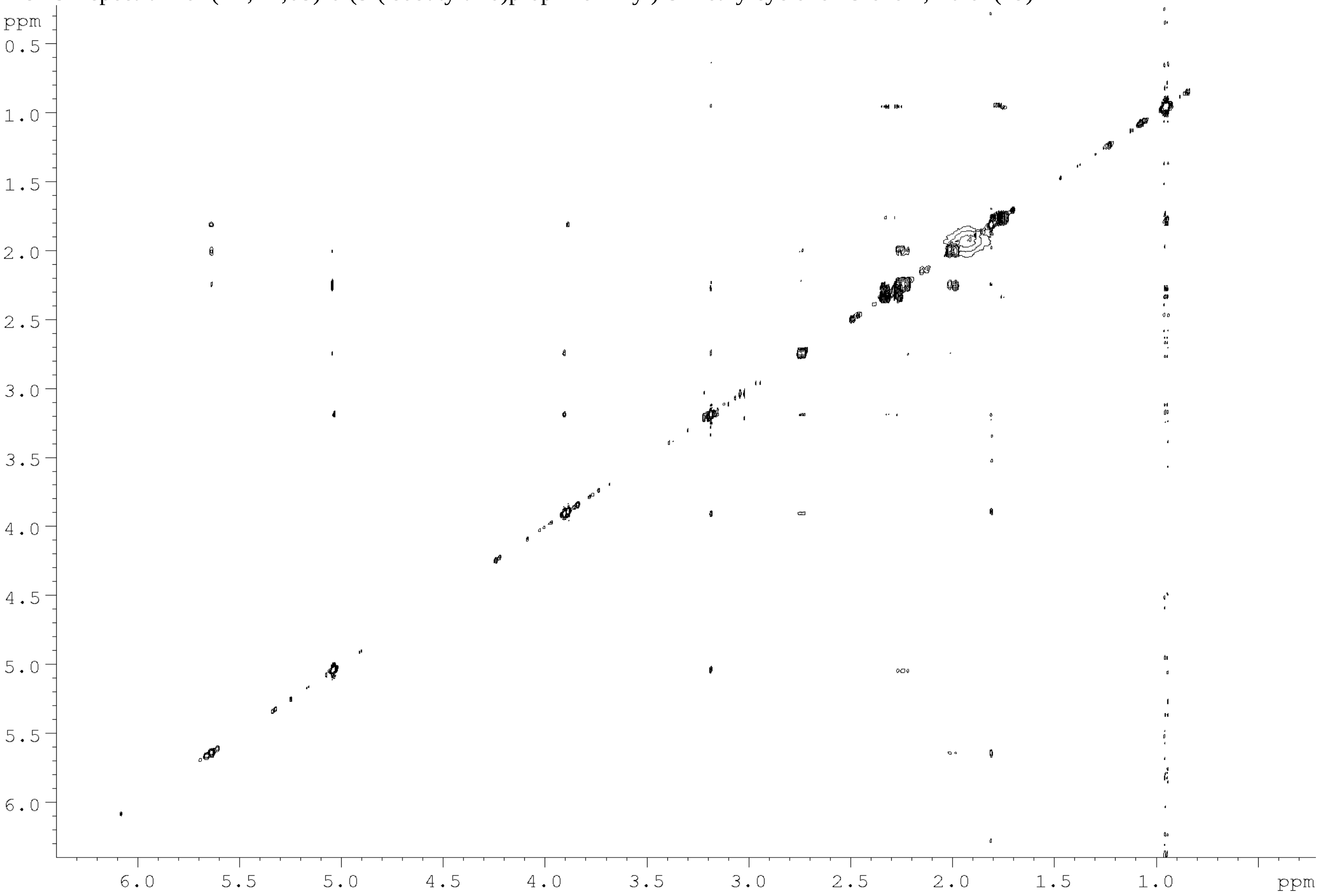

^{1}H NMR spectrum of (1R,2R,6S)-6-(3-(tert-butylthio)prop-1-en-2-yl)-3-methylcyclohex-3-ene-1,2-diol (**20**)

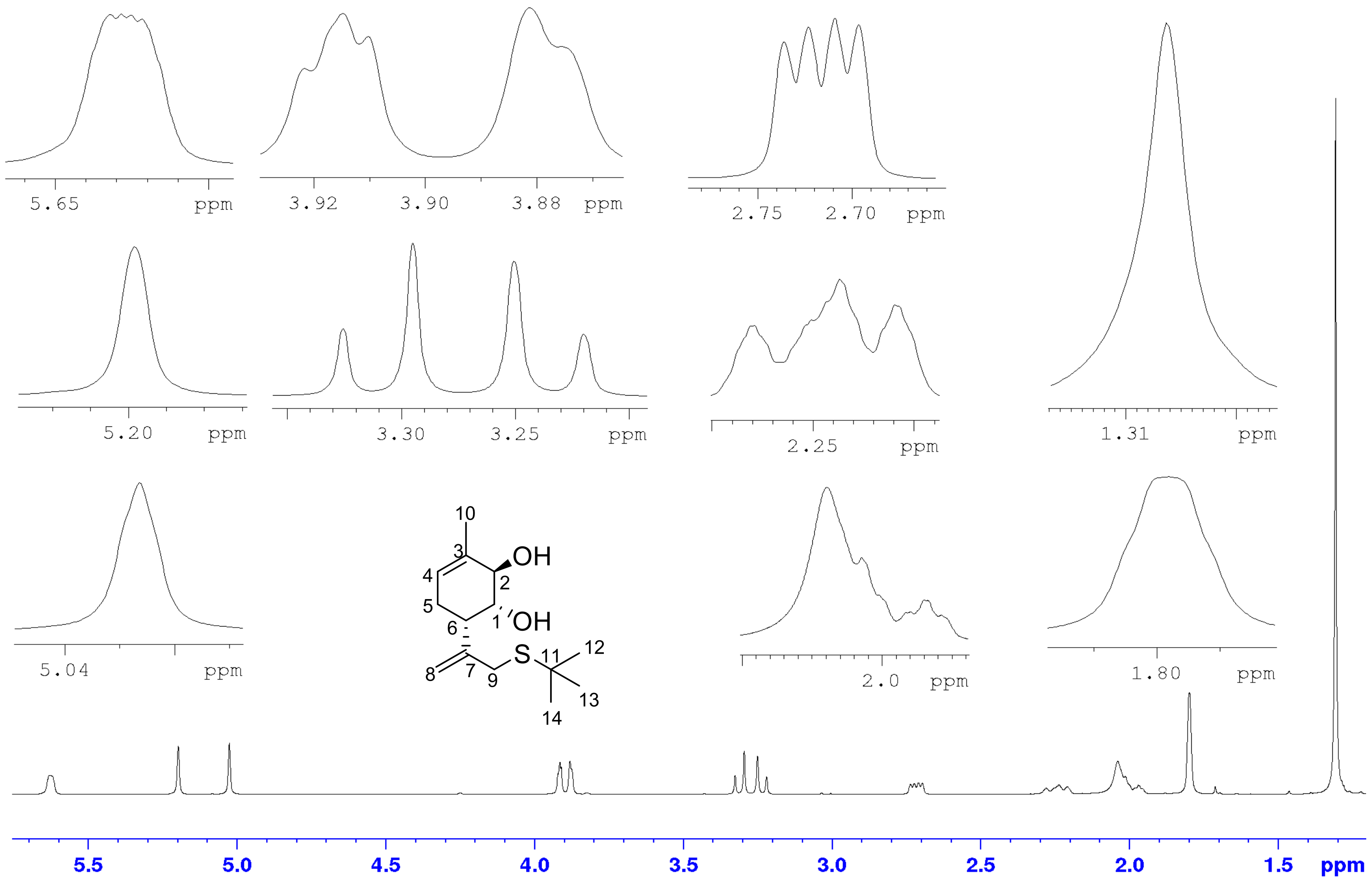

J-modulated ^{13}C NMR spectrum of (1R,2R,6S)-6-(3-(tert-butylthio)prop-1-en-2-yl)-3-methylcyclohex-3-ene-1,2-diol (**20**)

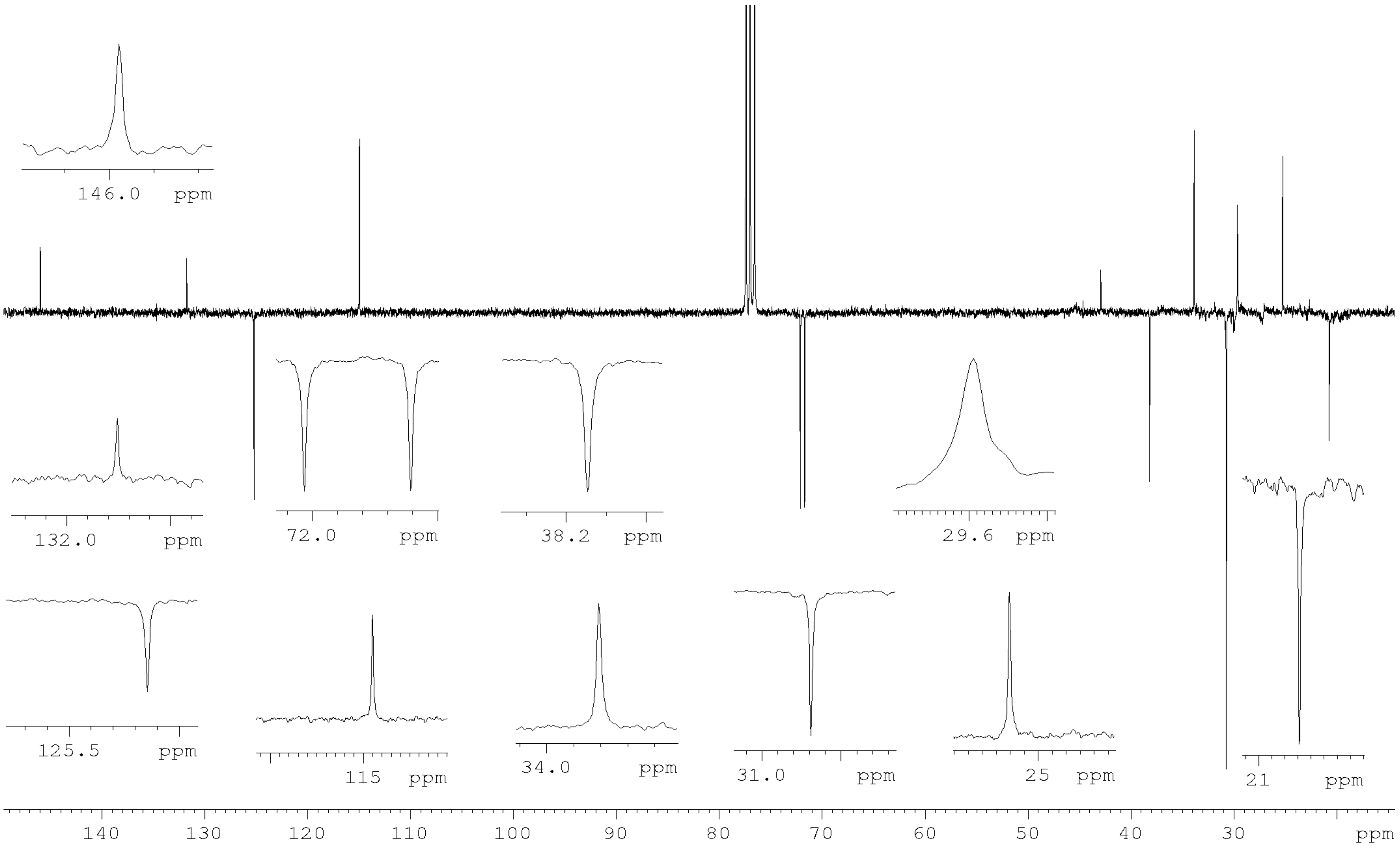

^{1}H NMR spectrum of (1R,2R,6S)-6-(3-(benzylthio)prop-1-en-2-yl)-3-methylcyclohex-3-ene-1,2-diol (**21**)

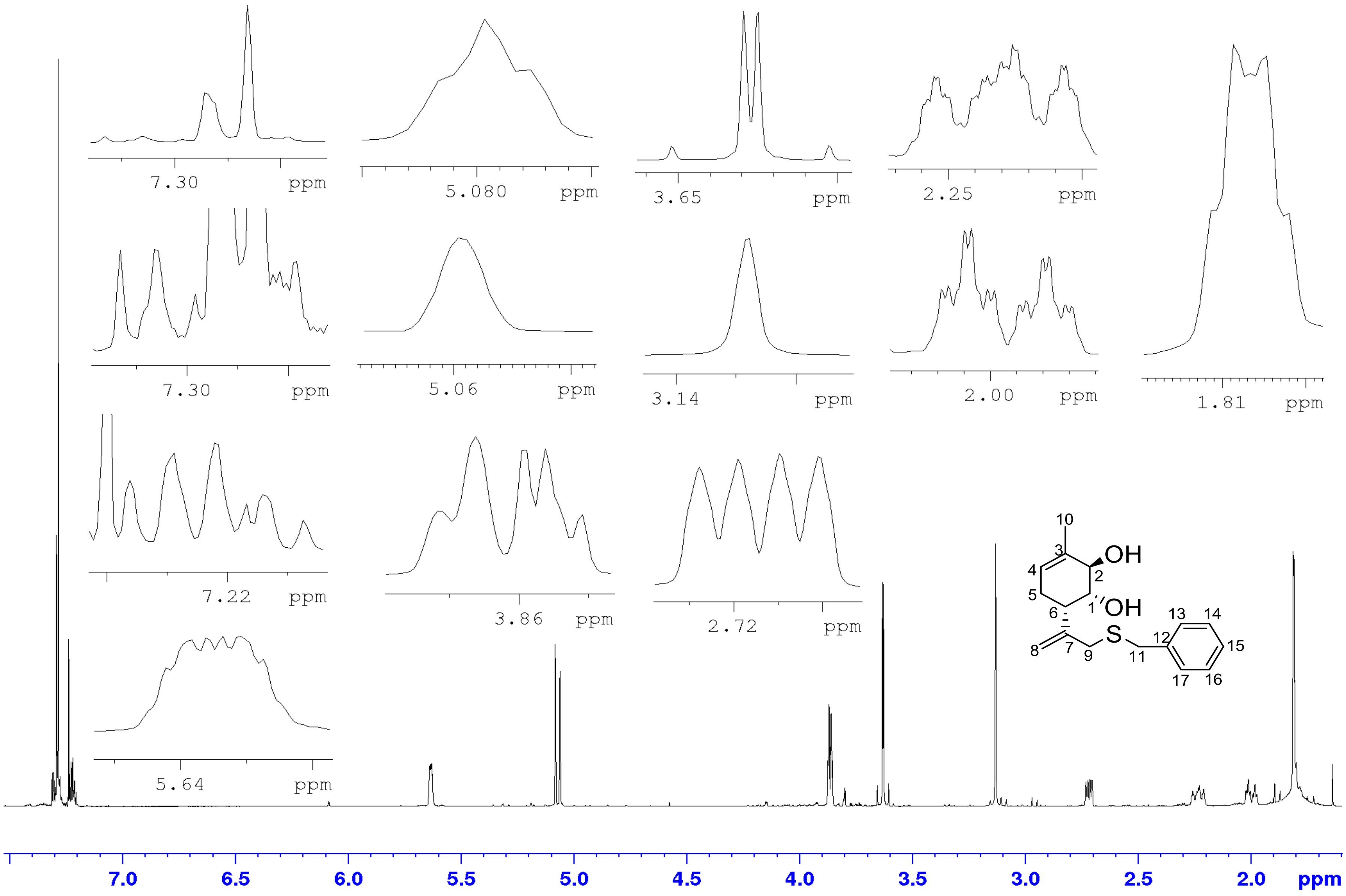

J-modulated ^{13}C NMR spectrum of (1R,2R,6S)-6-(3-(benzylthio)prop-1-en-2-yl)-3-methylcyclohex-3-ene-1,2-diol (**21**)

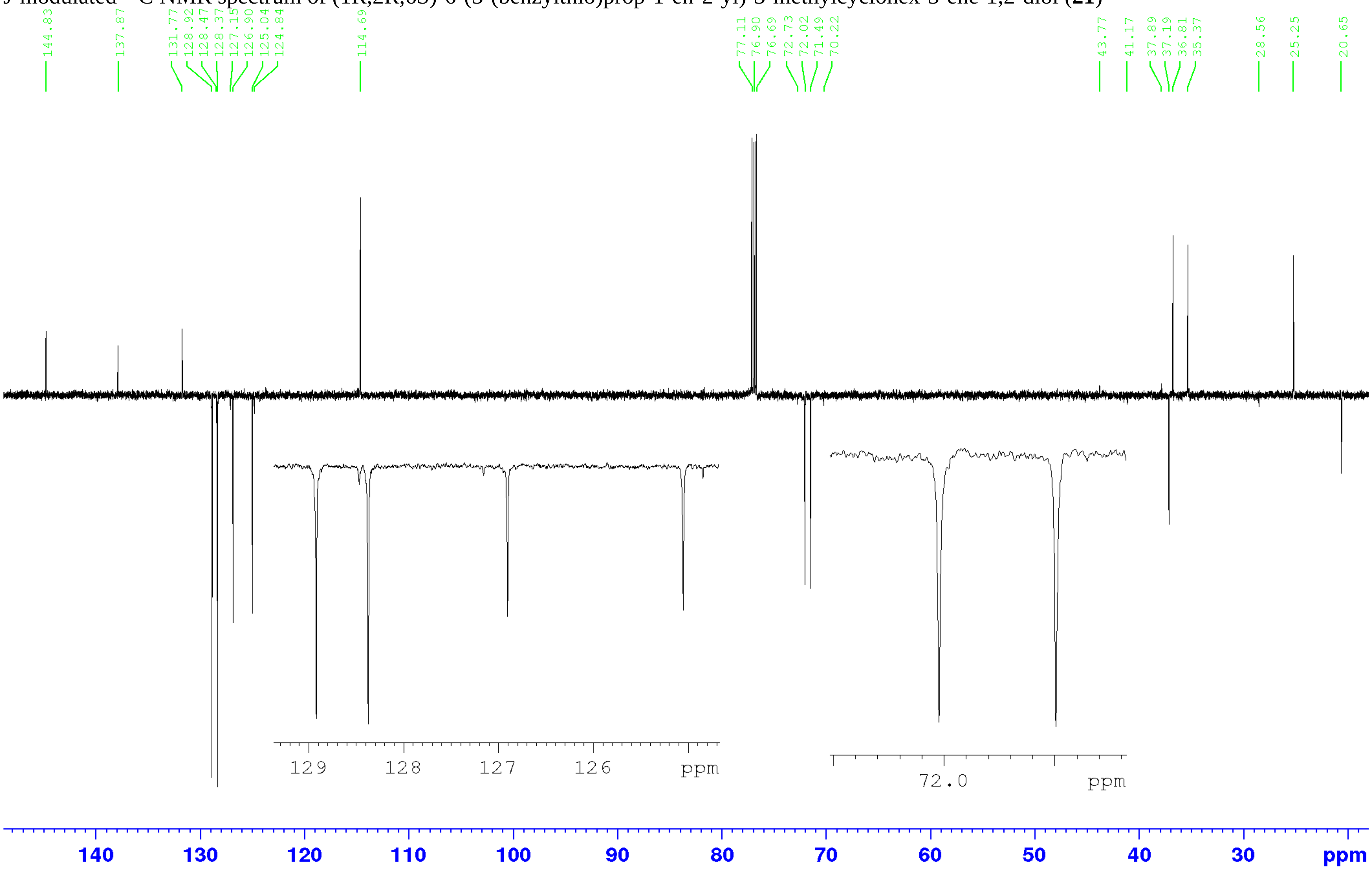

^{1}H-^{1}H 2D homonuclear correlation (COSY) spectrum of (1R,2R,6S)-6-(3-(benzylthio)prop-1-en-2-yl)-3-methylcyclohex-3-ene-1,2-diol (**21**)

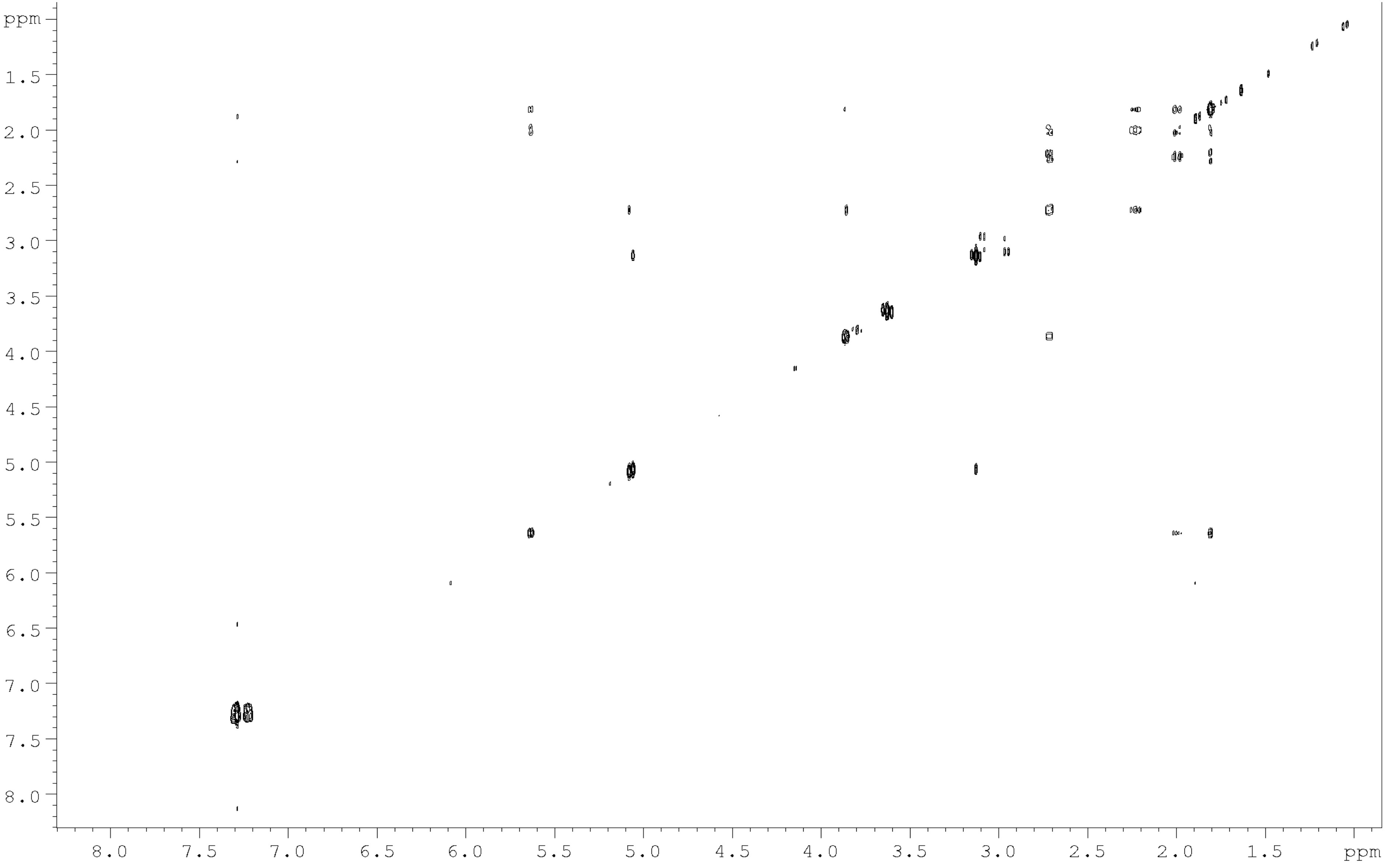

HXCO ^{13}C-^{1}H 2D heteronuclear correlation (C-H COSY) spectrum of (1R,2R,6S)-6-(3-(benzylthio)prop-1-en-2-yl)-3-methylcyclohex-3-ene-1,2-diol (**21**)

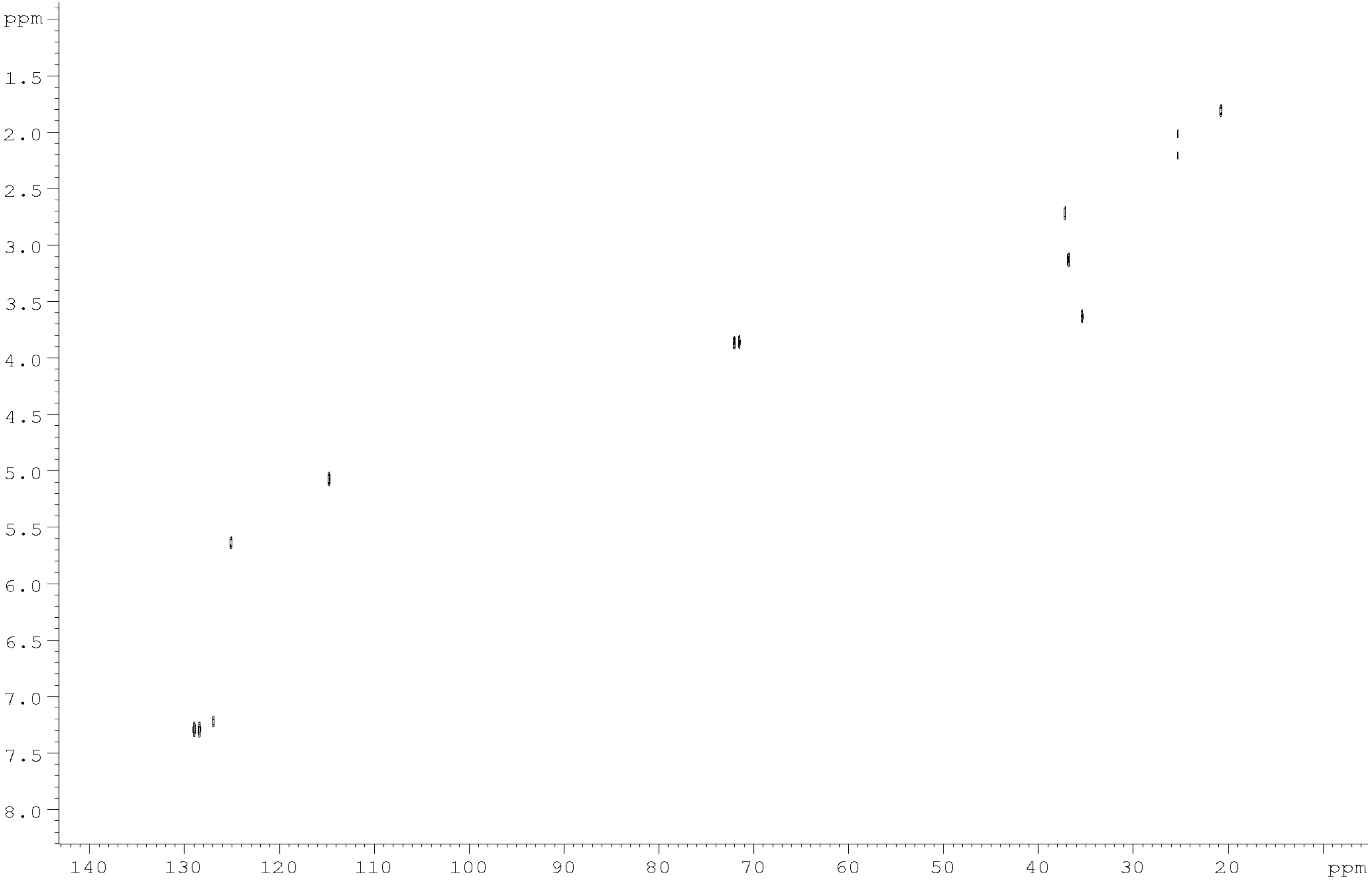

HMBC ^{13}C-^{1}H 2D heteronuclear correlation (C-H COSY) spectrum of (1R,2R,6S)-6-(3-(benzylthio)prop-1-en-2-yl)-3-methylcyclohex-3-ene-1,2-diol (**21**)

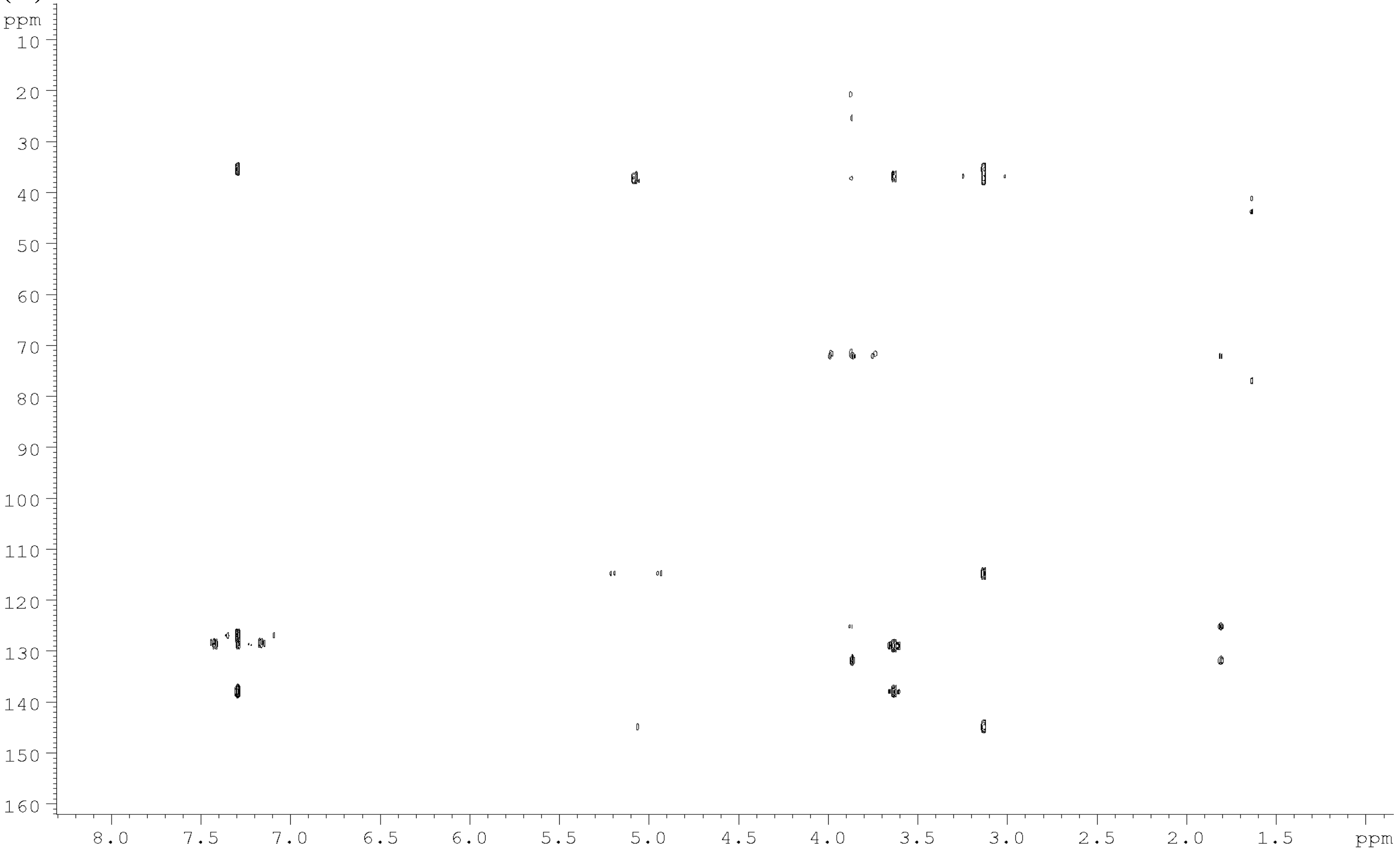

NOESY spectrum of (1R,2R,6S)-6-(3-(benzylthio)prop-1-en-2-yl)-3-methylcyclohex-3-ene-1,2-diol (**21**)

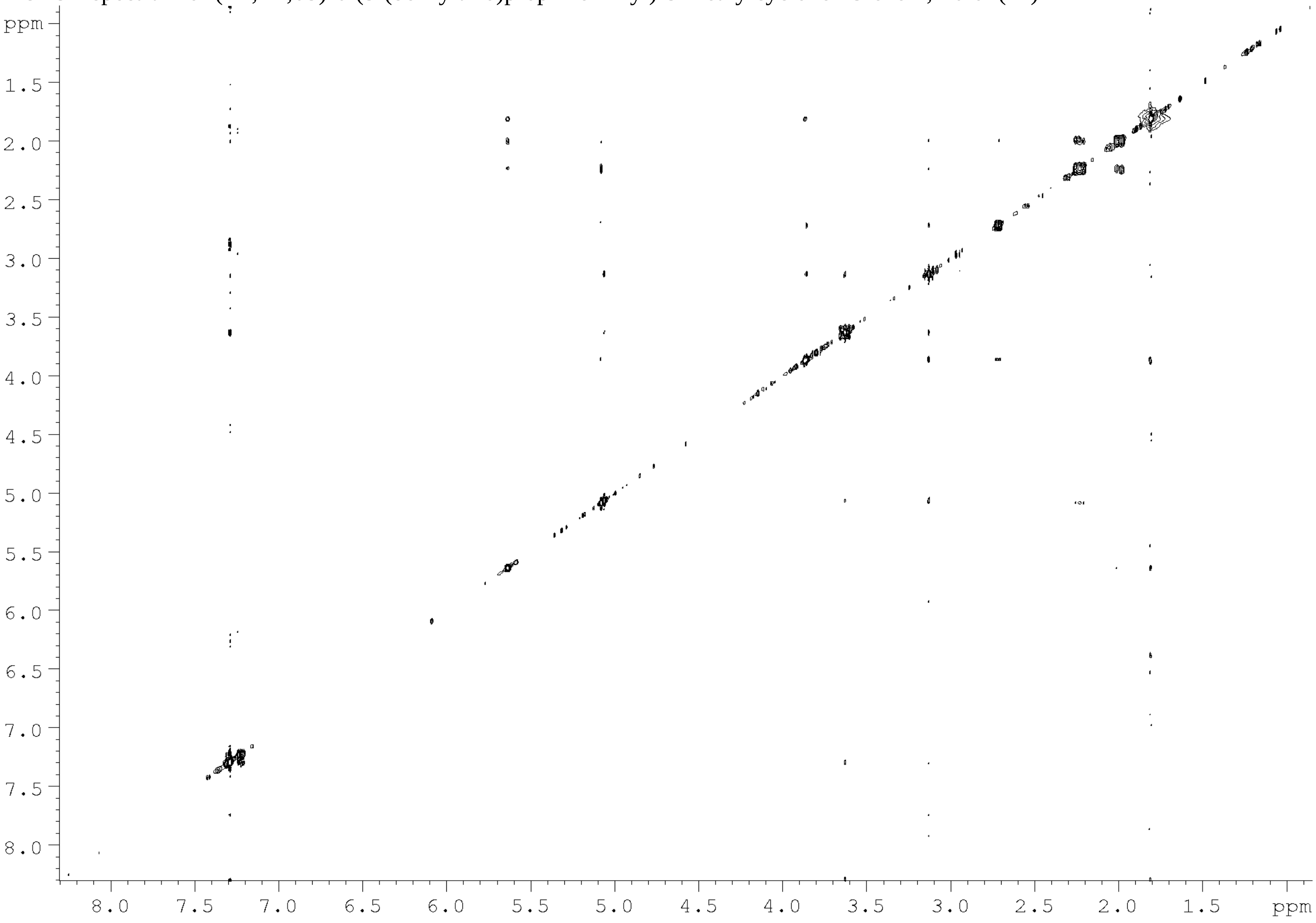

^{1}H NMR spectrum of (1R,2R,6S)-6-(3-(4-chlorophenylthio)prop-1-en-2-yl)-3-methylcyclohex-3-ene-1,2-diol (**22**)

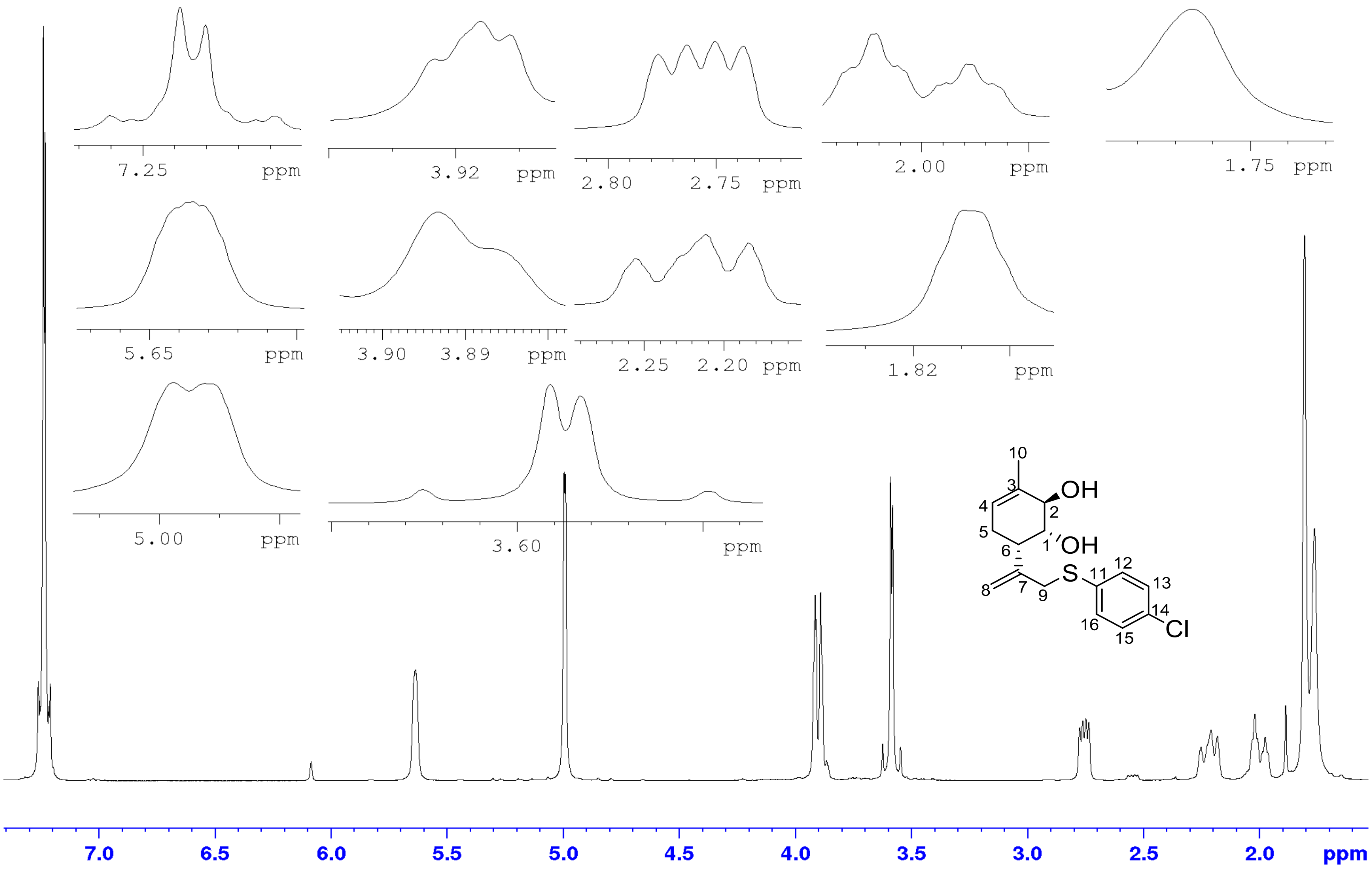

J-modulated ^{13}C NMR spectrum of (1R,2R,6S)-6-(3-(4-chlorophenylthio)prop-1-en-2-yl)-3-methylcyclohex-3-ene-1,2-diol (**22**)

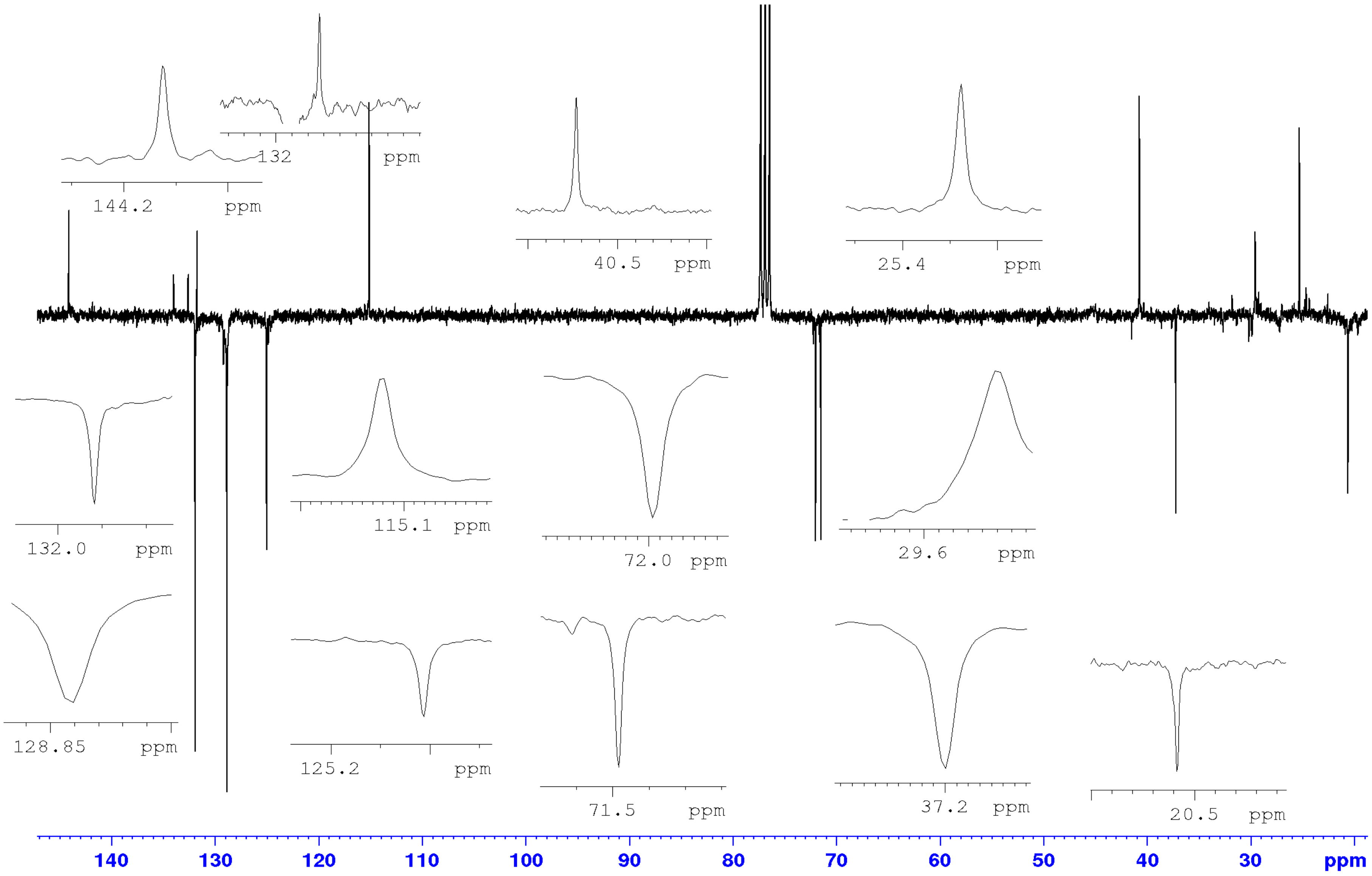

^{1}H NMR spectrum of (1R,2R,6S)-3-methyl-6-(3-(pyridin-2-ylthio)prop-1-en-2-yl)cyclohex-3-ene-1,2-diol (**23**)

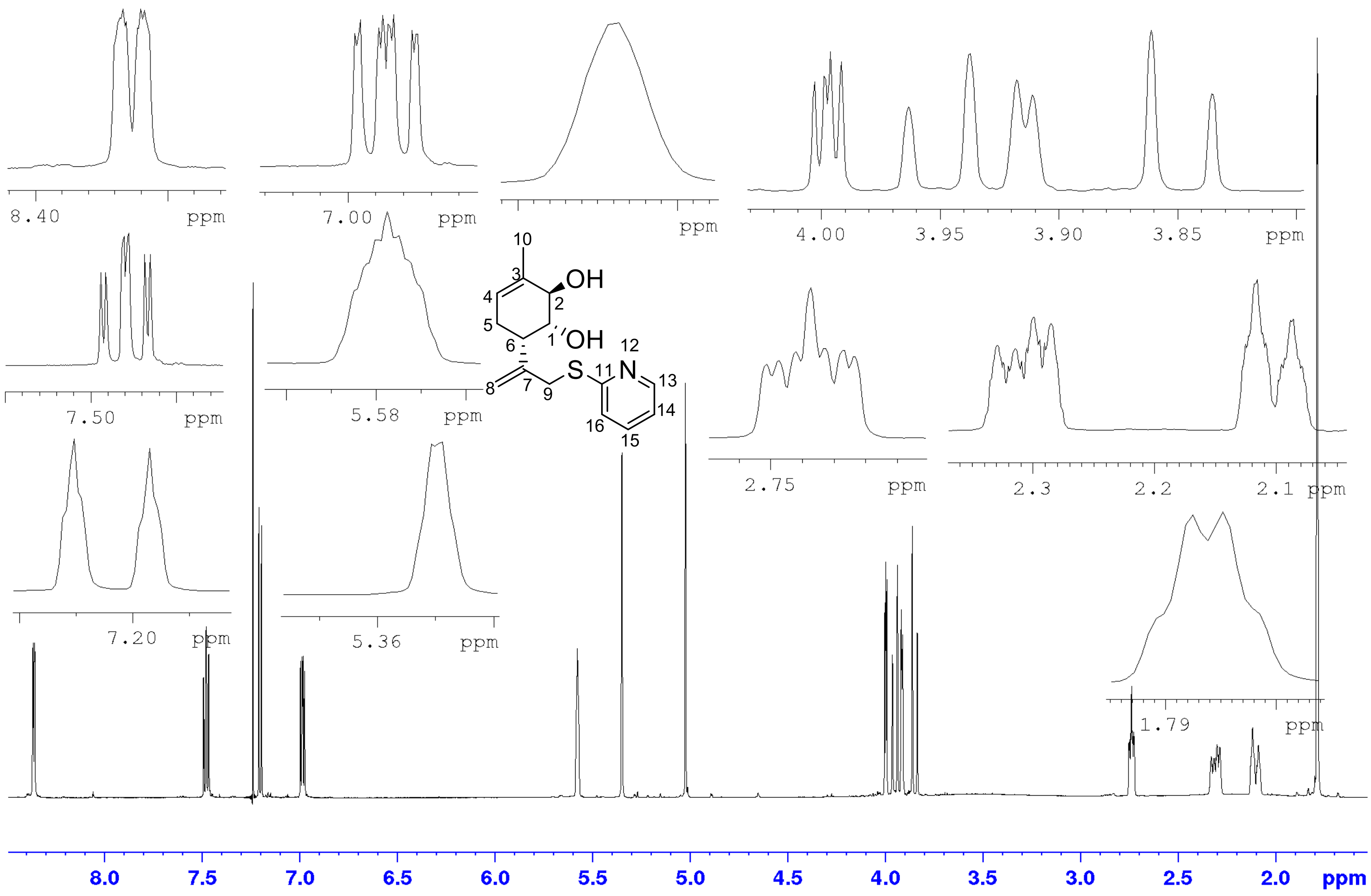

J-modulated ^{13}C NMR spectrum of (1R,2R,6S)-3-methyl-6-(3-(pyridin-2-ylthio)prop-1-en-2-yl)cyclohex-3-ene-1,2-diol (**23**)

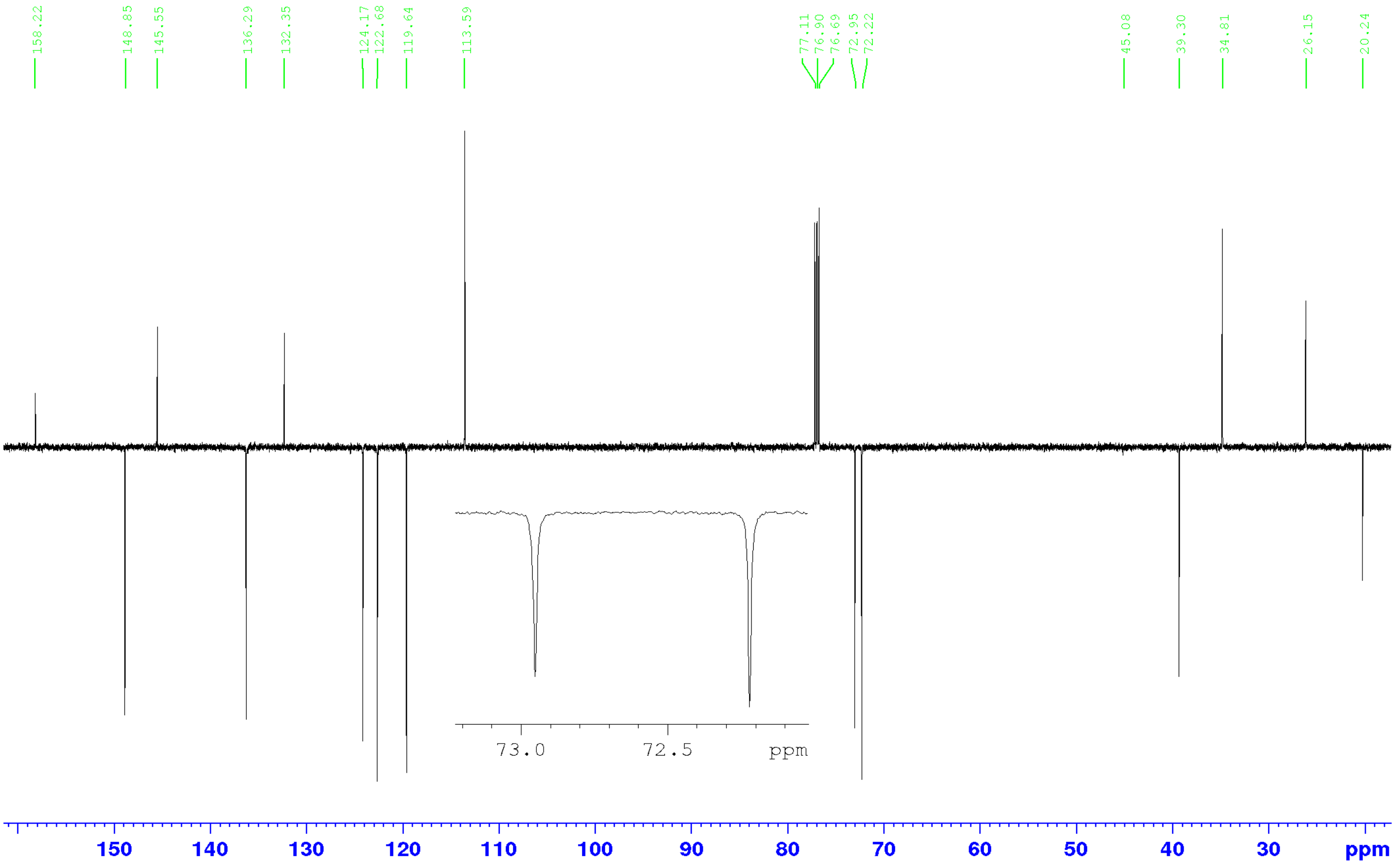

^{1}H-^{1}H 2D homonuclear correlation (COSY) spectrum of (1R,2R,6S)-3-methyl-6-(3-(pyridin-2-ylthio)prop-1-en-2-yl)cyclohex-3-ene-1,2-diol (**23**)

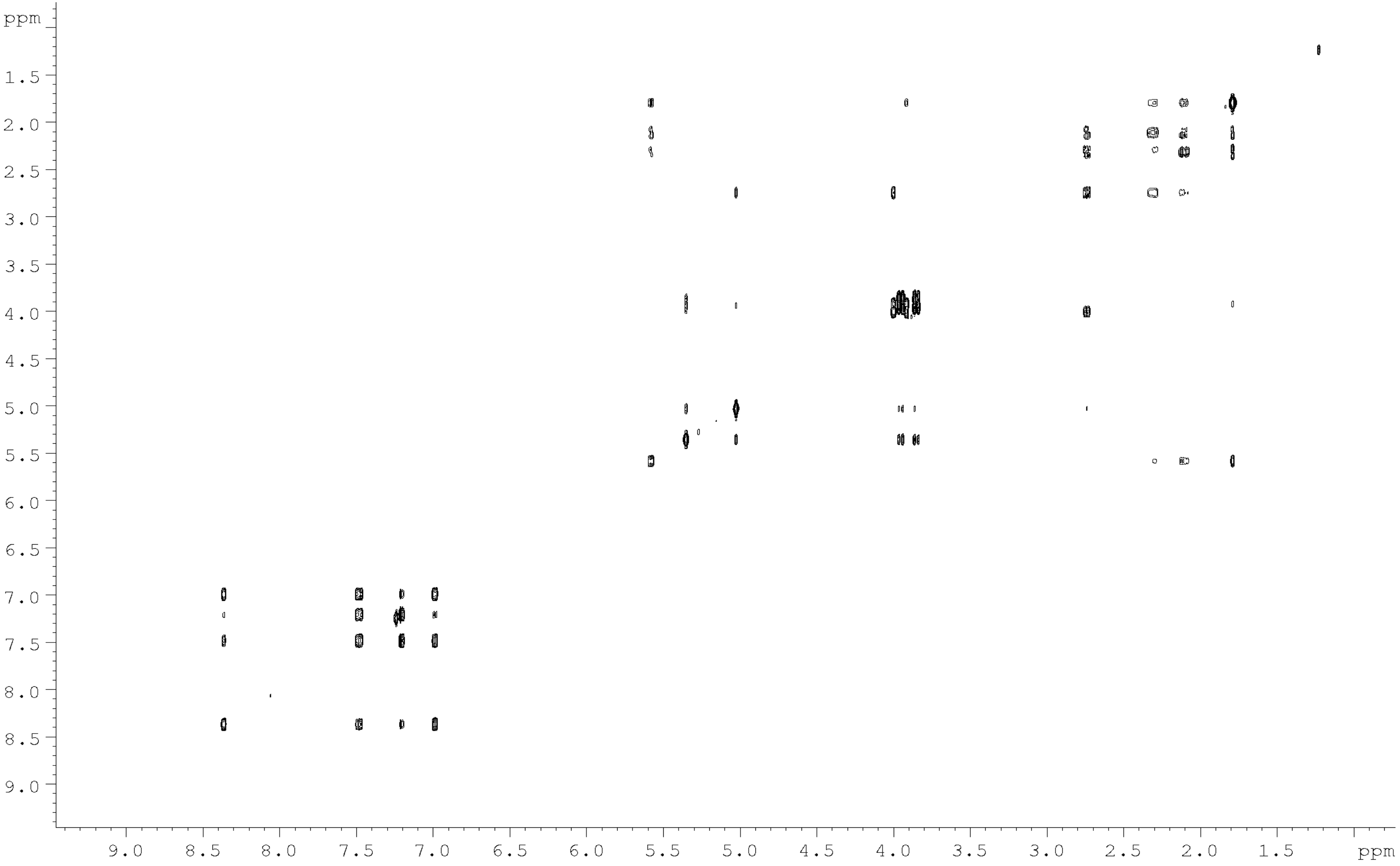

HXCO ^{13}C-^{1}H 2D heteronuclear correlation (C-H COSY) spectrum of (1R,2R,6S)-3-methyl-6-(3-(pyridin-2-ylthio)prop-1-en-2-yl)cyclohex-3-ene-1,2-diol (**23**)

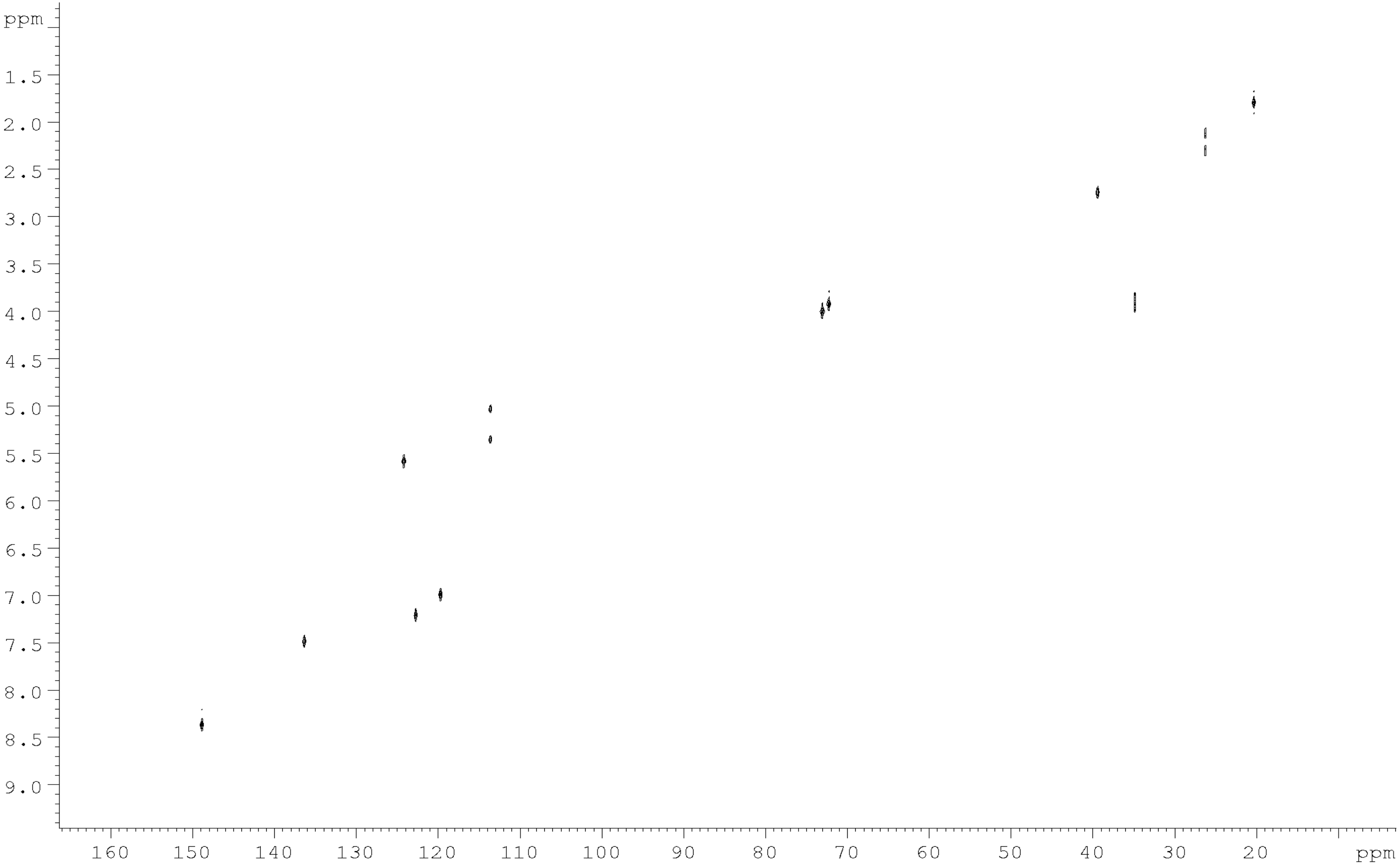

HMBC ^{13}C-^{1}H 2D heteronuclear correlation (C-H COSY) spectrum of (1R,2R,6S)-3-methyl-6-(3-(pyridin-2-ylthio)prop-1-en-2-yl)cyclohex-3-ene-1,2-diol (**23**)

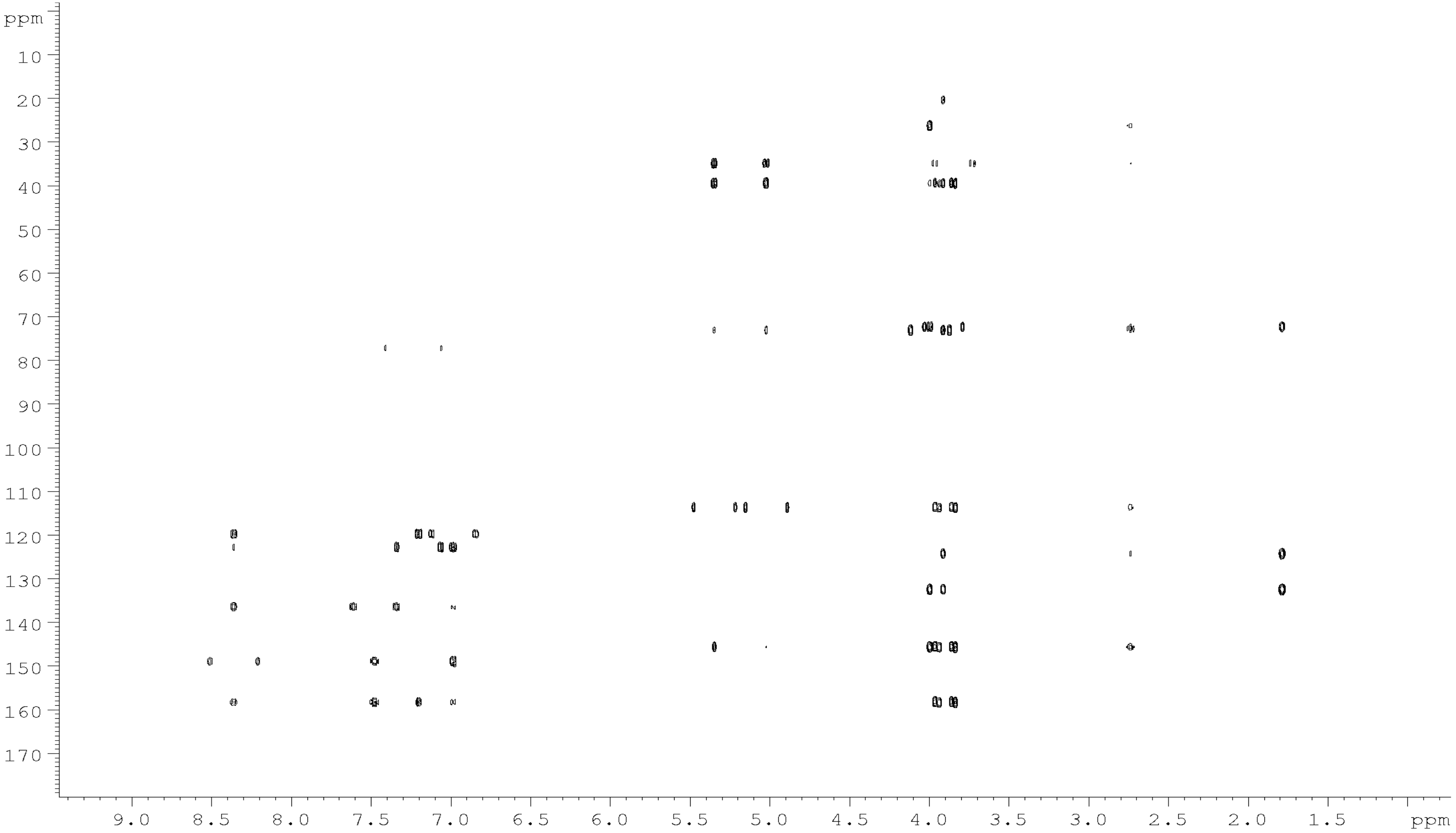

NOESY spectrum of (1R,2R,6S)-3-methyl-6-(3-(pyridin-2-ylthio)prop-1-en-2-yl)cyclohex-3-ene-1,2-diol (**23**)

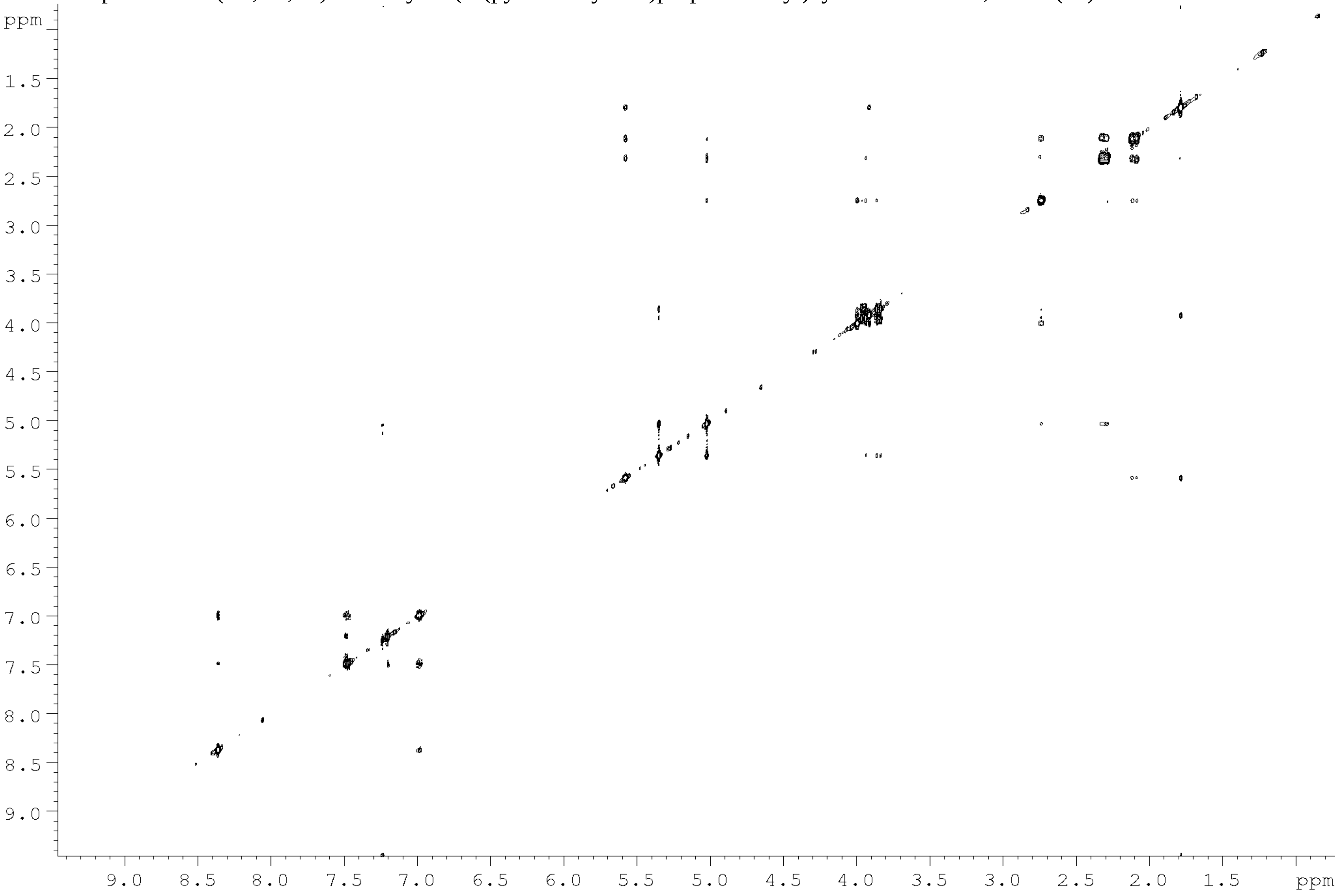

^{1}H NMR spectrum of (1R,2R,6S)-3-methyl-6-(3-(pyrimidin-2-ylthio)prop-1-en-2-yl)cyclohex-3-ene-1,2-diol (**24**)

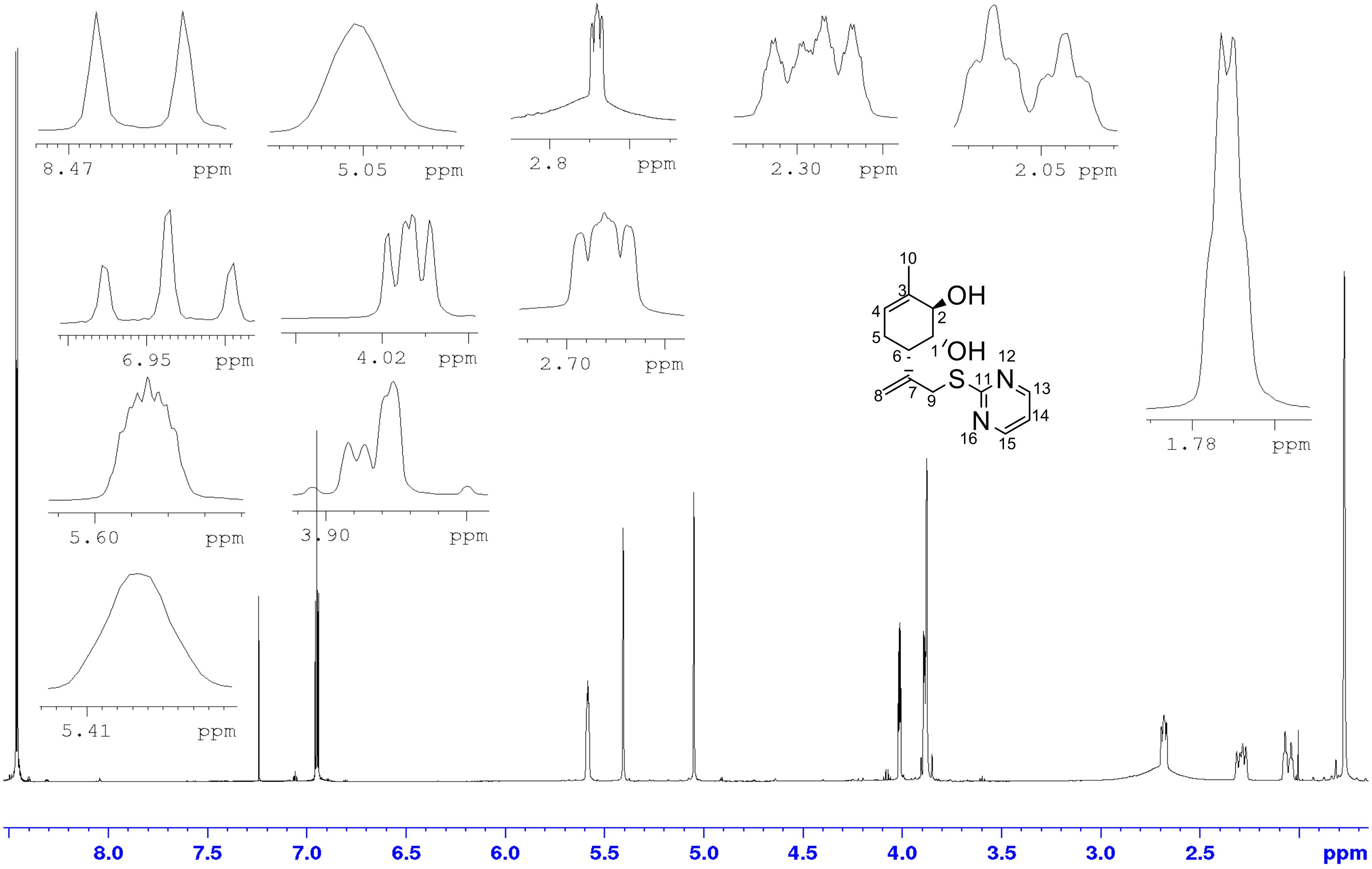

J-modulated ^{13}C NMR spectrum of (1R,2R,6S)-3-methyl-6-(3-(pyrimidin-2-ylthio)prop-1-en-2-yl)cyclohex-3-ene-1,2-diol (**24**)

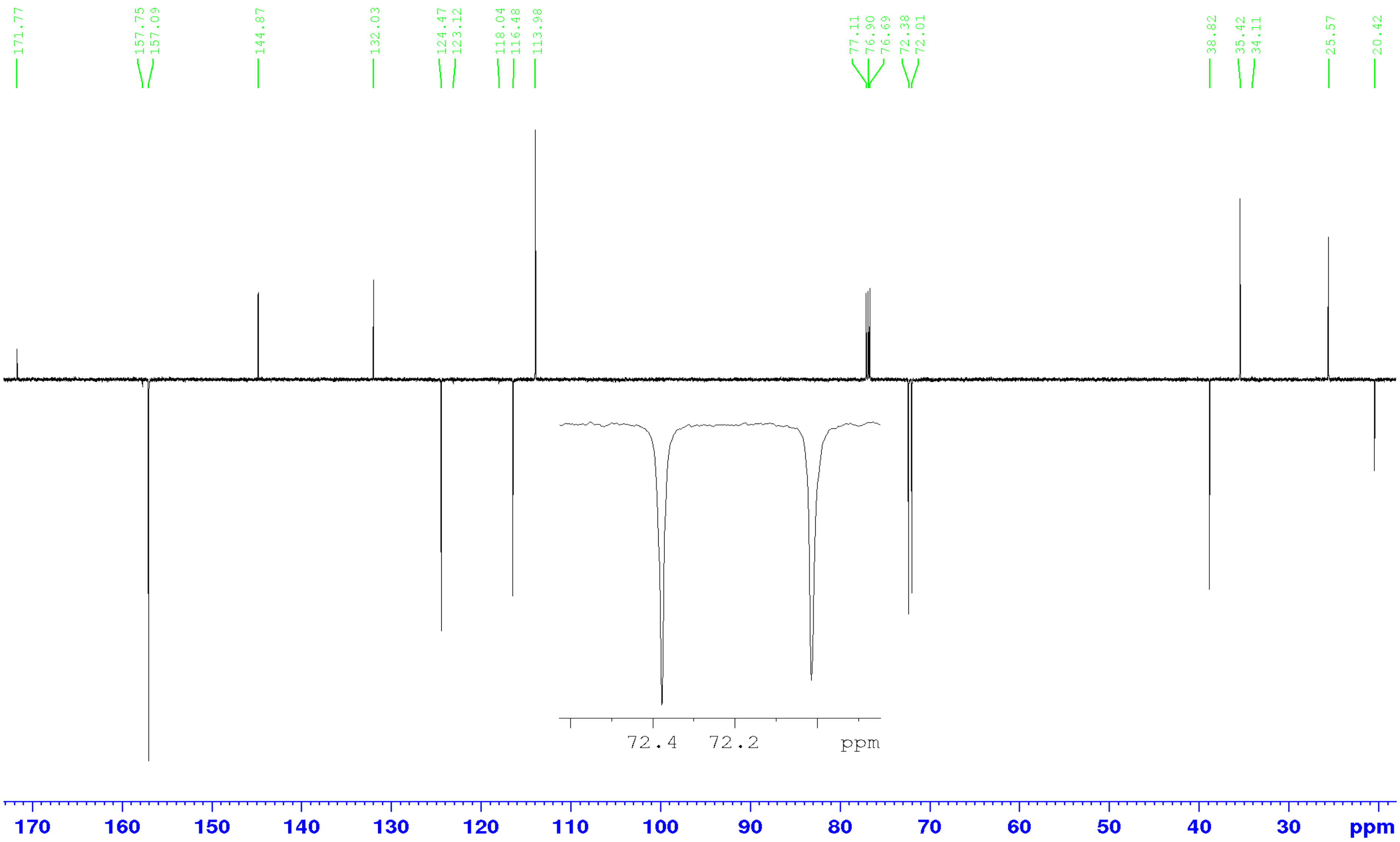

^{1}H-^{1}H 2D homonuclear correlation (COSY) spectrum of (1R,2R,6S)-3-methyl-6-(3-(pyrimidin-2-ylthio)prop-1-en-2-yl)cyclohex-3-ene-1,2-diol (**24**)

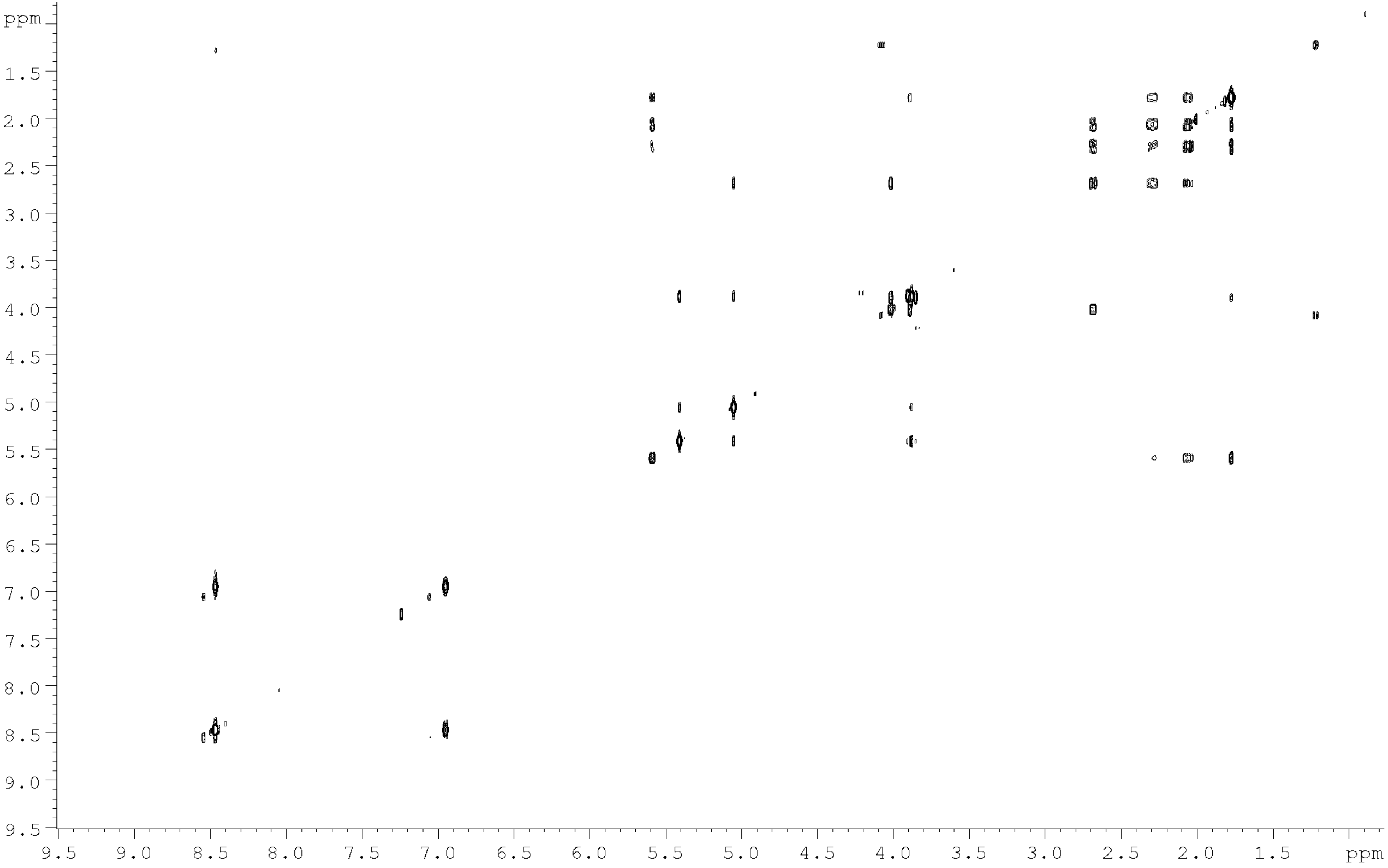

HXCO ^{13}C-^{1}H 2D heteronuclear correlation (C-H COSY) spectrum of (1R,2R,6S)-3-methyl-6-(3-(pyrimidin-2-ylthio)prop-1-en-2-yl)cyclohex-3-ene-1,2-diol (**24**)

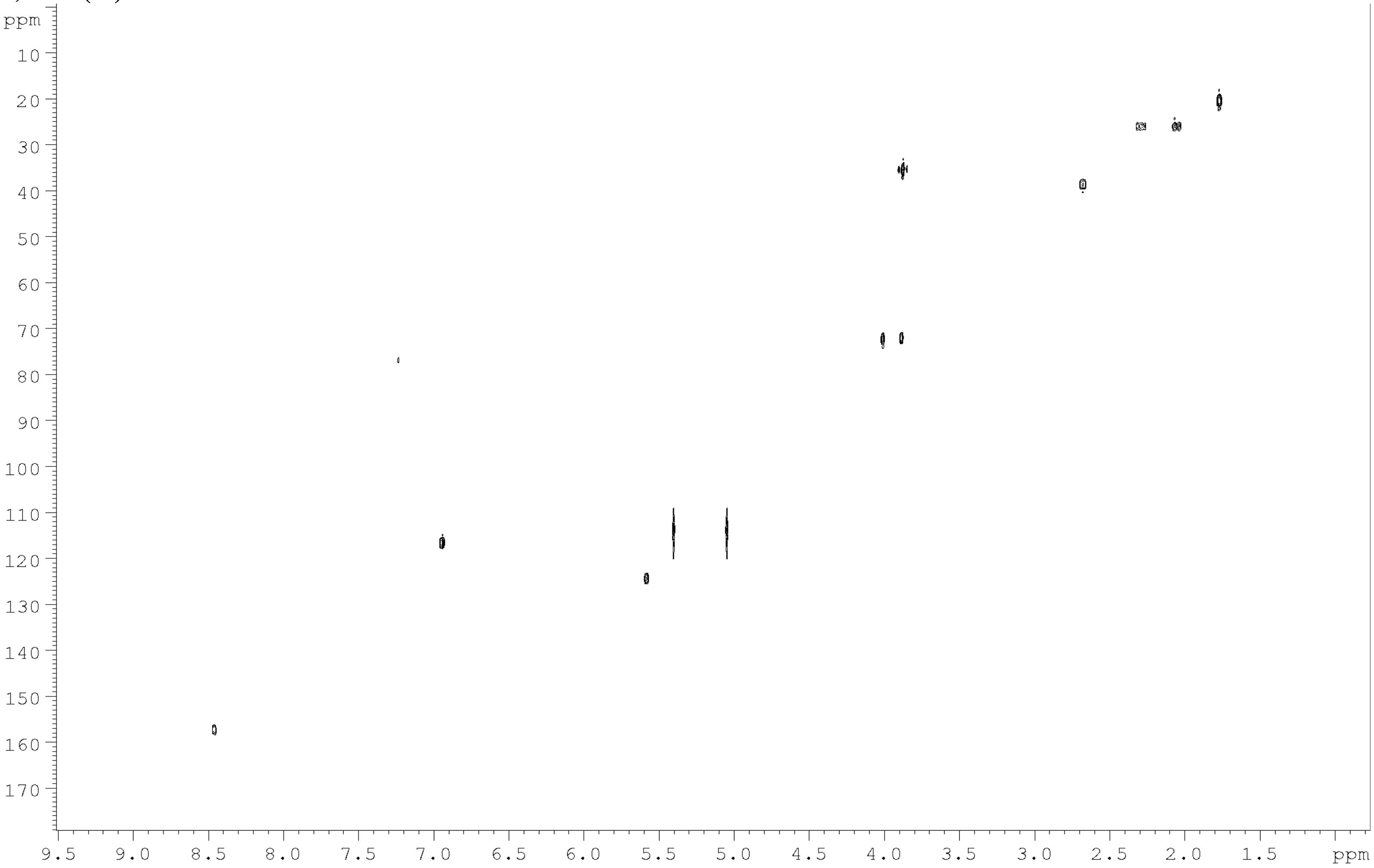

HMBC ^{13}C-^{1}H 2D heteronuclear correlation (C-H COSY) spectrum of (1R,2R,6S)-3-methyl-6-(3-(pyrimidin-2-ylthio)prop-1-en-2-yl)cyclohex-3-ene-1,2-diol (**24**)

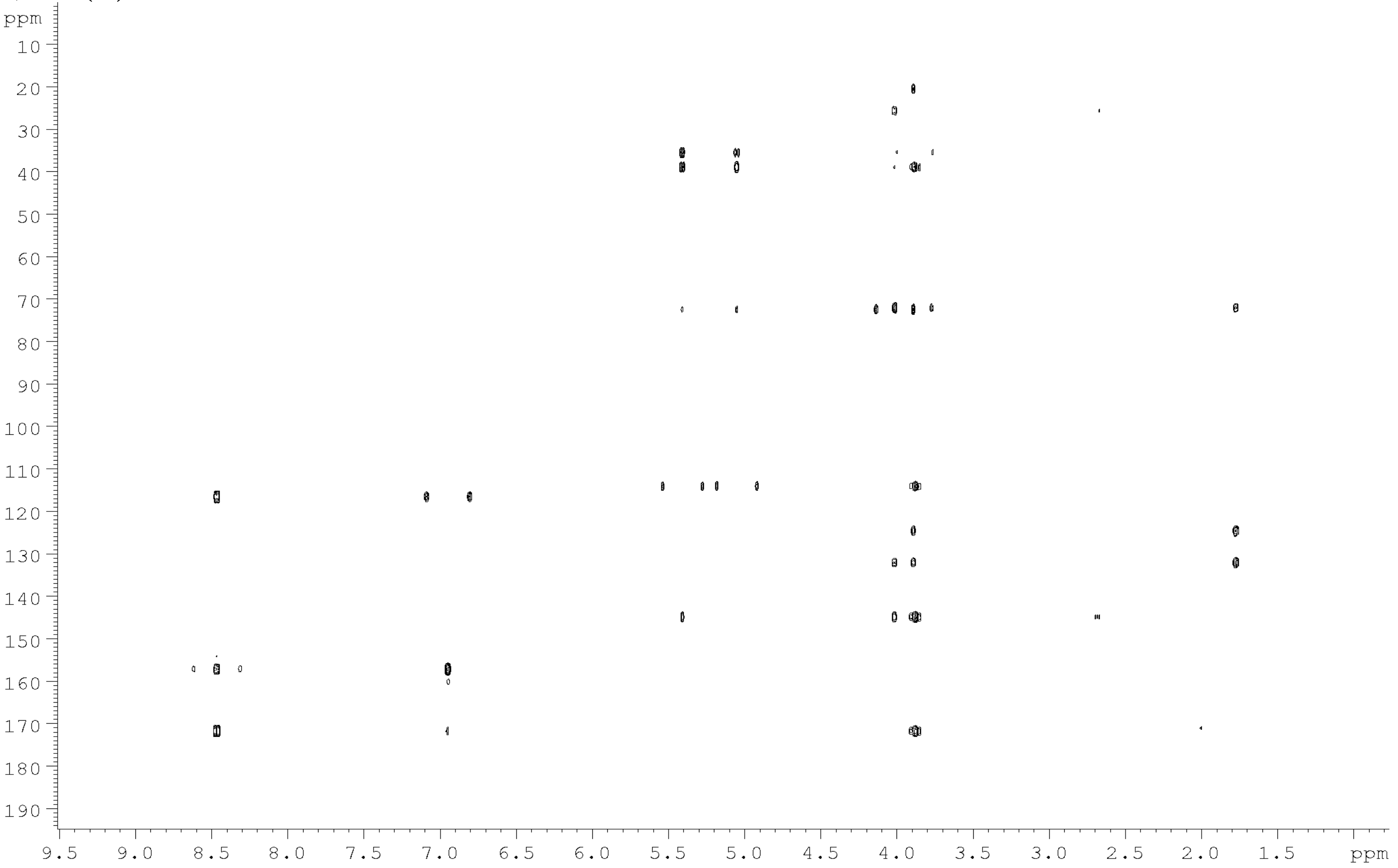

NOESY spectrum of (1R,2R,6S)-3-methyl-6-(3-(pyrimidin-2-ylthio)prop-1-en-2-yl)cyclohex-3-ene-1,2-diol (**24**)

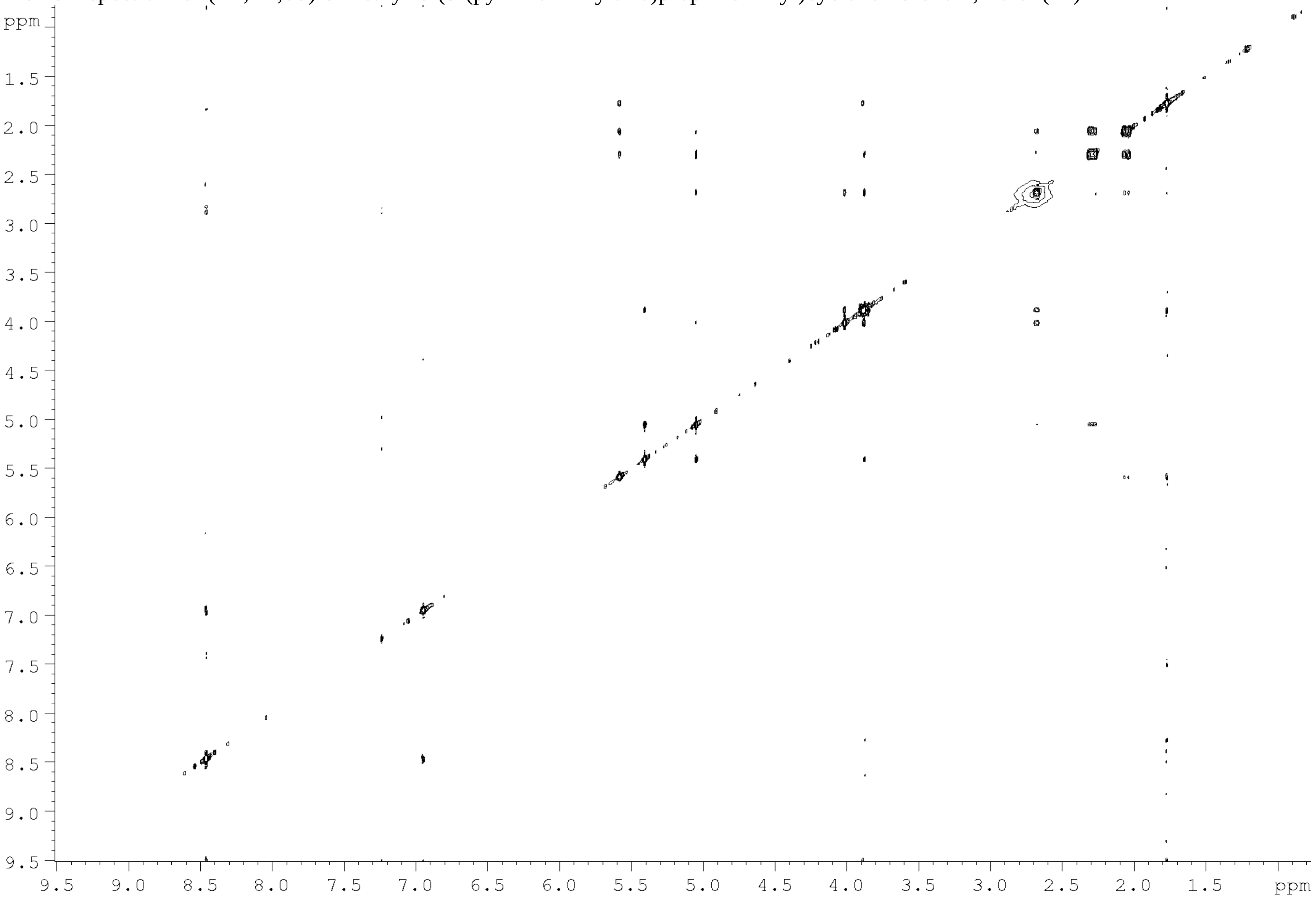

^{1}H NMR spectrum of (1R,2R,6S)-6-(3-(benzo[d]oxazol-2-ylthio)prop-1-en-2-yl)-3-methylcyclohex-3-ene-1,2-diol (**25**)

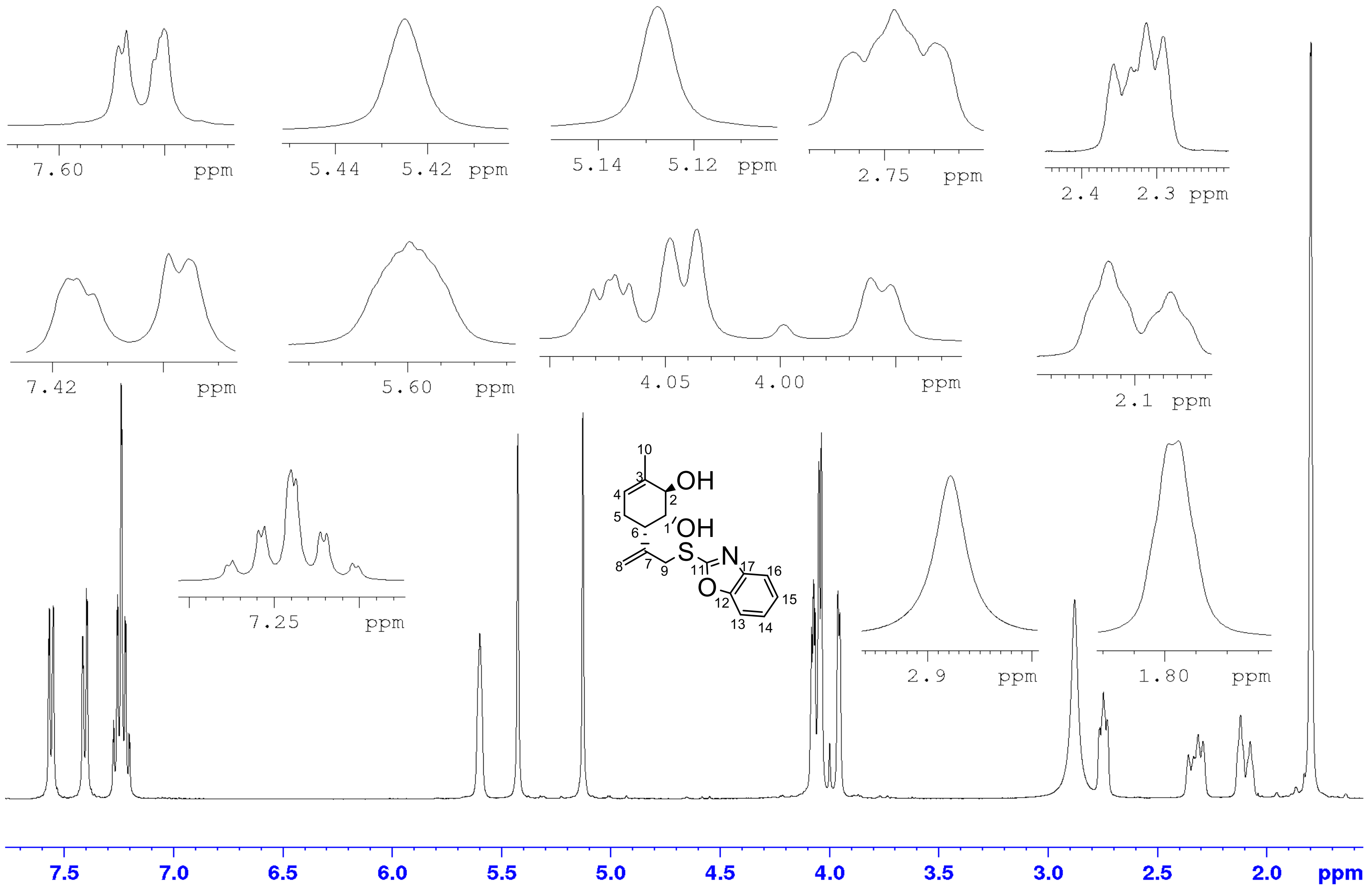

J-modulated ^{13}C NMR spectrum of (1R,2R,6S)-6-(3-(benzo[d]oxazol-2-ylthio)prop-1-en-2-yl)-3-methylcyclohex-3-ene-1,2-diol (**25**)

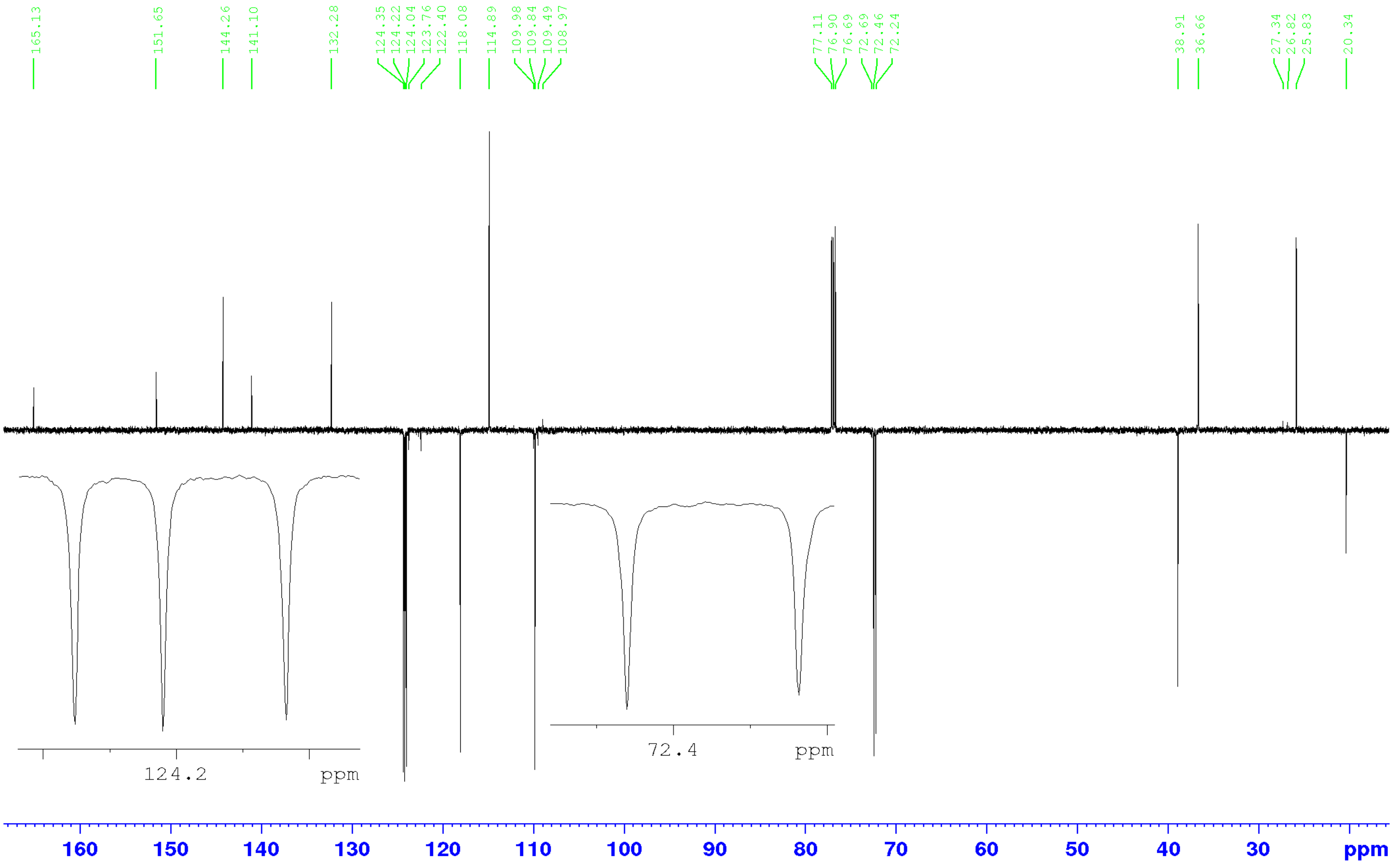

^{1}H-^{1}H 2D homonuclear correlation (COSY) spectrum of (1R,2R,6S)-6-(3-(benzo[d]oxazol-2-ylthio)prop-1-en-2-yl)-3-methylcyclohex-3-ene-1,2-diol **(25)**

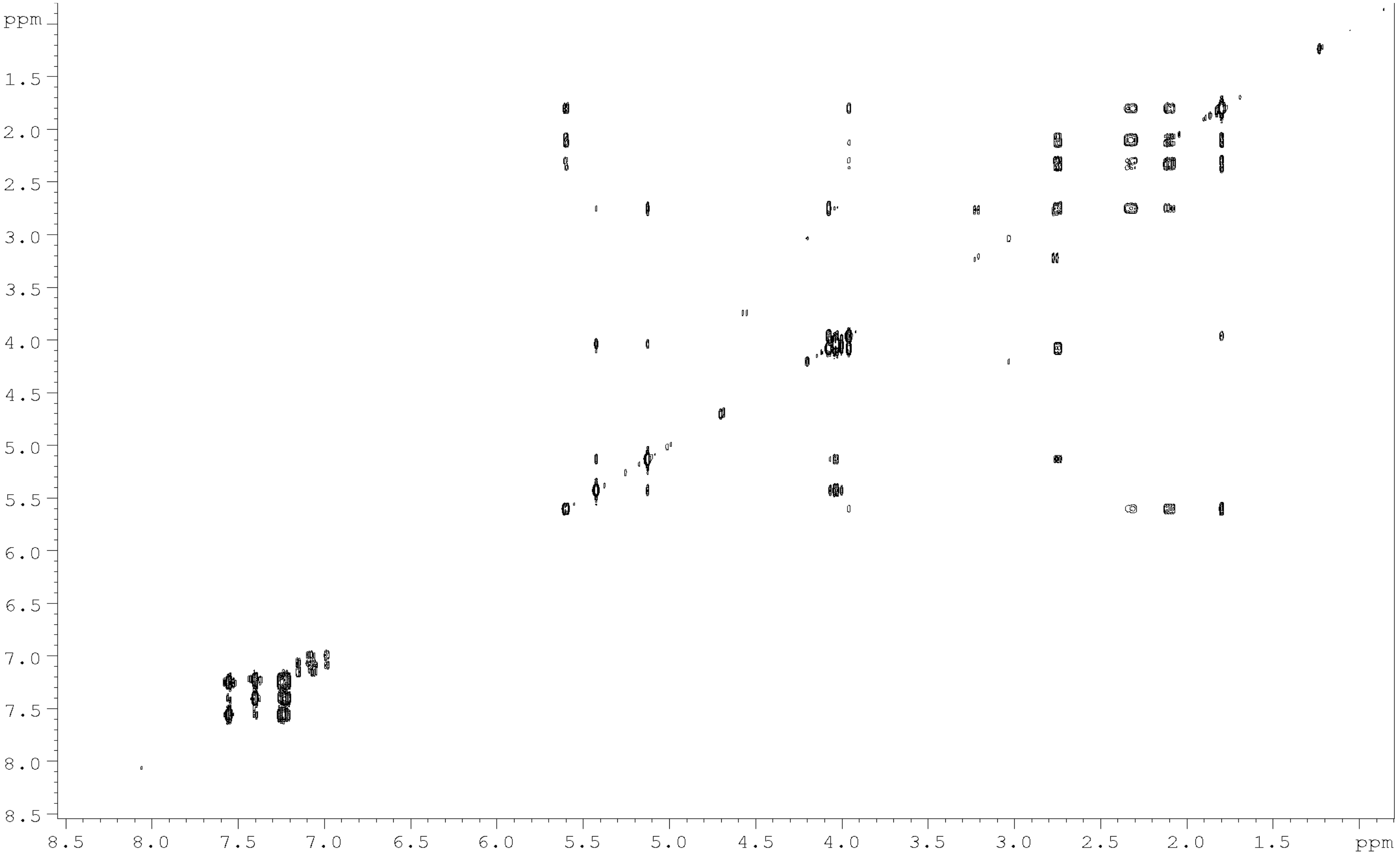

HXCO ^{13}C-^{1}H 2D heteronuclear correlation (C-H COSY) spectrum of (1R,2R,6S)-6-(3-(benzo[d]oxazol-2-ylthio)prop-1-en-2-yl)-3-methylcyclohex-3-ene-1,2-diol (**25**)

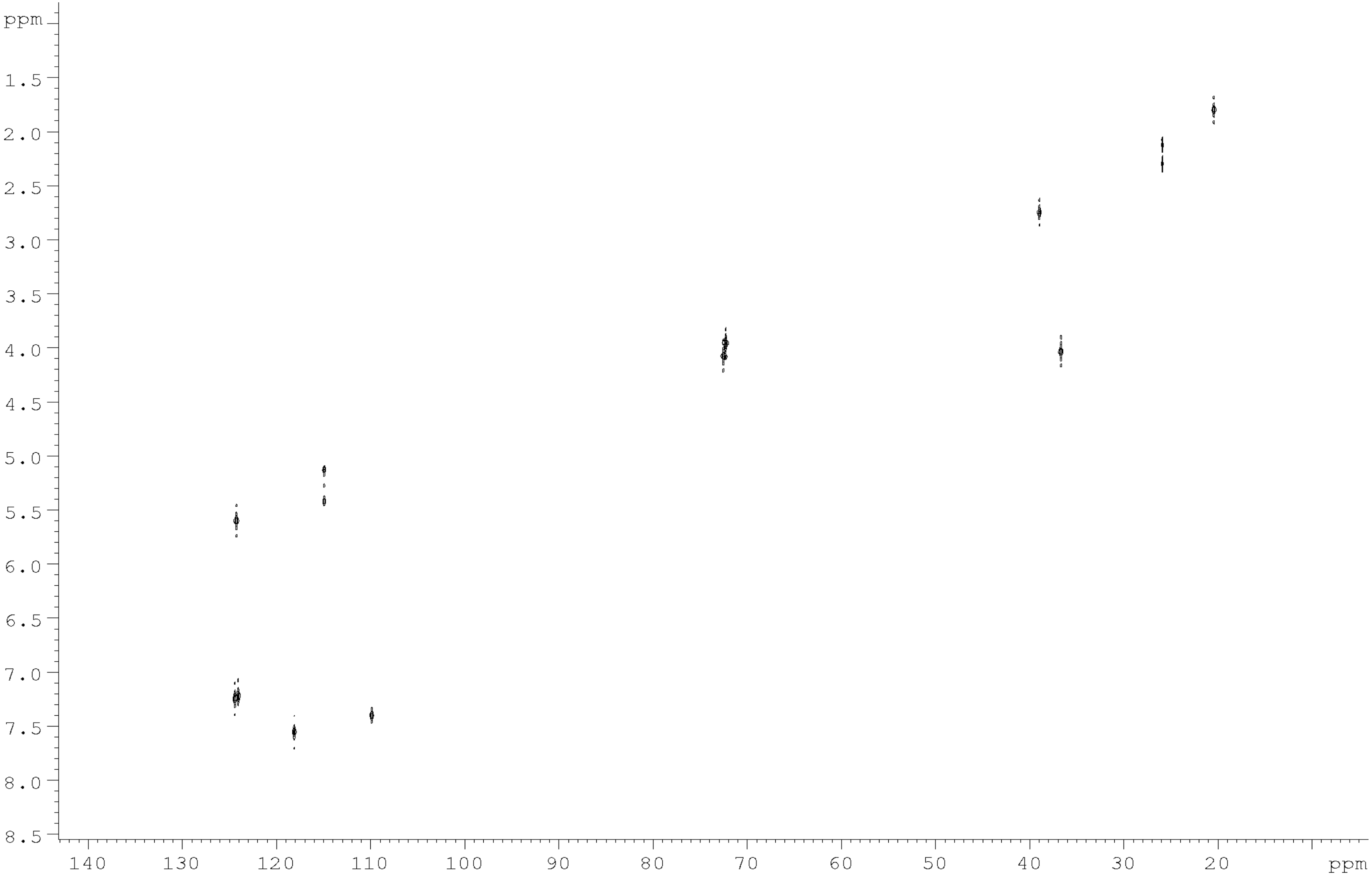

HMBC ^{13}C-^{1}H 2D heteronuclear correlation (C-H COSY) spectrum of (1R,2R,6S)-6-(3-(benzo[d]oxazol-2-ylthio)prop-1-en-2-yl)-3-methylcyclohex-3-ene-1,2-diol (**25**)

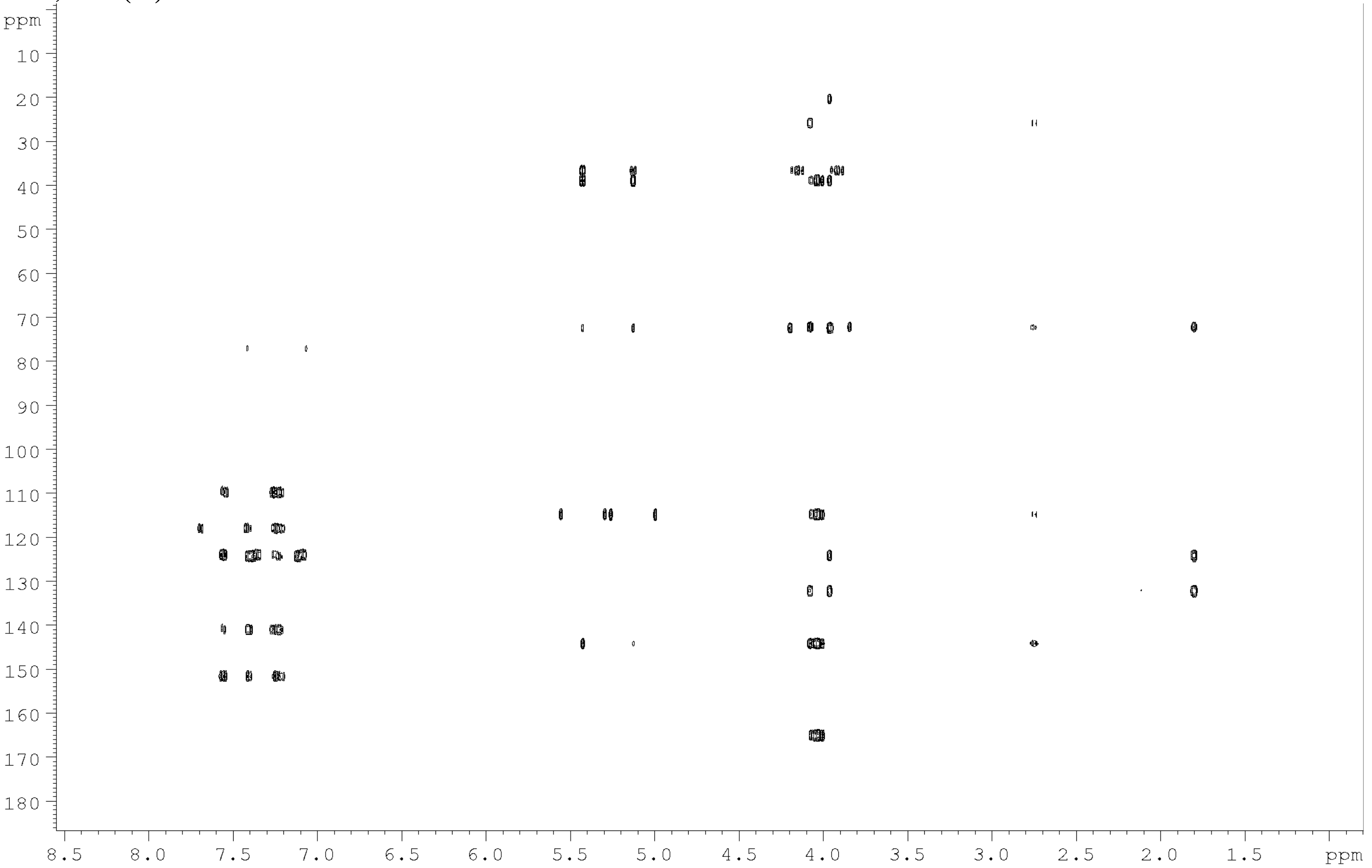

NOESY spectrum of (1R,2R,6S)-6-(3-(benzo[d]oxazol-2-ylthio)prop-1-en-2-yl)-3-methylcyclohex-3-ene-1,2-diol (**25**)

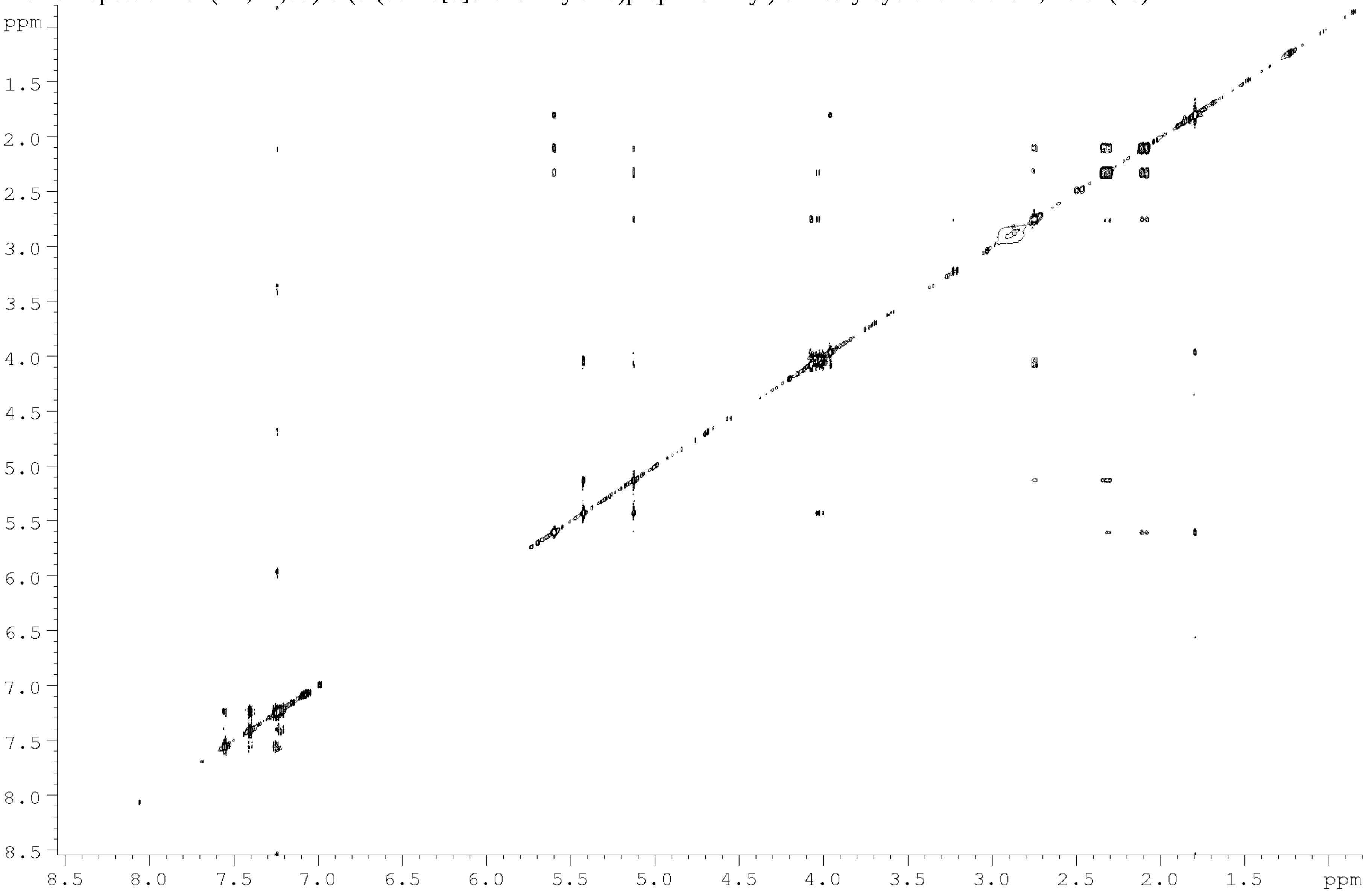

^{1}H NMR spectrum of (1R,2R,6S)-3-methyl-6-(3-(phenylamino)prop-1-en-2-yl)cyclohex-3-ene-1,2-diol (**26**)

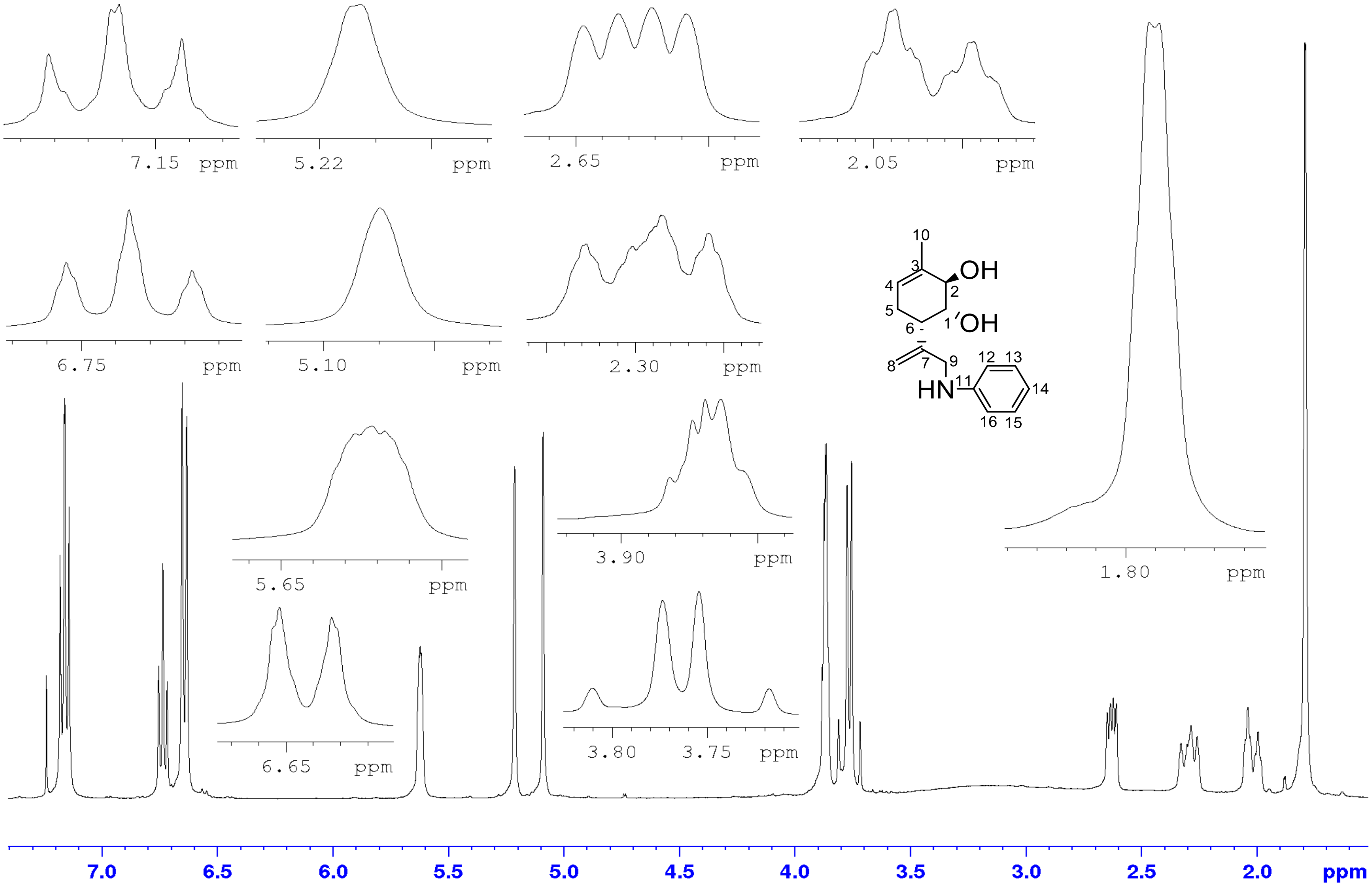

J-modulated ^{13}C NMR spectrum of (1R,2R,6S)-3-methyl-6-(3-(phenylamino)prop-1-en-2-yl)cyclohex-3-ene-1,2-diol (**26**)

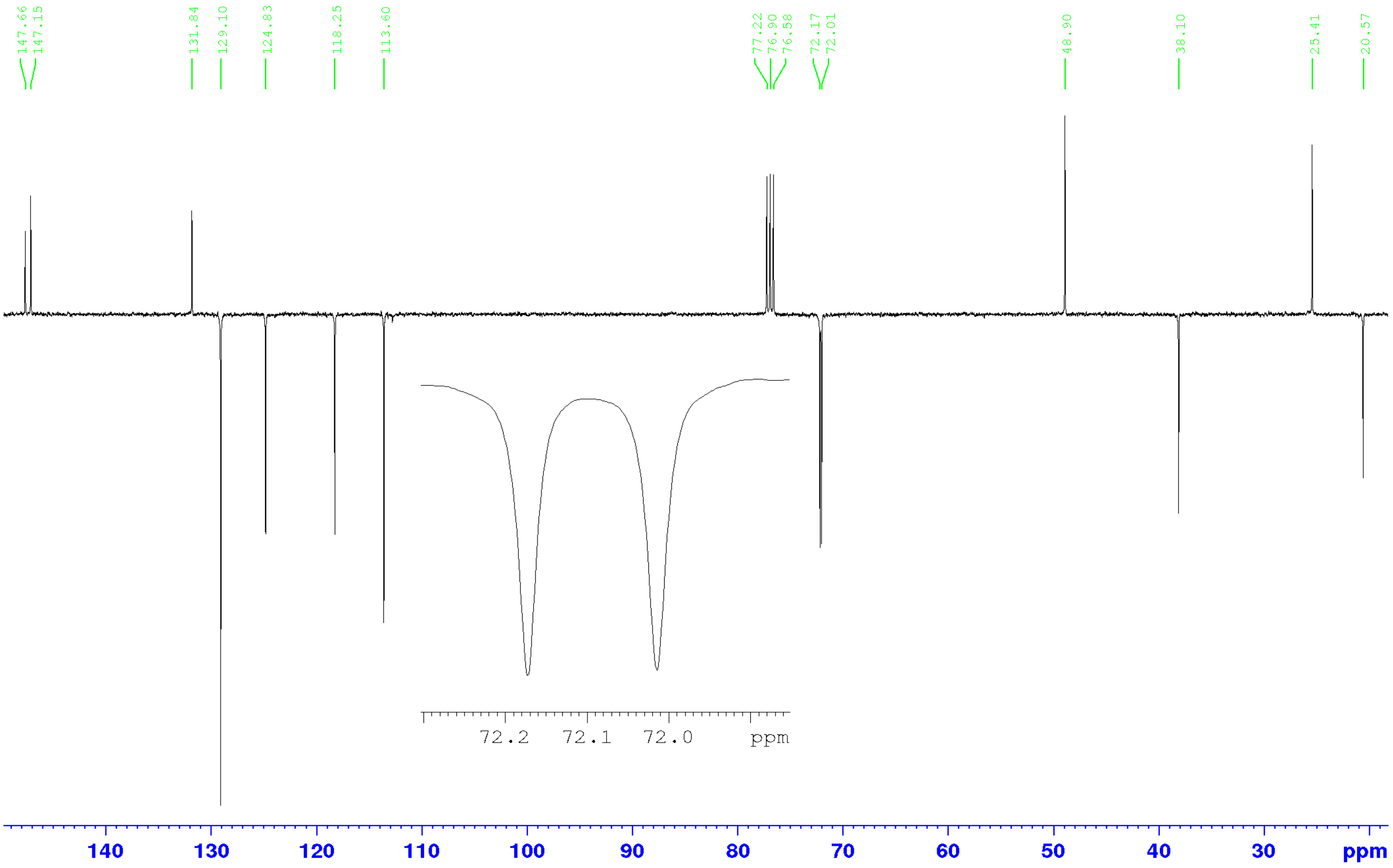

^{1}H-^{1}H 2D homonuclear correlation (COSY) spectrum of (1R,2R,6S)-3-methyl-6-(3-(phenylamino)prop-1-en-2-yl)cyclohex-3-ene-1,2-diol (**26**)

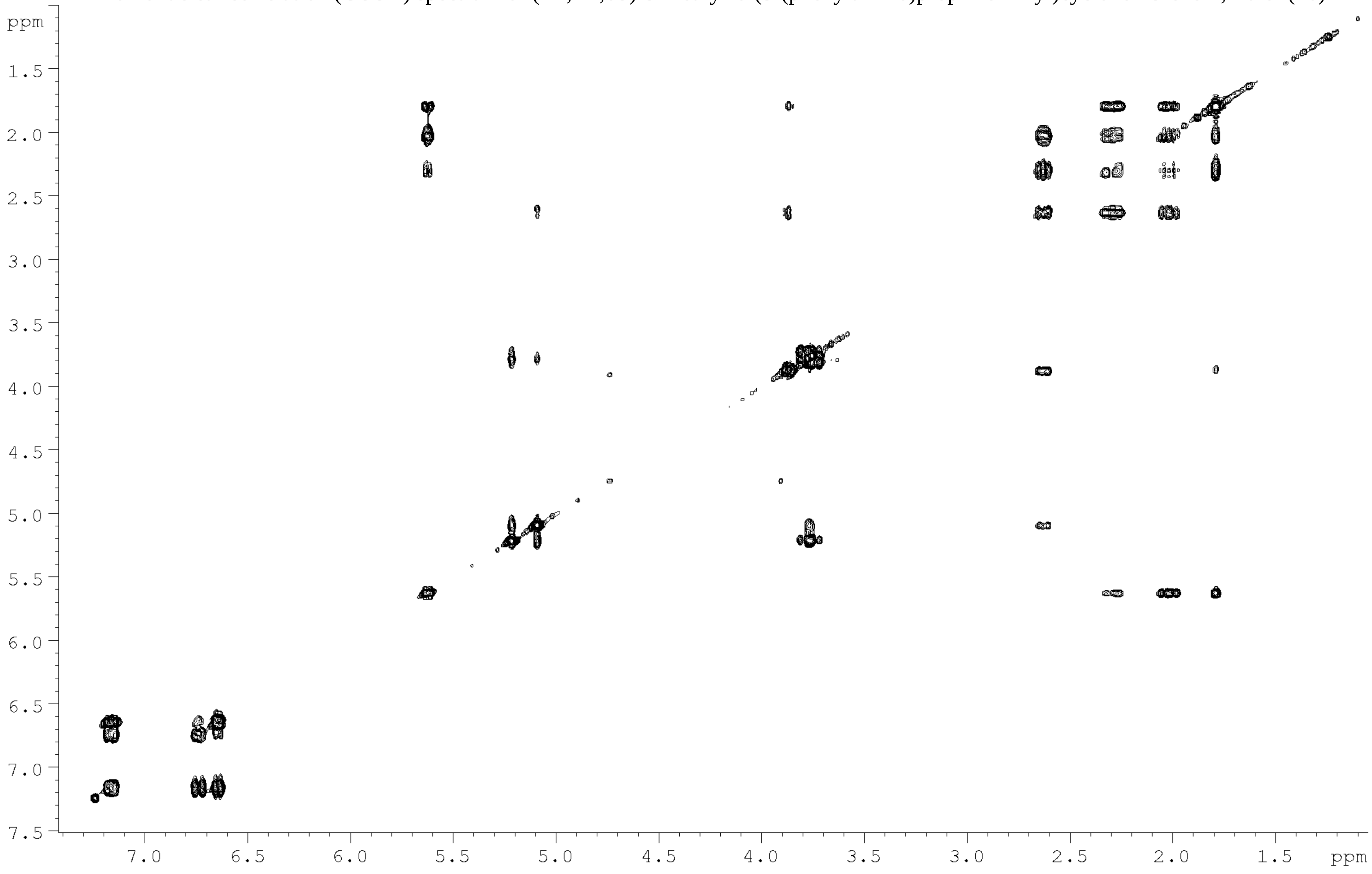

HSQC ^{13}C-^{1}H 2D heteronuclear correlation (C-H COSY) spectrum of (1R,2R,6S)-3-methyl-6-(3-(phenylamino)prop-1-en-2-yl)cyclohex-3-ene-1,2-diol (**26**)

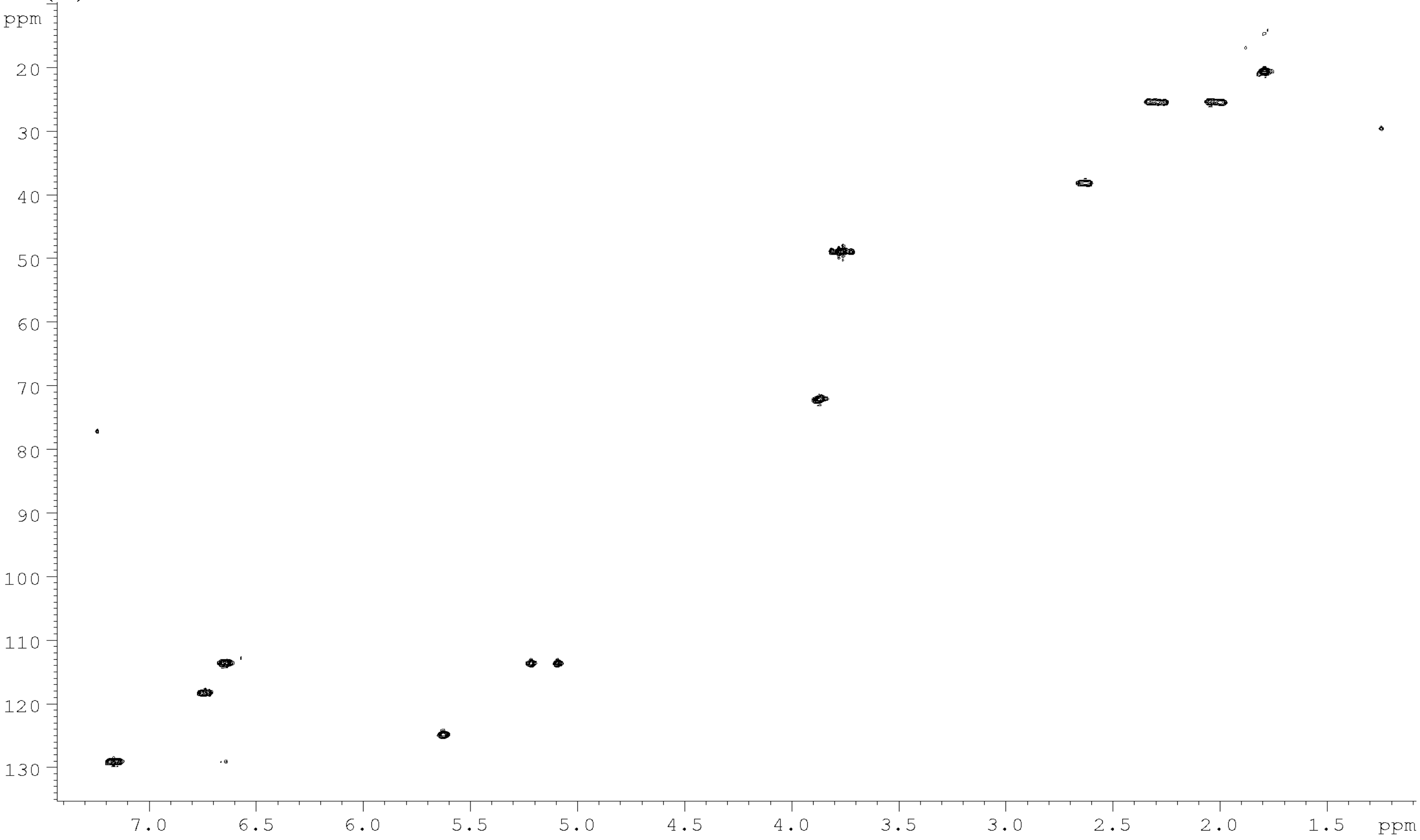

HMBC ^{13}C-^{1}H 2D heteronuclear correlation (C-H COSY) spectrum of (1R,2R,6S)-3-methyl-6-(3-(phenylamino)prop-1-en-2-yl)cyclohex-3-ene-1,2-diol (**26**)

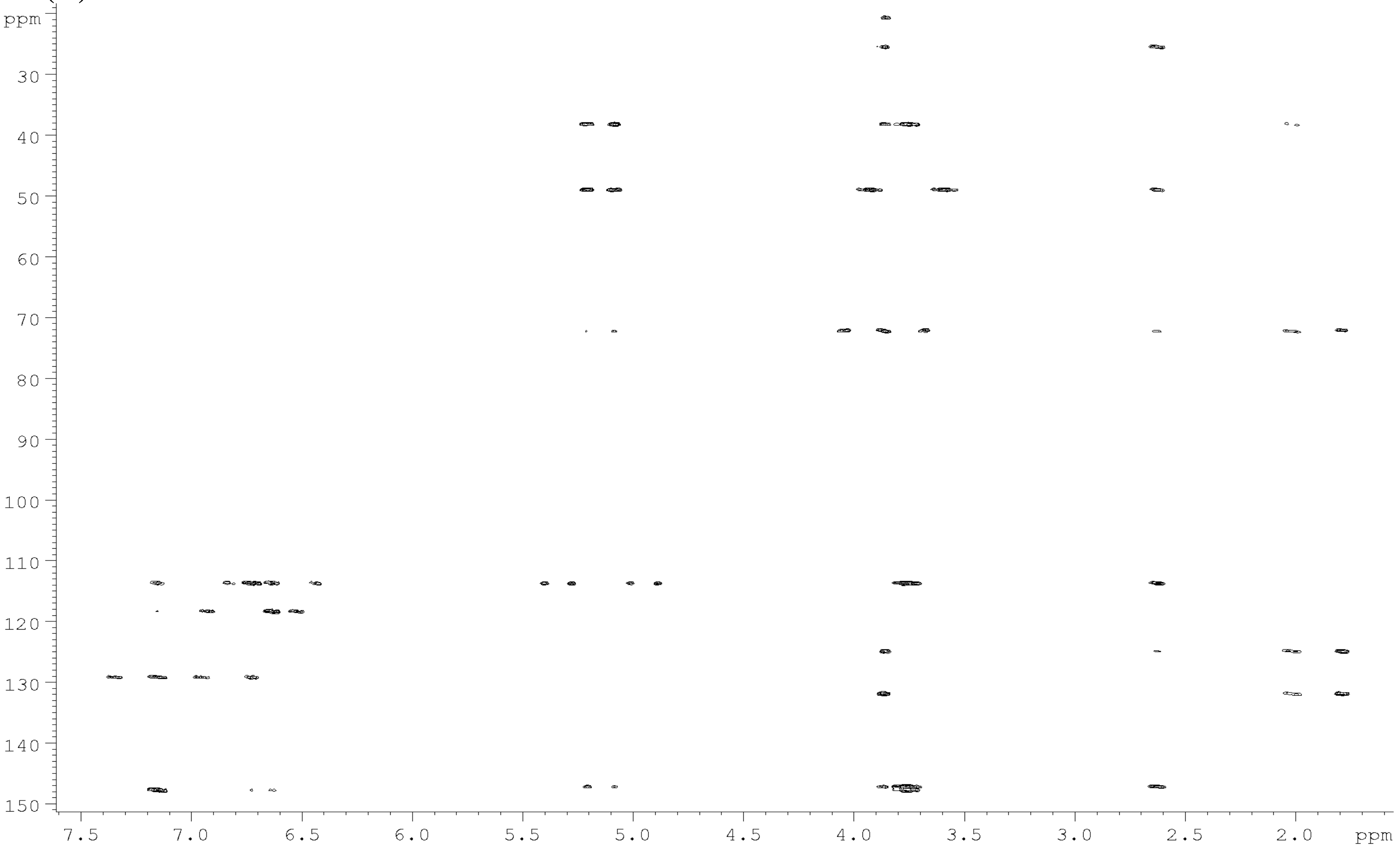

NOESY spectrum of (1R,2R,6S)-3-methyl-6-(3-(phenylamino)prop-1-en-2-yl)cyclohex-3-ene-1,2-diol (**26**)

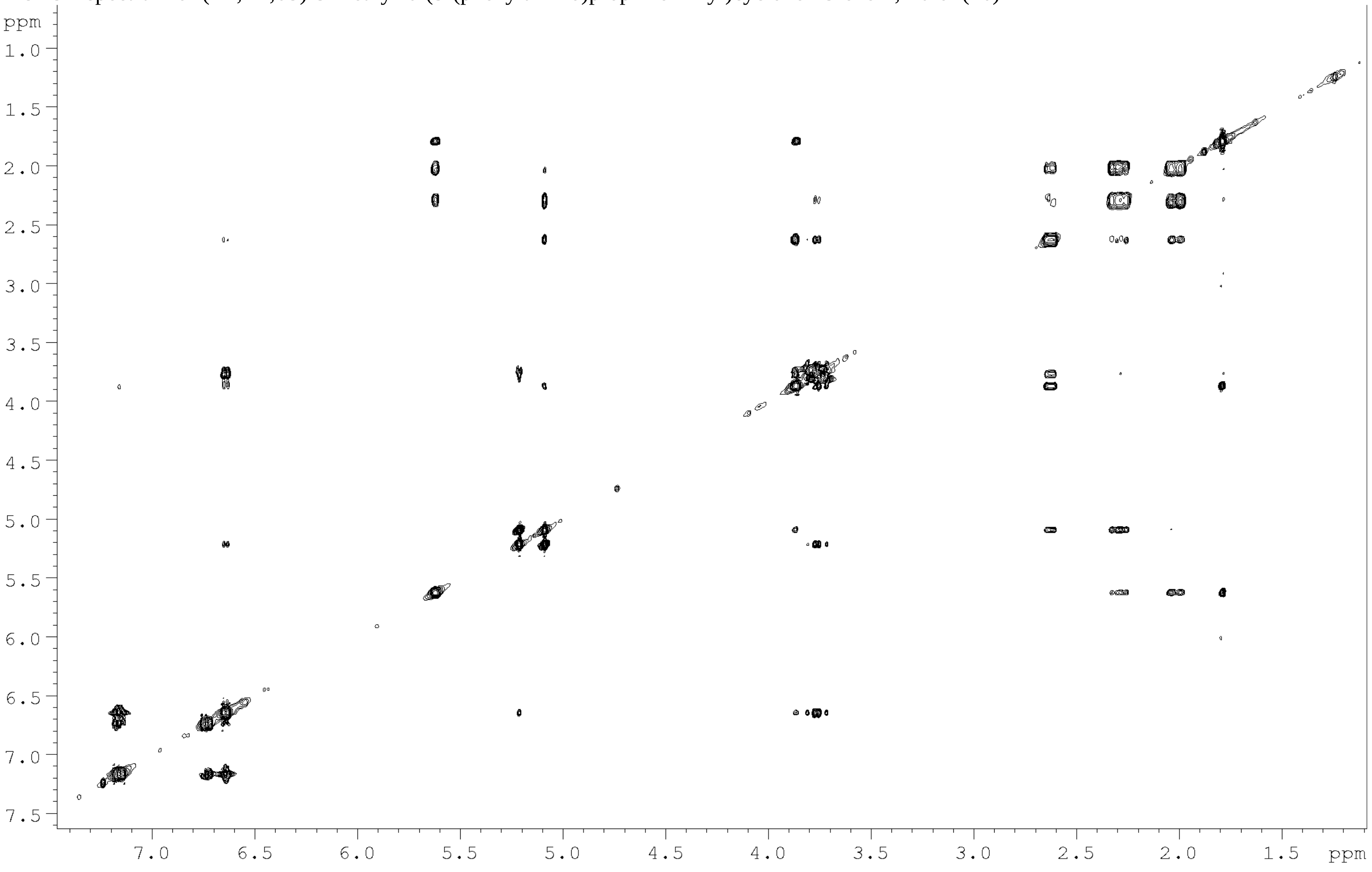

^{1}H NMR spectrum of (1R,2R,6S)-3-methyl-6-(3-(p-tolylamino)prop-1-en-2-yl)cyclohex-3-ene-1,2-diol (**27**)

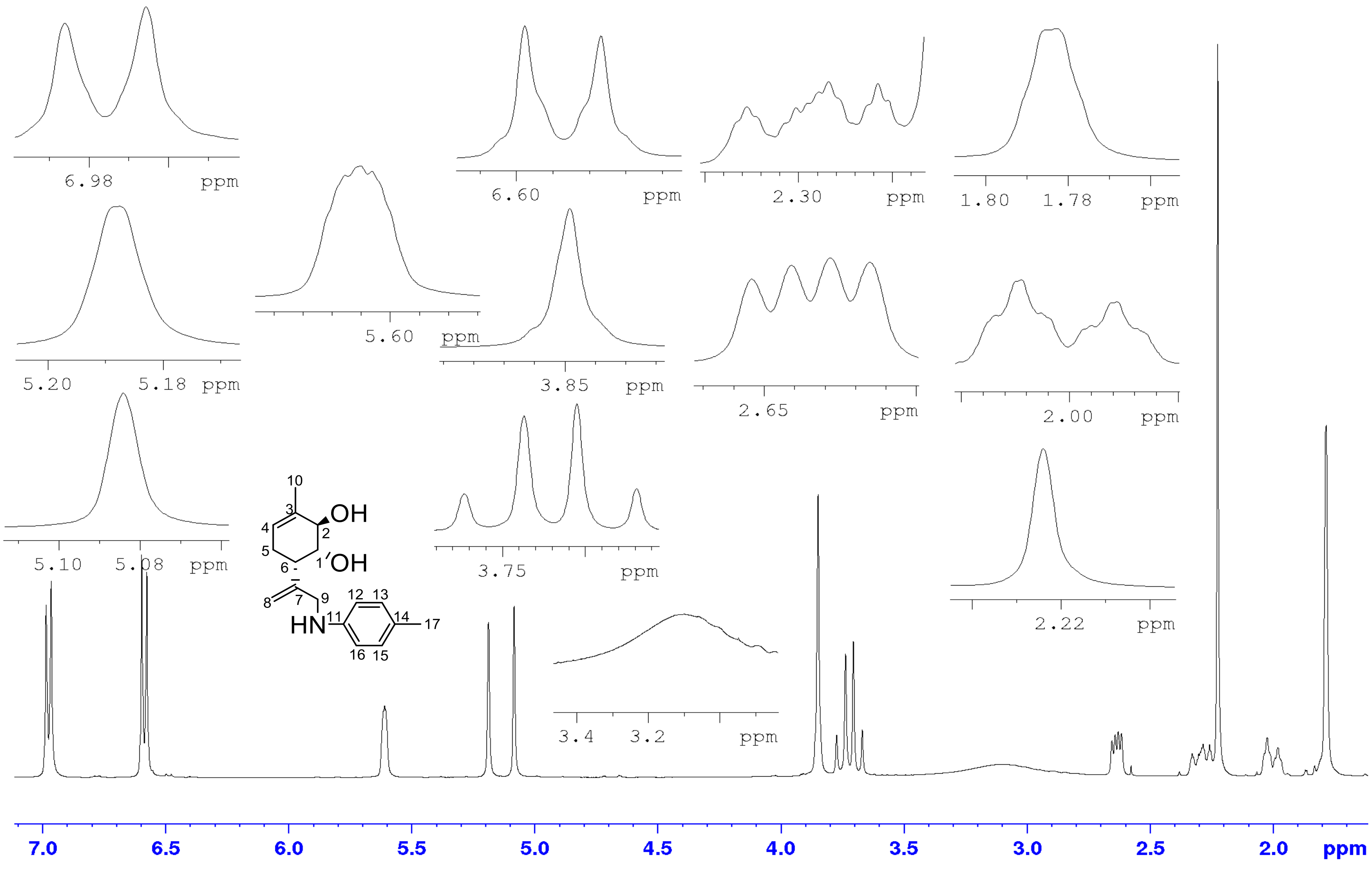

J-modulated ^{13}C NMR spectrum of (1R,2R,6S)-3-methyl-6-(3-(p-tolylamino)prop-1-en-2-yl)cyclohex-3-ene-1,2-diol (**27**)

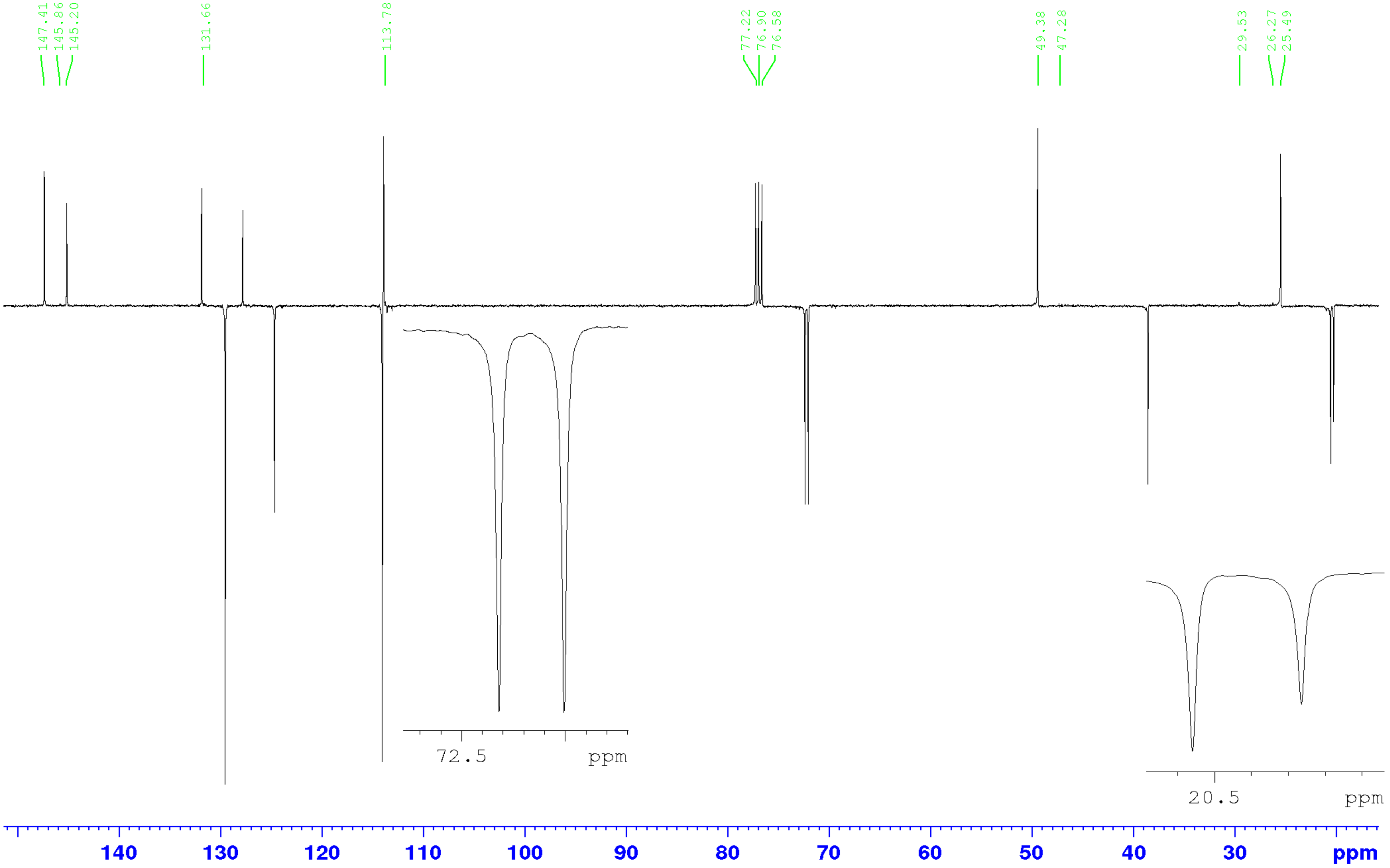

^{1}H-^{1}H 2D homonuclear correlation (COSY) spectrum of (1R,2R,6S)-3-methyl-6-(3-(p-tolylamino)prop-1-en-2-yl)cyclohex-3-ene-1,2-diol (**27**)

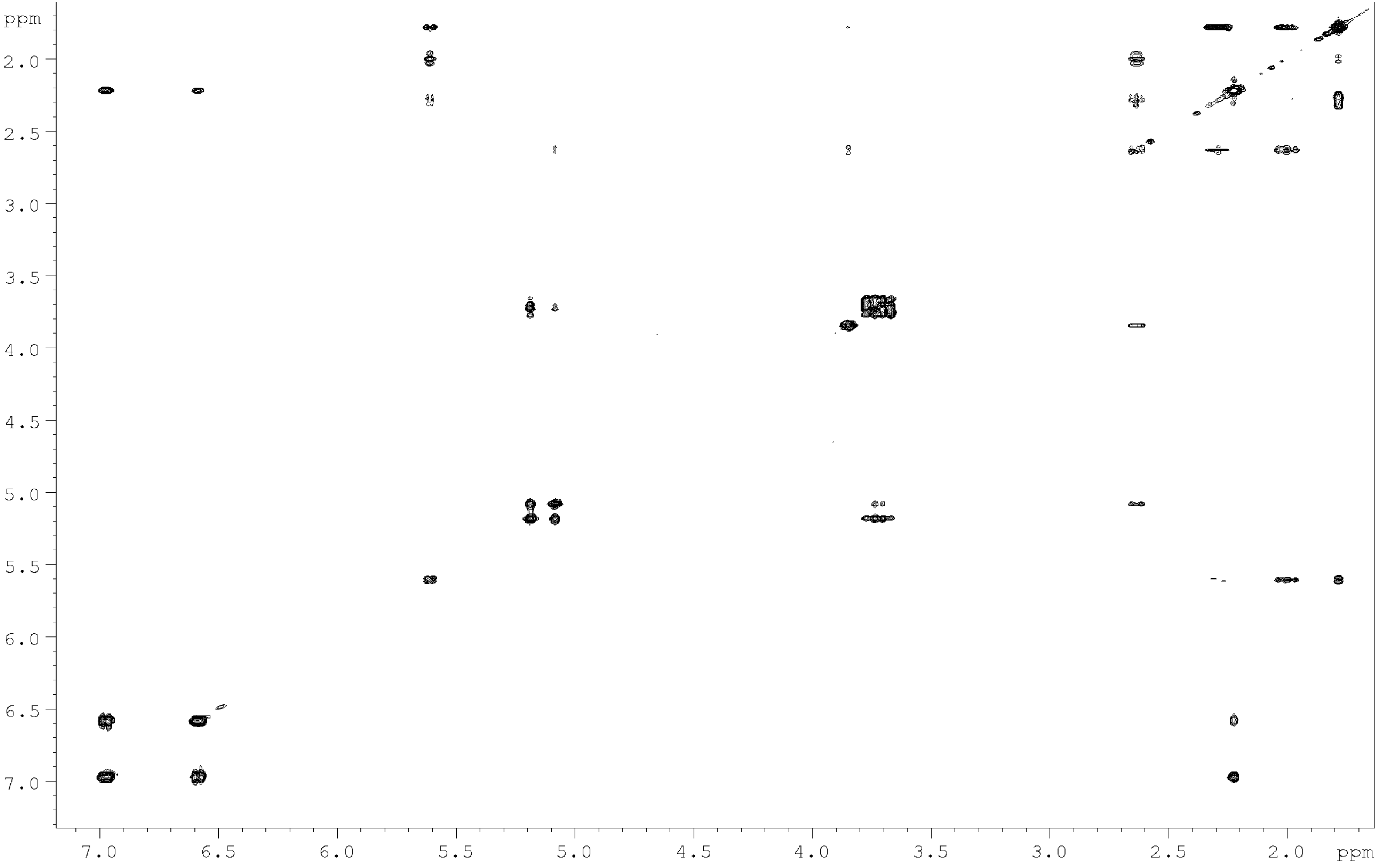

HSQC ^{13}C-^{1}H 2D heteronuclear correlation (C-H COSY) spectrum of (1R,2R,6S)-3-methyl-6-(3-(p-tolylamino)prop-1-en-2-yl)cyclohex-3-ene-1,2-diol (**27**)

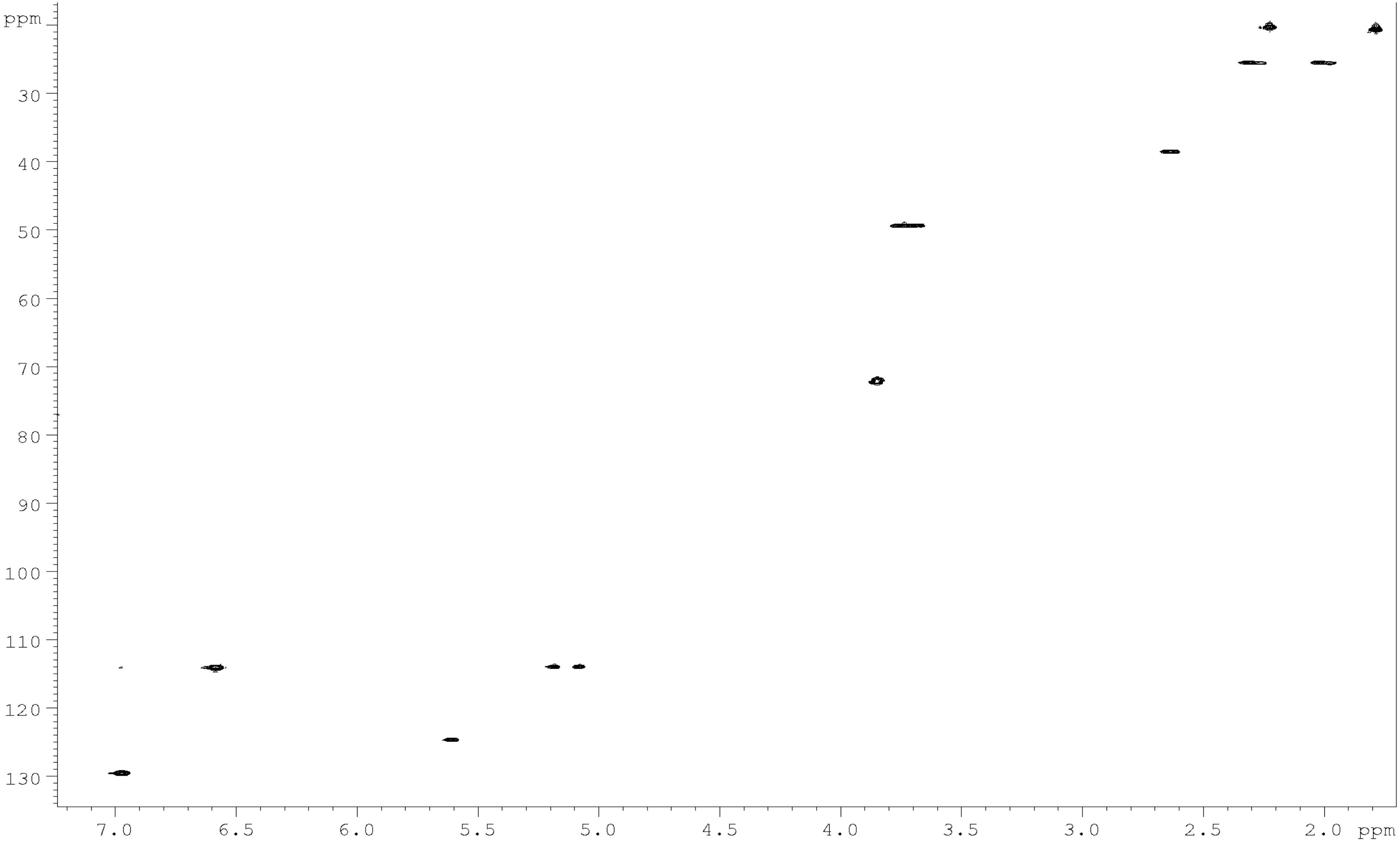

HMBC ^{13}C-^{1}H 2D heteronuclear correlation (C-H COSY) spectrum of (1R,2R,6S)-3-methyl-6-(3-(p-tolylamino)prop-1-en-2-yl)cyclohex-3-ene-1,2-diol (**27**)

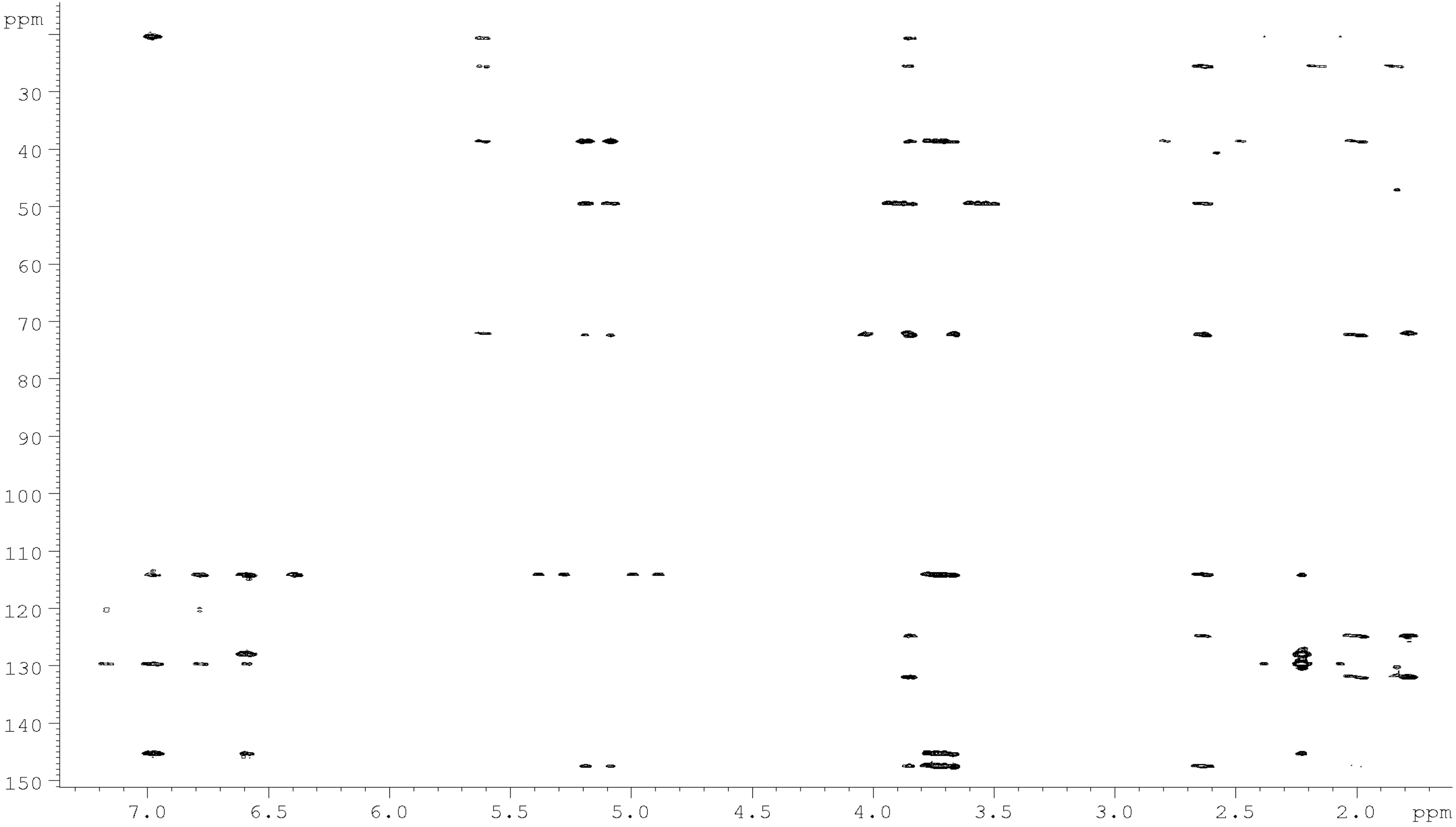

NOESY spectrum of (1R,2R,6S)-3-methyl-6-(3-(p-tolylamino)prop-1-en-2-yl)cyclohex-3-ene-1,2-diol (**27**)

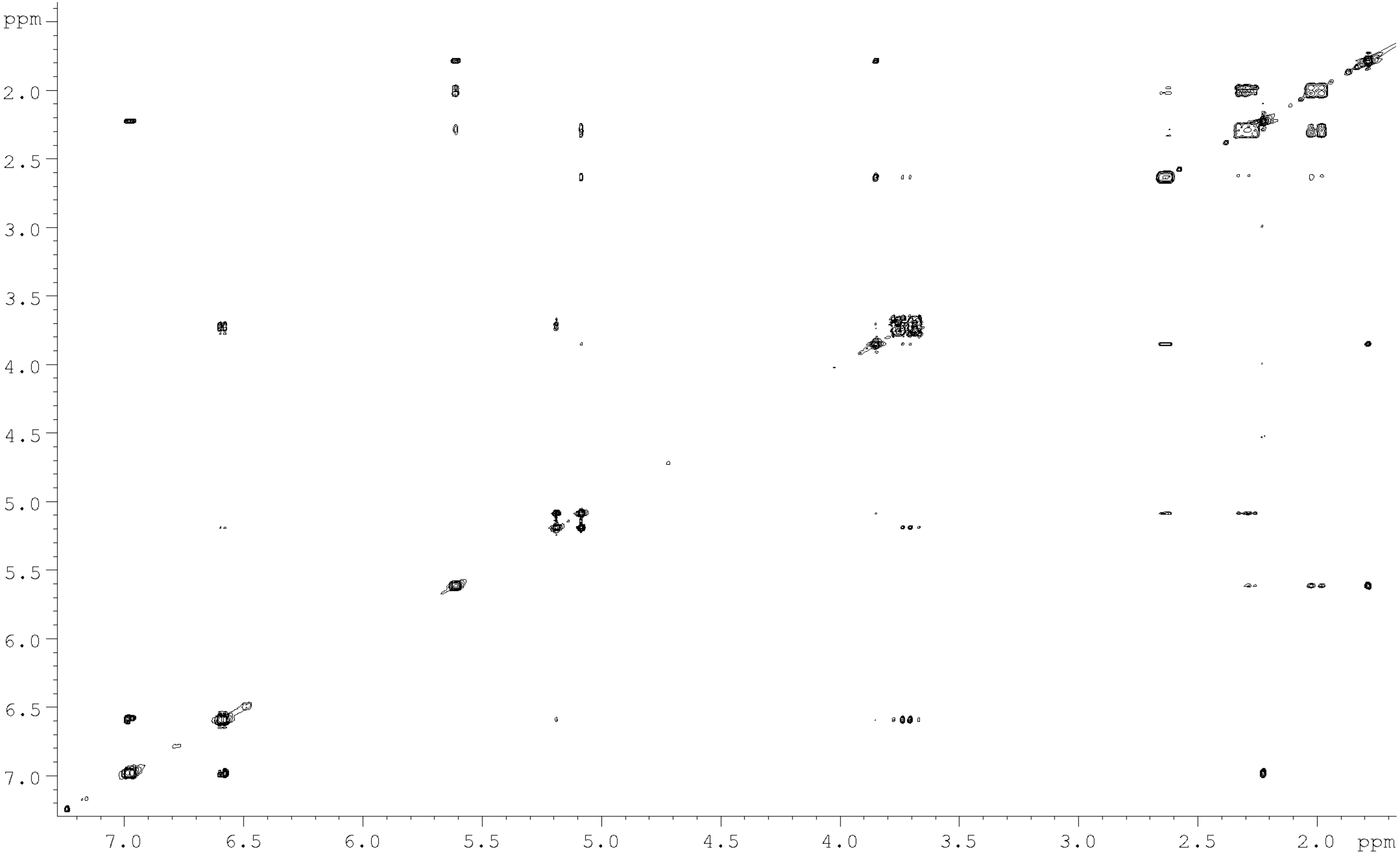

^{1}H NMR spectrum of (1R,2R,6S)-6-(3-(2,4-dimethylphenylamino)prop-1-en-2-yl)-3-methylcyclohex-3-ene-1,2-diol (**28**)

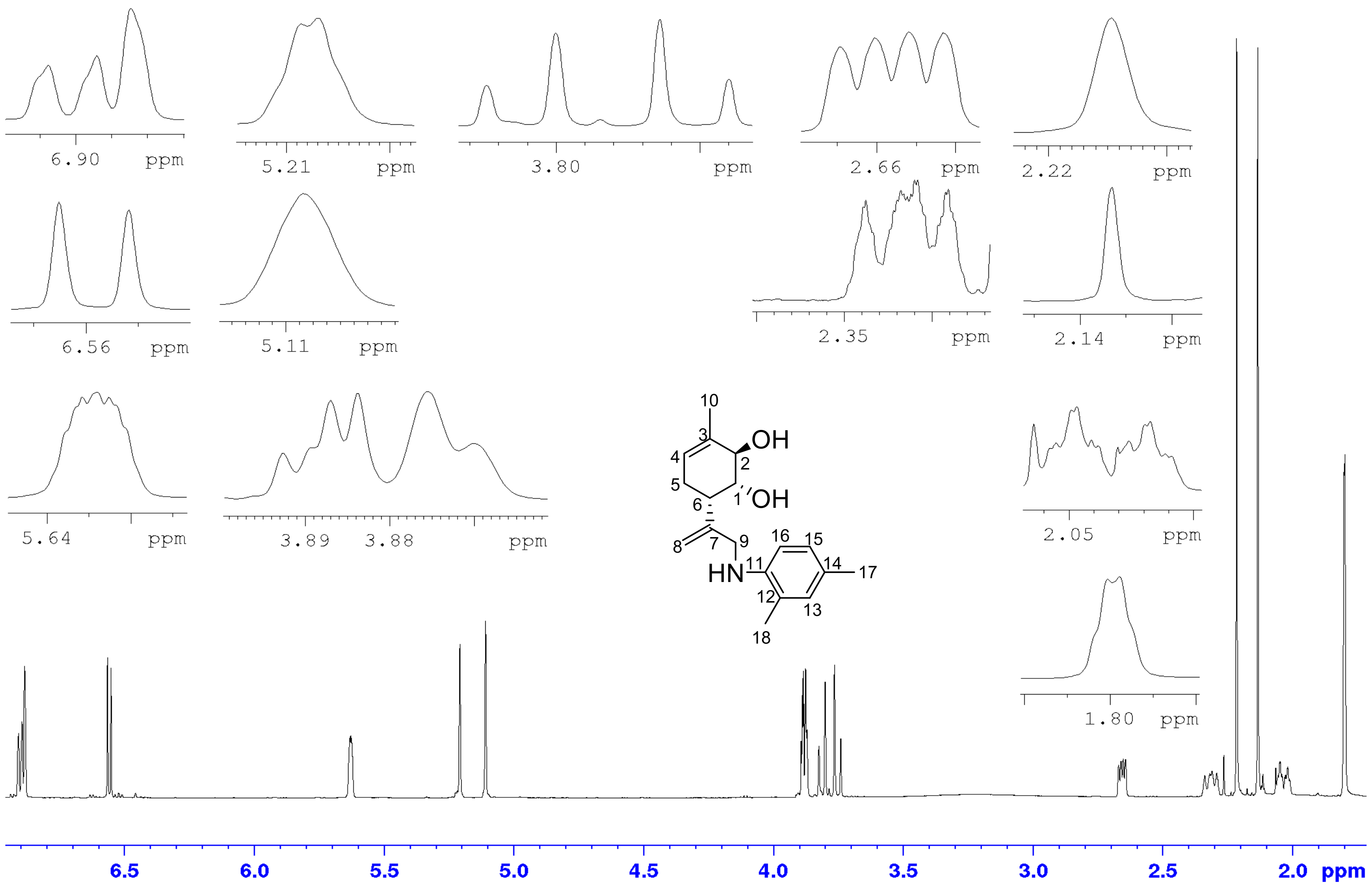

J-modulated ^{13}C NMR spectrum of (1R,2R,6S)-6-(3-(2,4-dimethylphenylamino)prop-1-en-2-yl)-3-methylcyclohex-3-ene-1,2-diol (**28**)

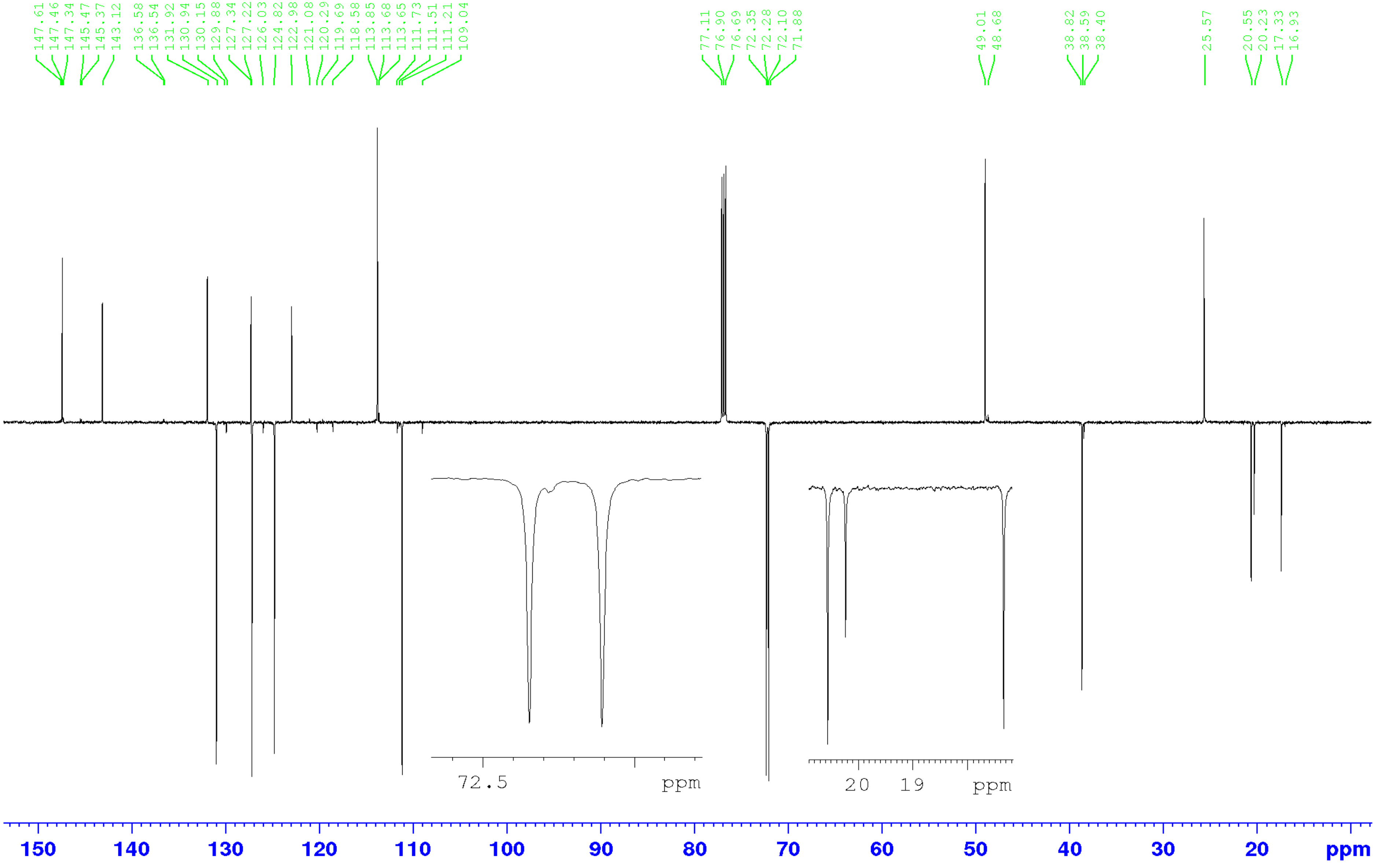

^{1}H-^{1}H 2D homonuclear correlation (COSY) spectrum of (1R,2R,6S)-6-(3-(2,4-dimethylphenylamino)prop-1-en-2-yl)-3-methylcyclohex-3-ene-1,2-diol (**28**)

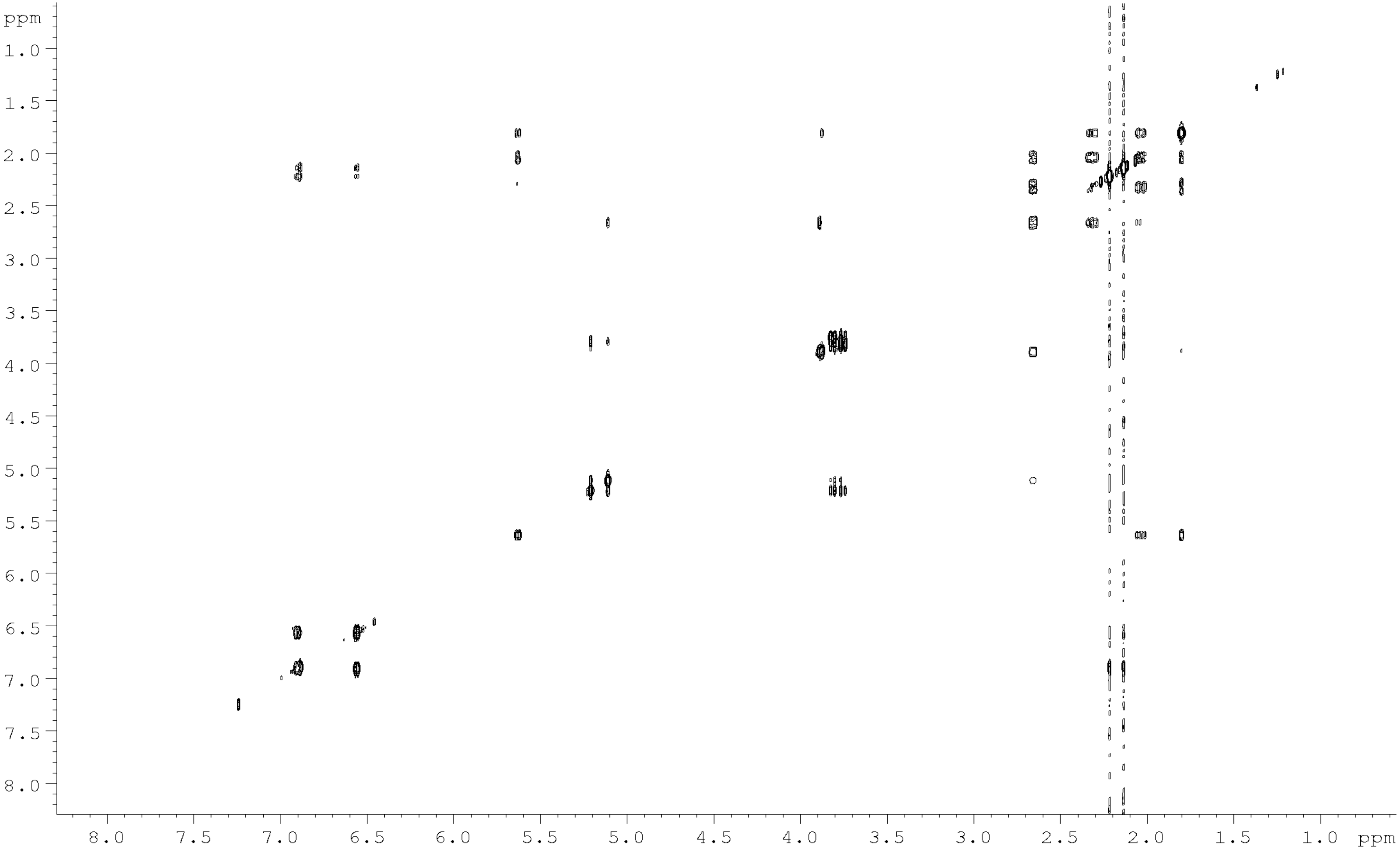

HSQC ^{13}C-^{1}H 2D heteronuclear correlation (C-H COSY) spectrum of (1R,2R,6S)-6-(3-(2,4-dimethylphenylamino)prop-1-en-2-yl)-3-methylcyclohex-3-ene-1,2-diol (**28**)

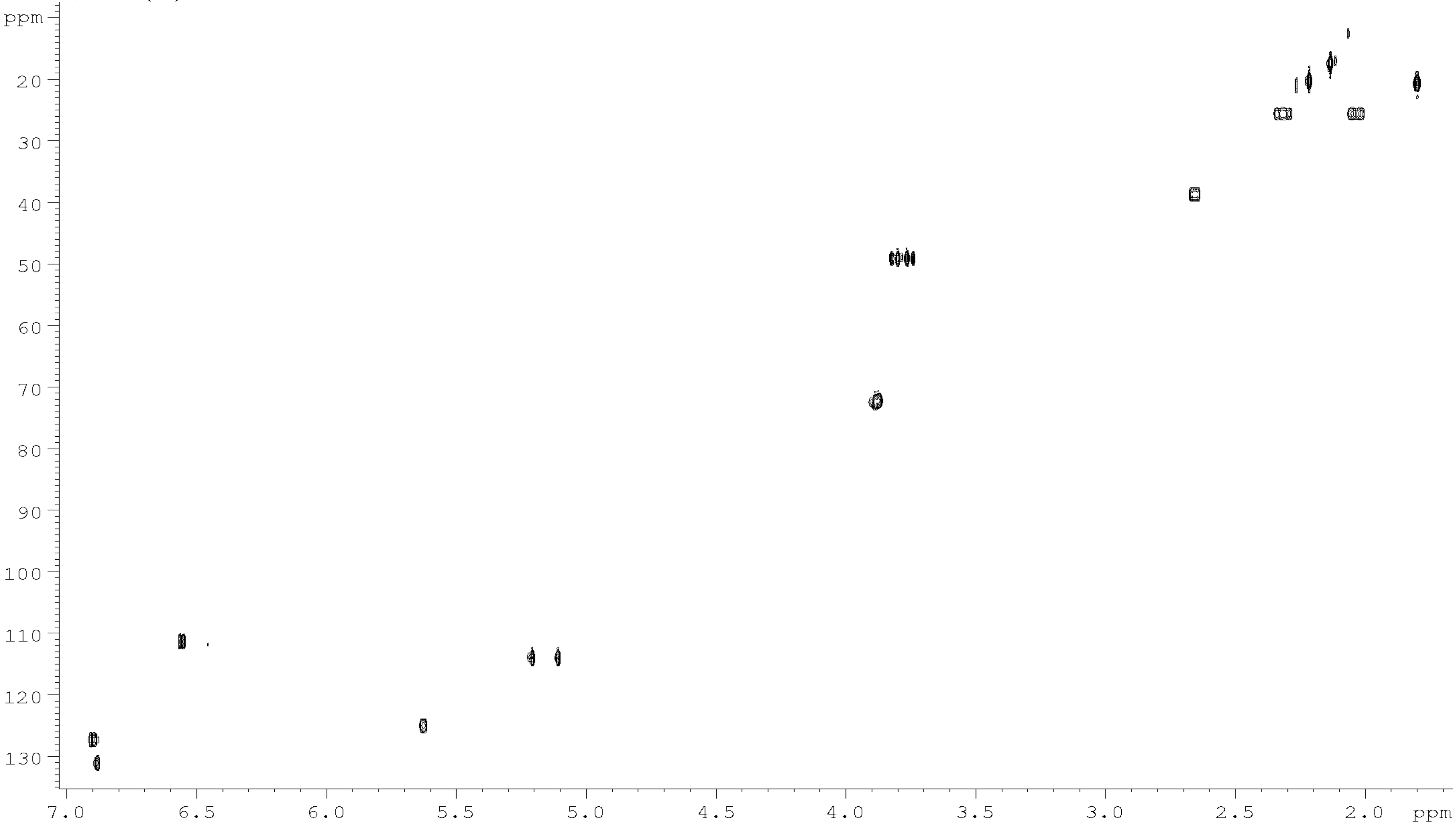

HMBC ^{13}C-^{1}H 2D heteronuclear correlation (C-H COSY) spectrum of (1R,2R,6S)-6-(3-(2,4-dimethylphenylamino)prop-1-en-2-yl)-3-methylcyclohex-3-ene-1,2-diol (**28**)

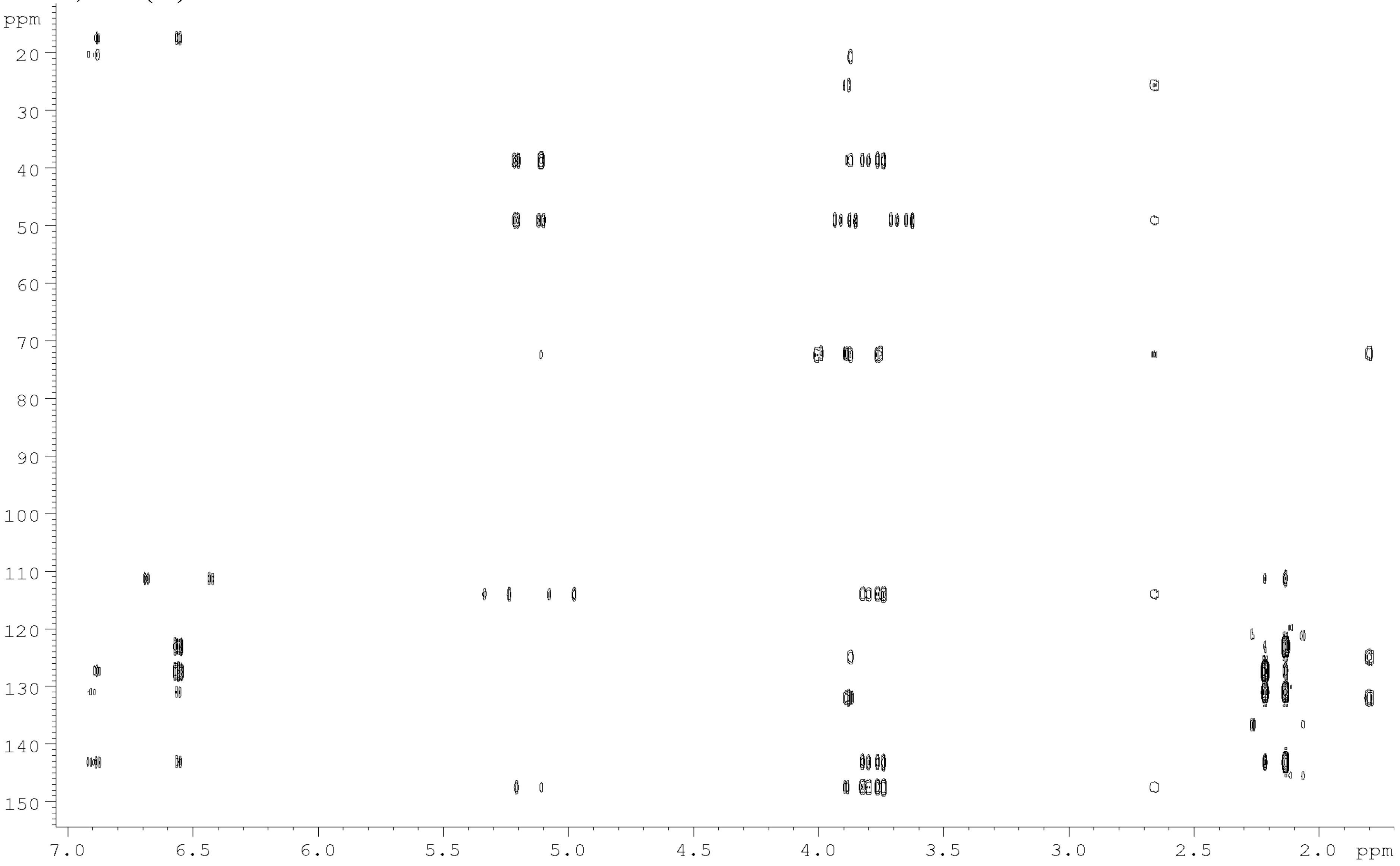

NOESY spectrum of (1R,2R,6S)-6-(3-(2,4-dimethylphenylamino)prop-1-en-2-yl)-3-methylcyclohex-3-ene-1,2-diol (**28**)

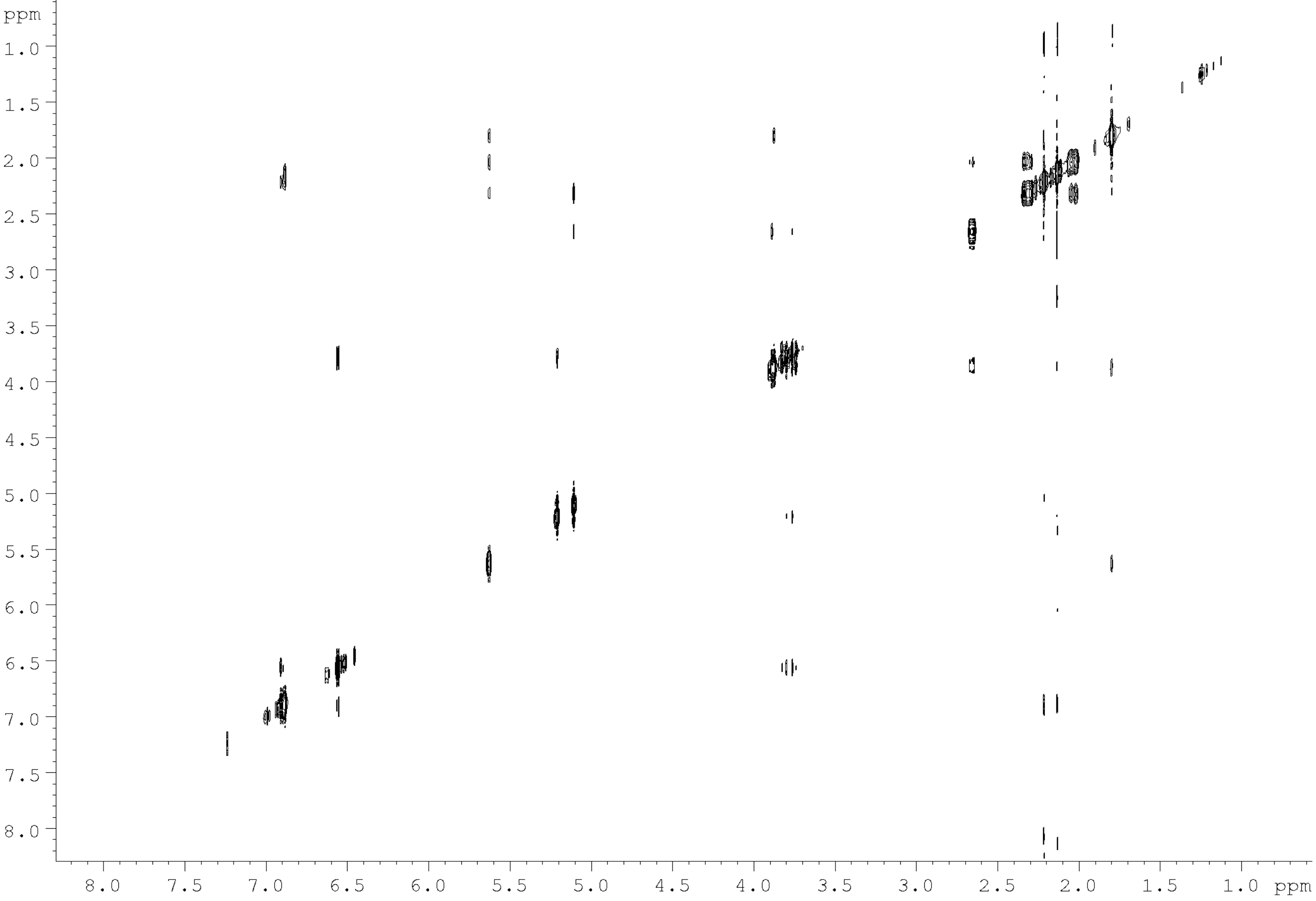

^{1}H NMR spectrum of (1R,2R,6S)-6-(3-(diethylamino)prop-1-en-2-yl)-3-methylcyclohex-3-ene-1,2-diol (**29**)

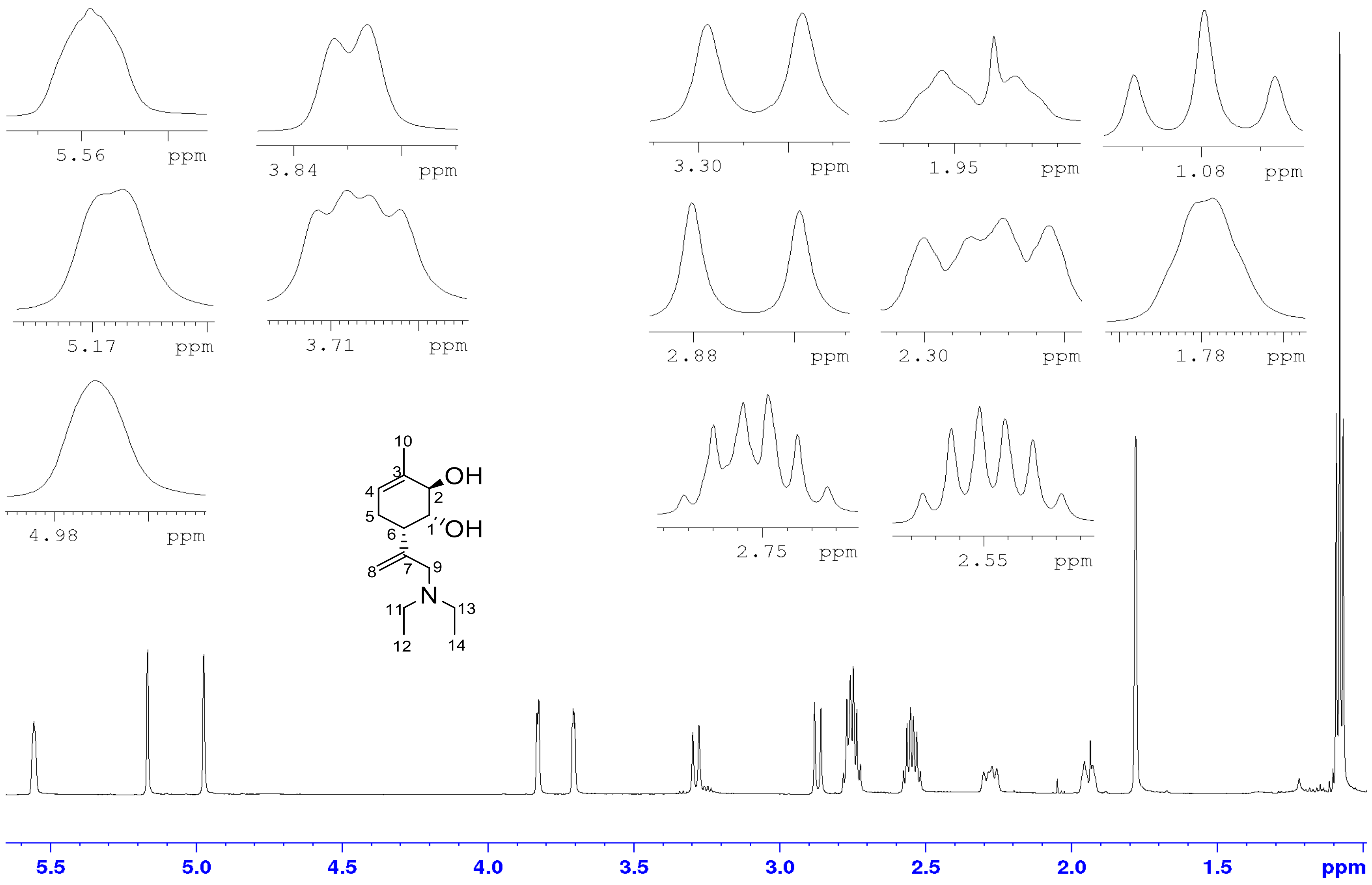

J-modulated ^{13}C NMR spectrum of (1R,2R,6S)-6-(3-(diethylamino)prop-1-en-2-yl)-3-methylcyclohex-3-ene-1,2-diol (**29**)

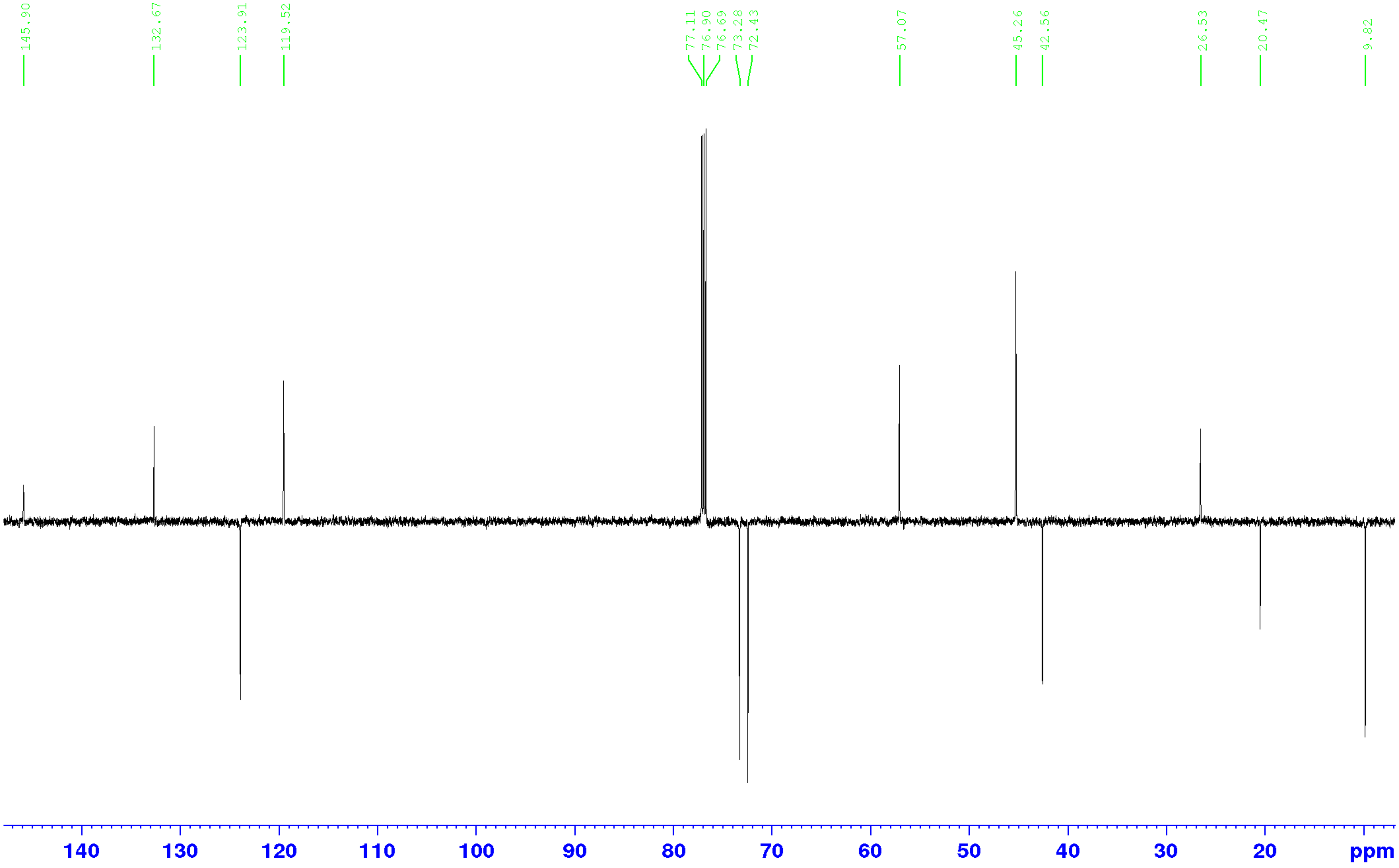

^{1}H-^{1}H 2D homonuclear correlation (COSY) spectrum of (1R,2R,6S)-6-(3-(diethylamino)prop-1-en-2-yl)-3-methylcyclohex-3-ene-1,2-diol (**29**)

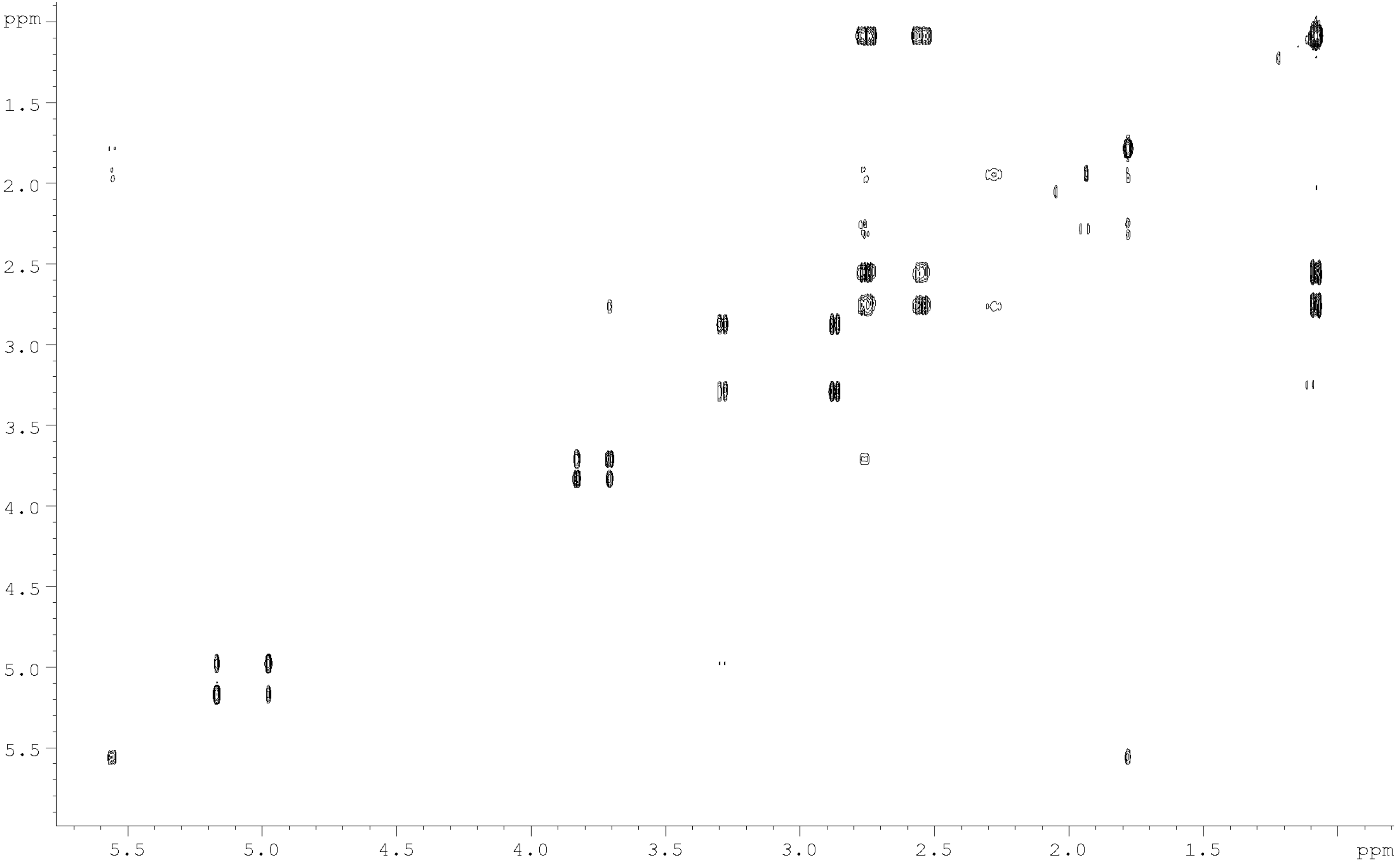

HSQC ^{13}C-^{1}H 2D heteronuclear correlation (C-H COSY) spectrum of (1R,2R,6S)-6-(3-(diethylamino)prop-1-en-2-yl)-3-methylcyclohex-3-ene-1,2-diol (**29**)

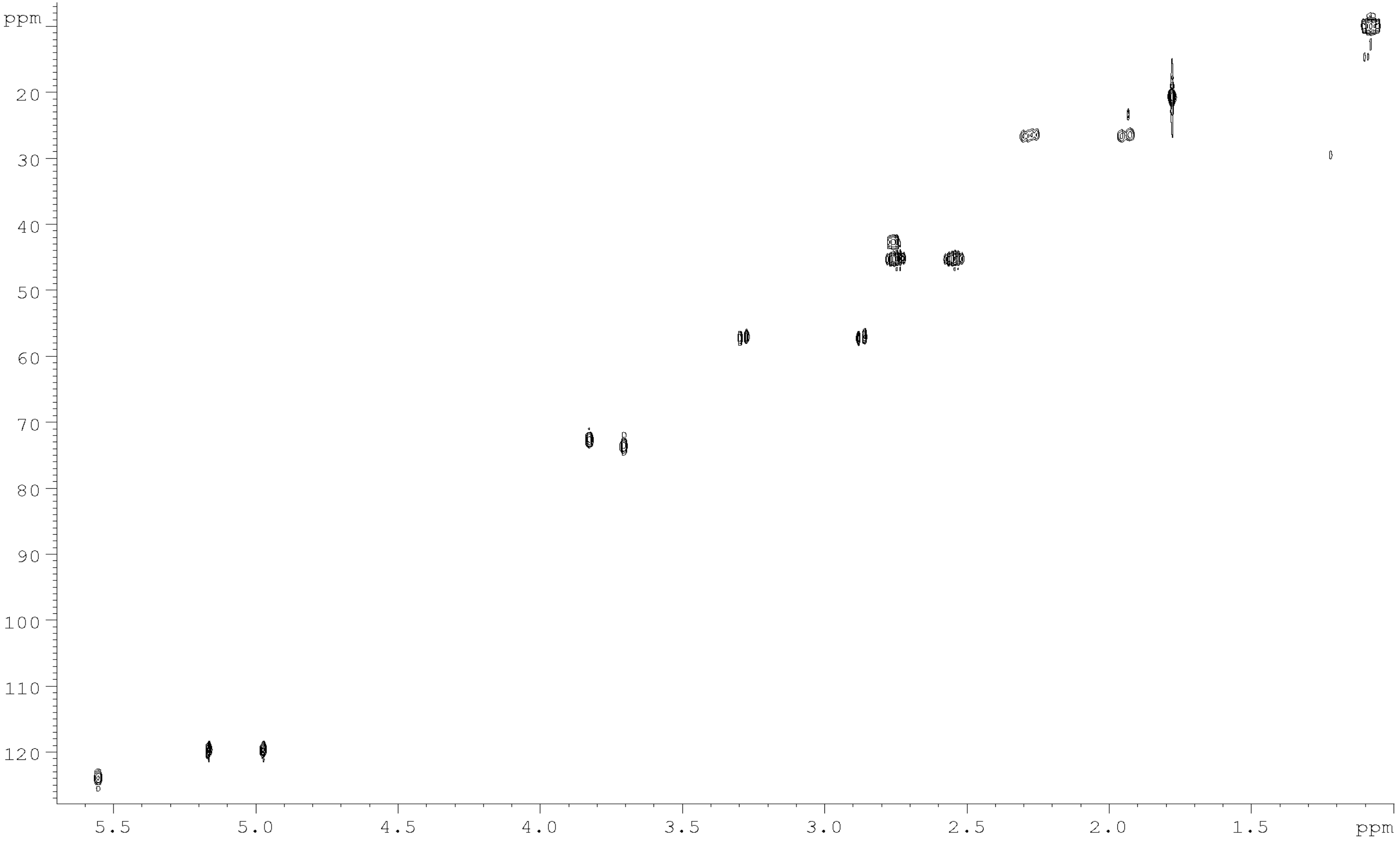

HMBC ^{13}C-^{1}H 2D heteronuclear correlation (C-H COSY) spectrum of (1R,2R,6S)-6-(3-(diethylamino)prop-1-en-2-yl)-3-methylcyclohex-3-ene-1,2-diol (**29**)

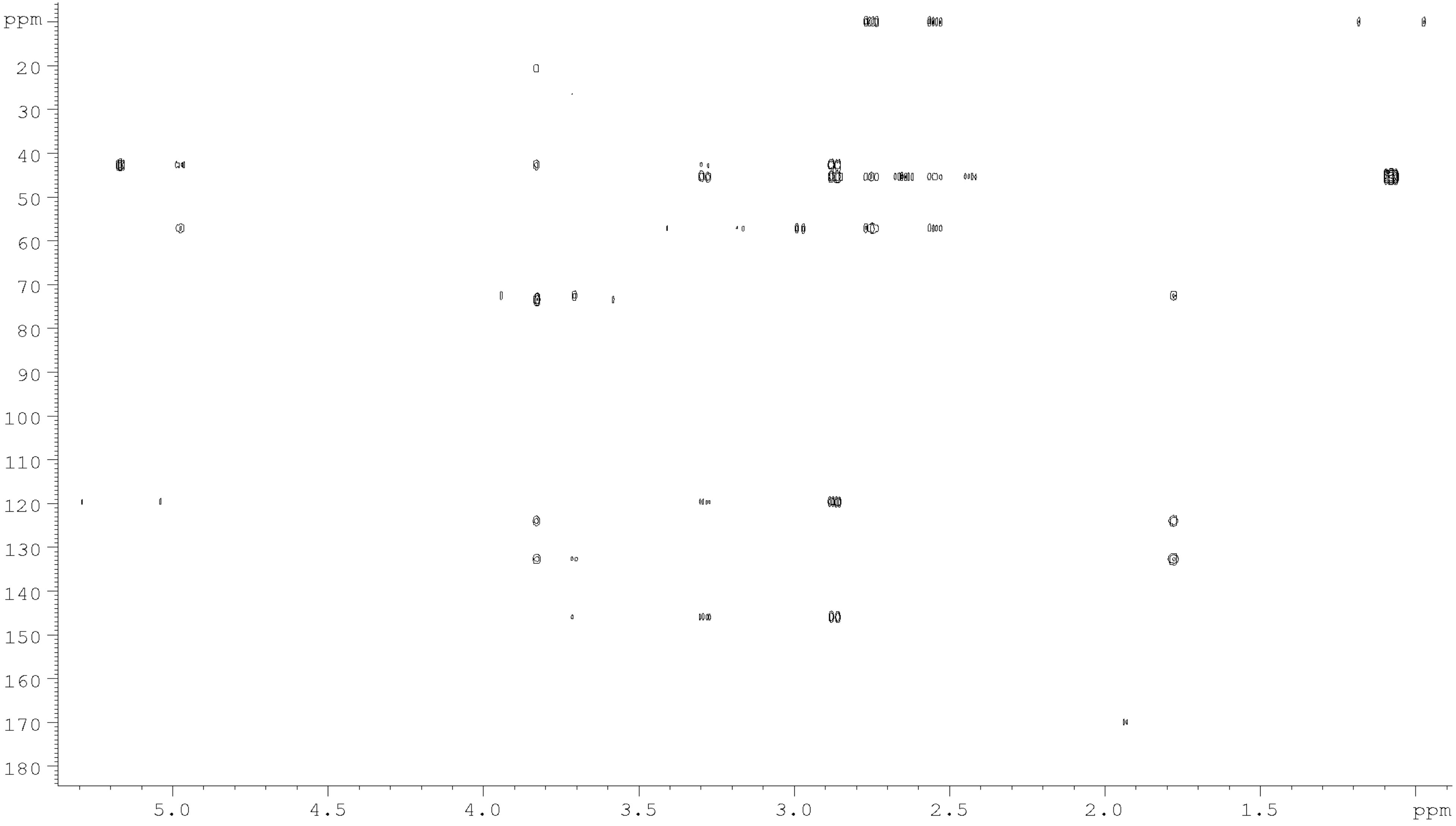

NOESY spectrum of (1R,2R,6S)-6-(3-(diethylamino)prop-1-en-2-yl)-3-methylcyclohex-3-ene-1,2-diol (**29**)

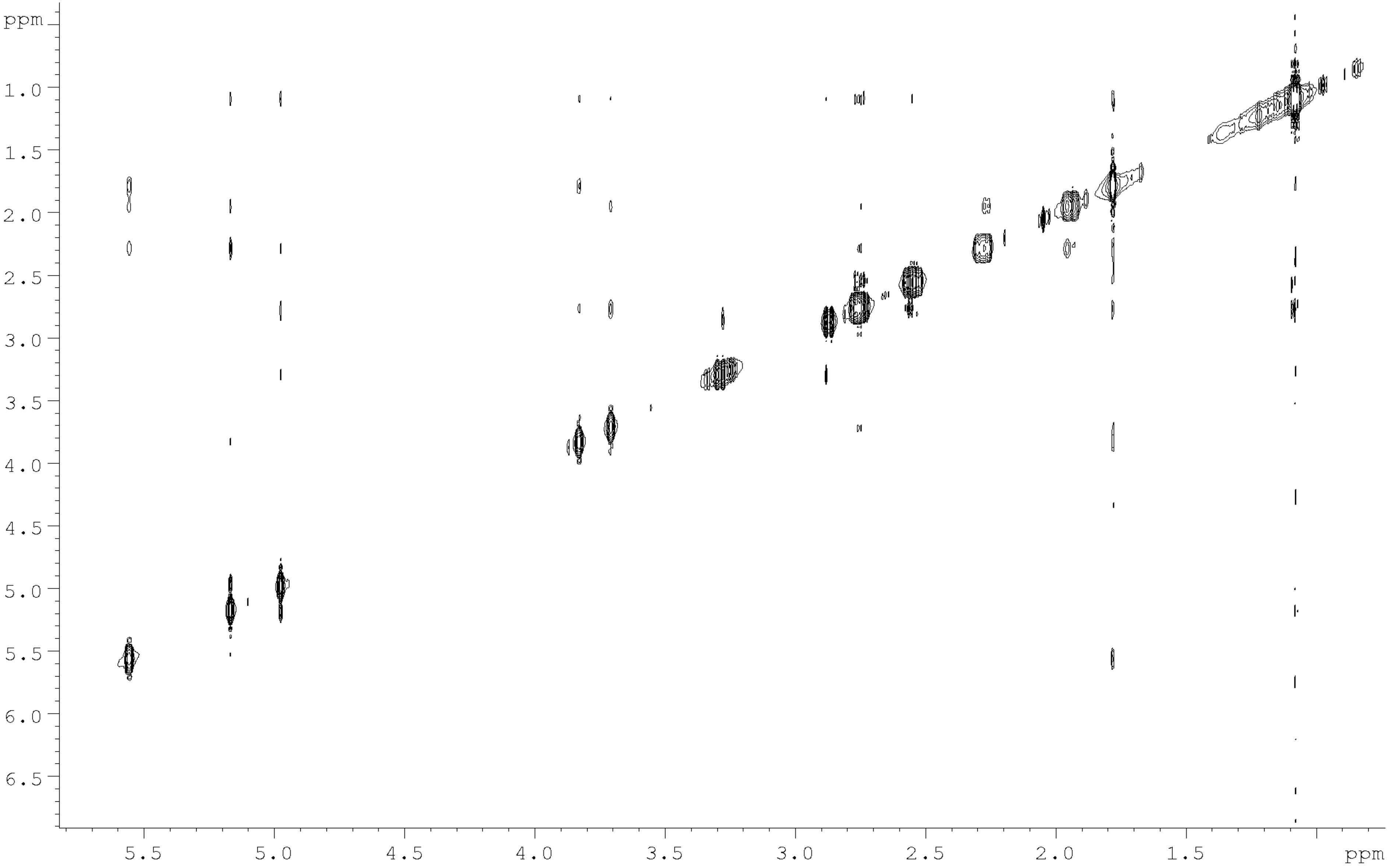

^{1}H NMR spectrum of 1-(2-(2-((1S,5R,6R)-5,6-dihydroxy-4-methylcyclohex-3-enyl)allylamino)phenyl)ethanone (**30**)

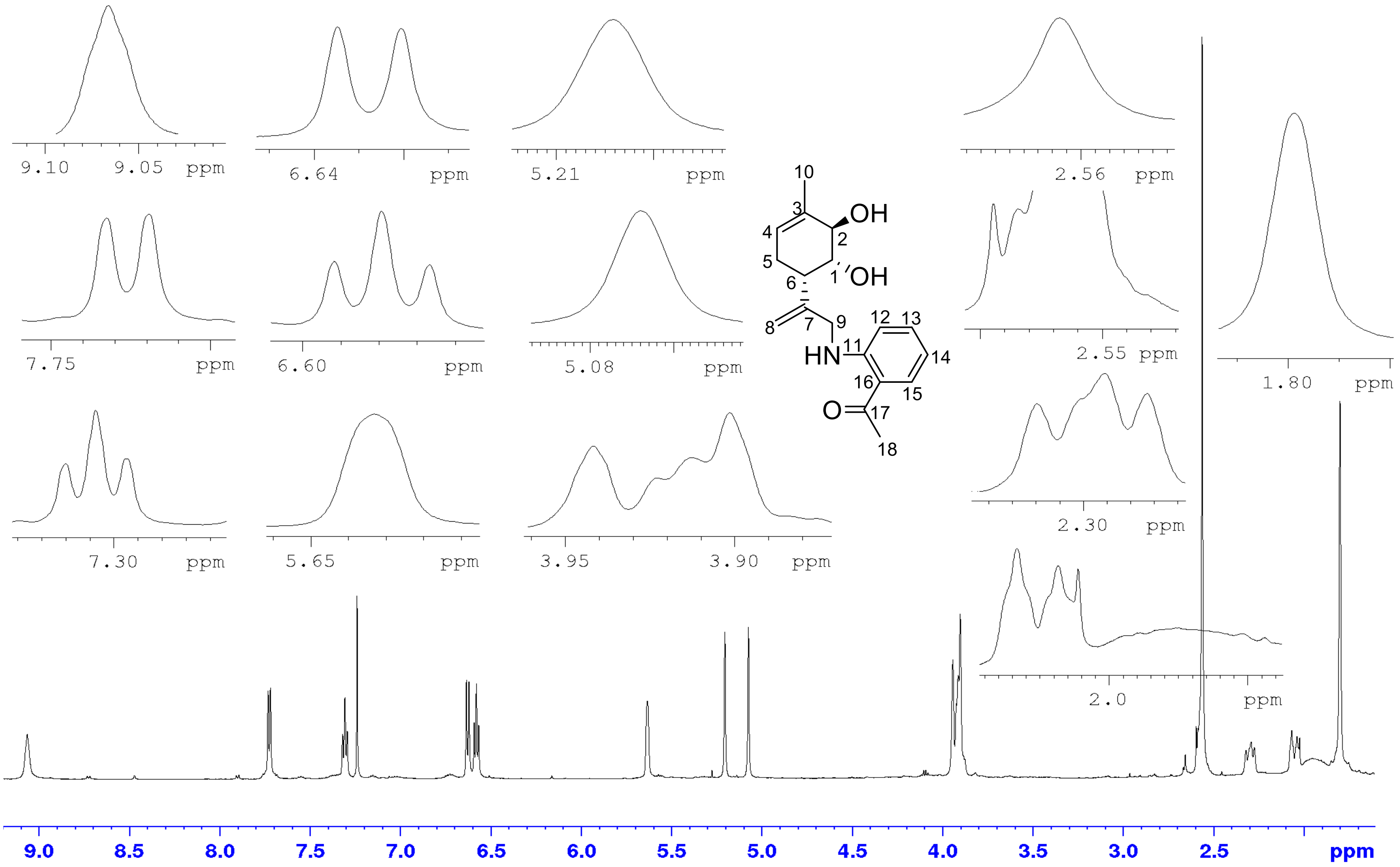

J-modulated ^{13}C NMR spectrum of 1-(2-(2-((1S,5R,6R)-5,6-dihydroxy-4-methylcyclohex-3-enyl)allylamino)phenyl)ethanone (**30**)

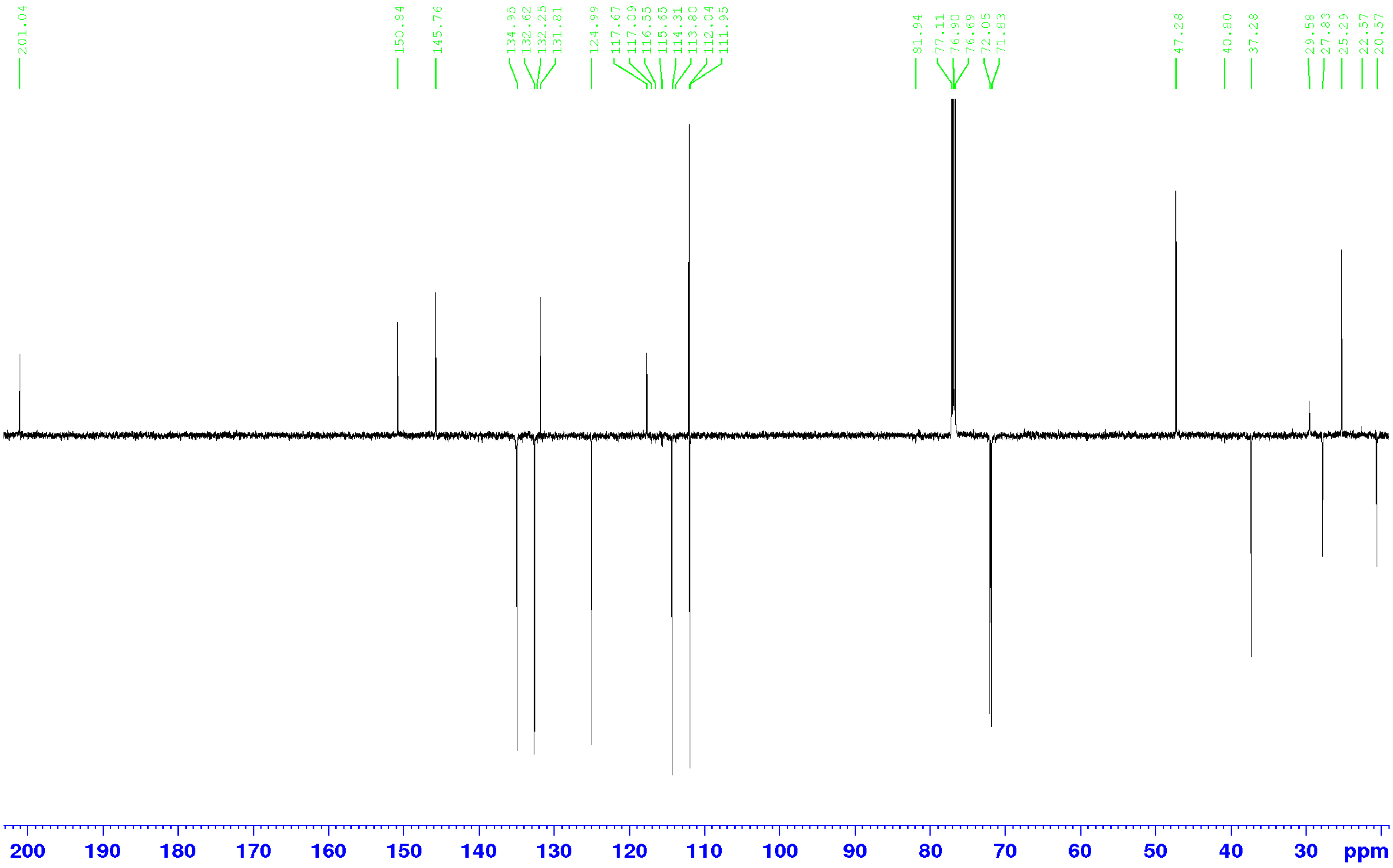

^{1}H-^{1}H 2D homonuclear correlation (COSY) spectrum of 1-(2-(2-((1S,5R,6R)-5,6-dihydroxy-4-methylcyclohex-3-enyl)allylamino)phenyl)ethanone (**30**)

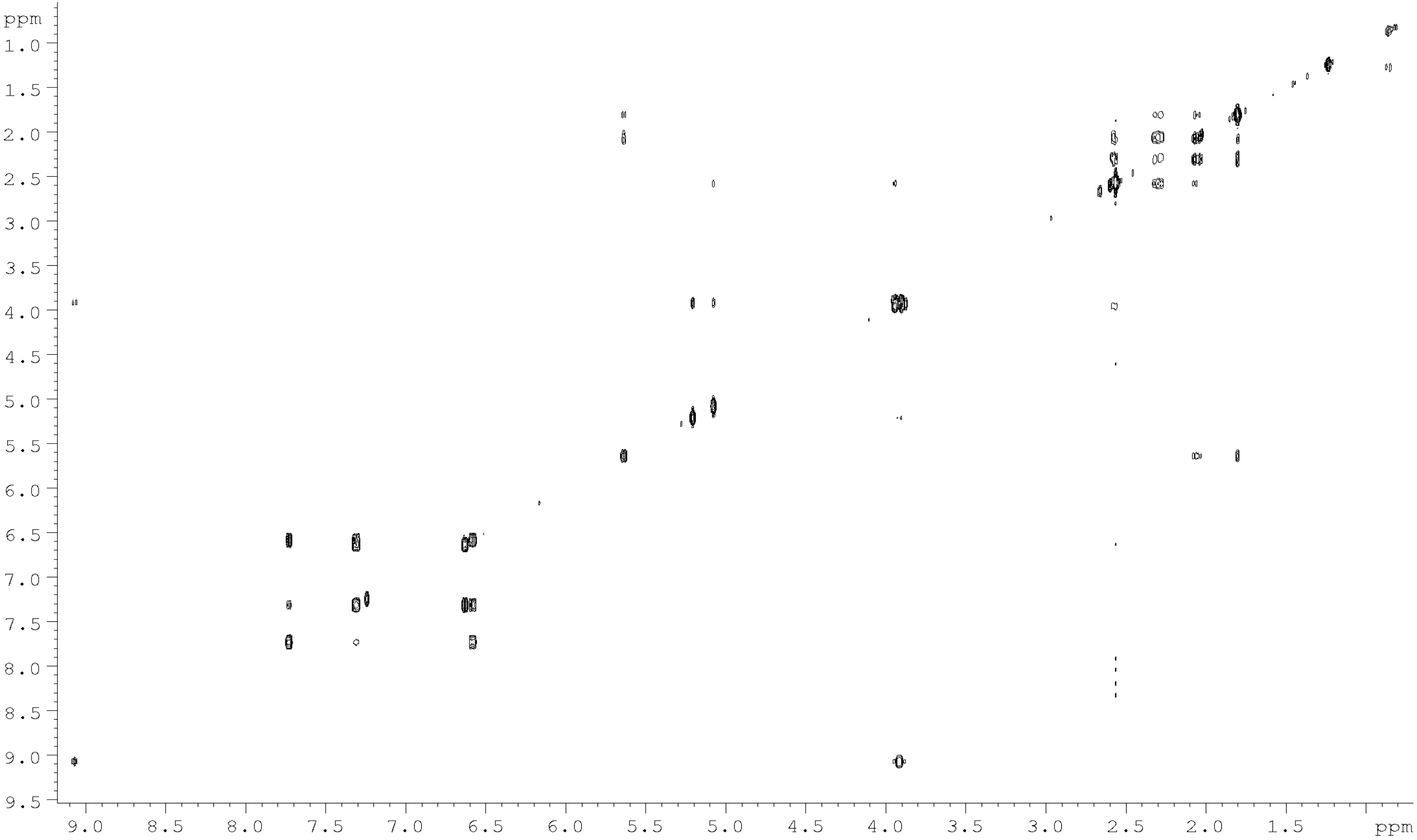

HSQC ^{13}C-^{1}H 2D heteronuclear correlation (C-H COSY) spectrum of 1-(2-(2-((1S,5R,6R)-5,6-dihydroxy-4-methylcyclohex-3-enyl)allylamino)phenyl)ethanone (**30**)

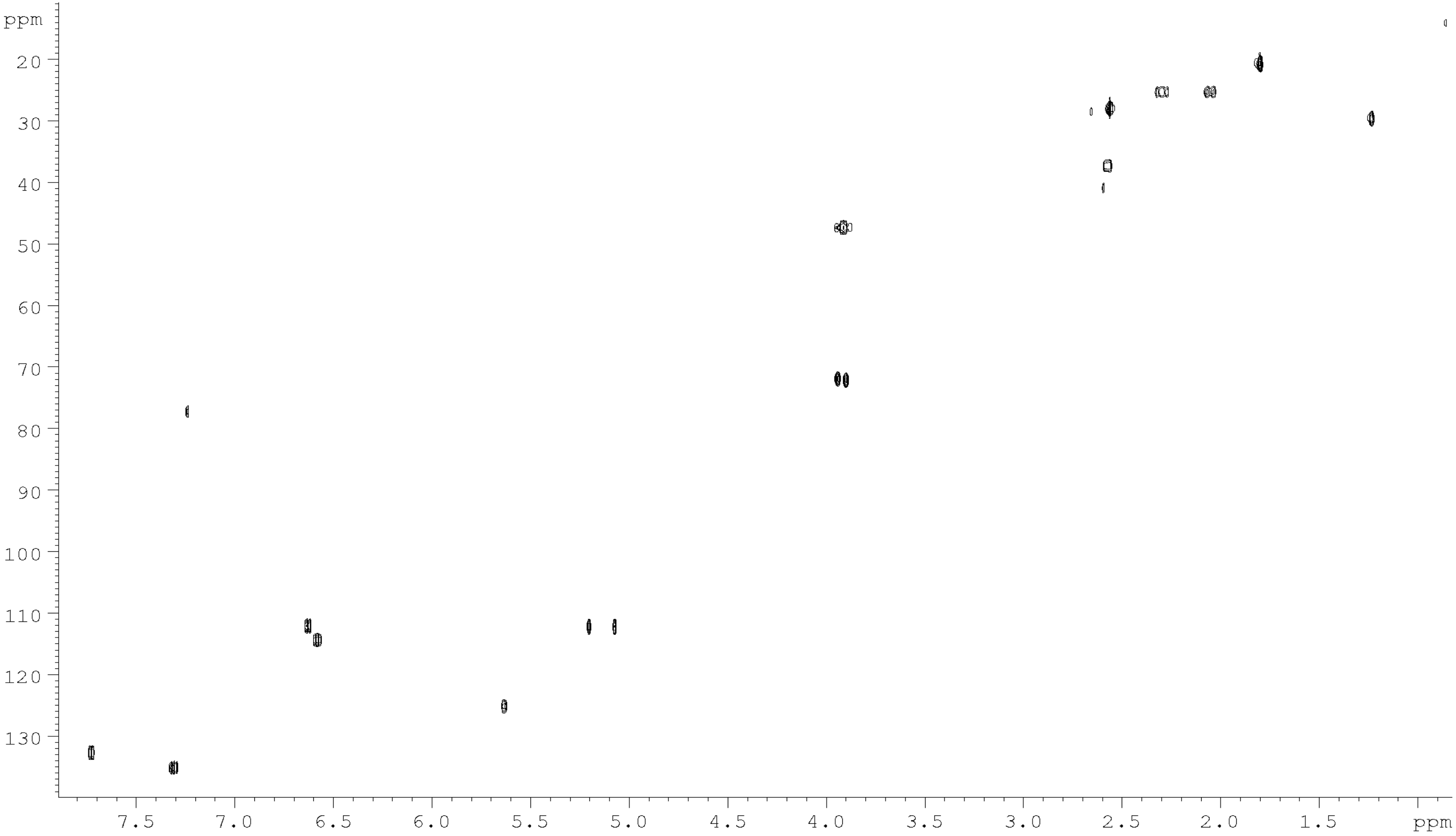

HMBC ^{13}C-^{1}H 2D heteronuclear correlation (C-H COSY) spectrum of 1-(2-(2-((1S,5R,6R)-5,6-dihydroxy-4-methylcyclohex-3-enyl)allylamino)phenyl)ethanone (**30**)

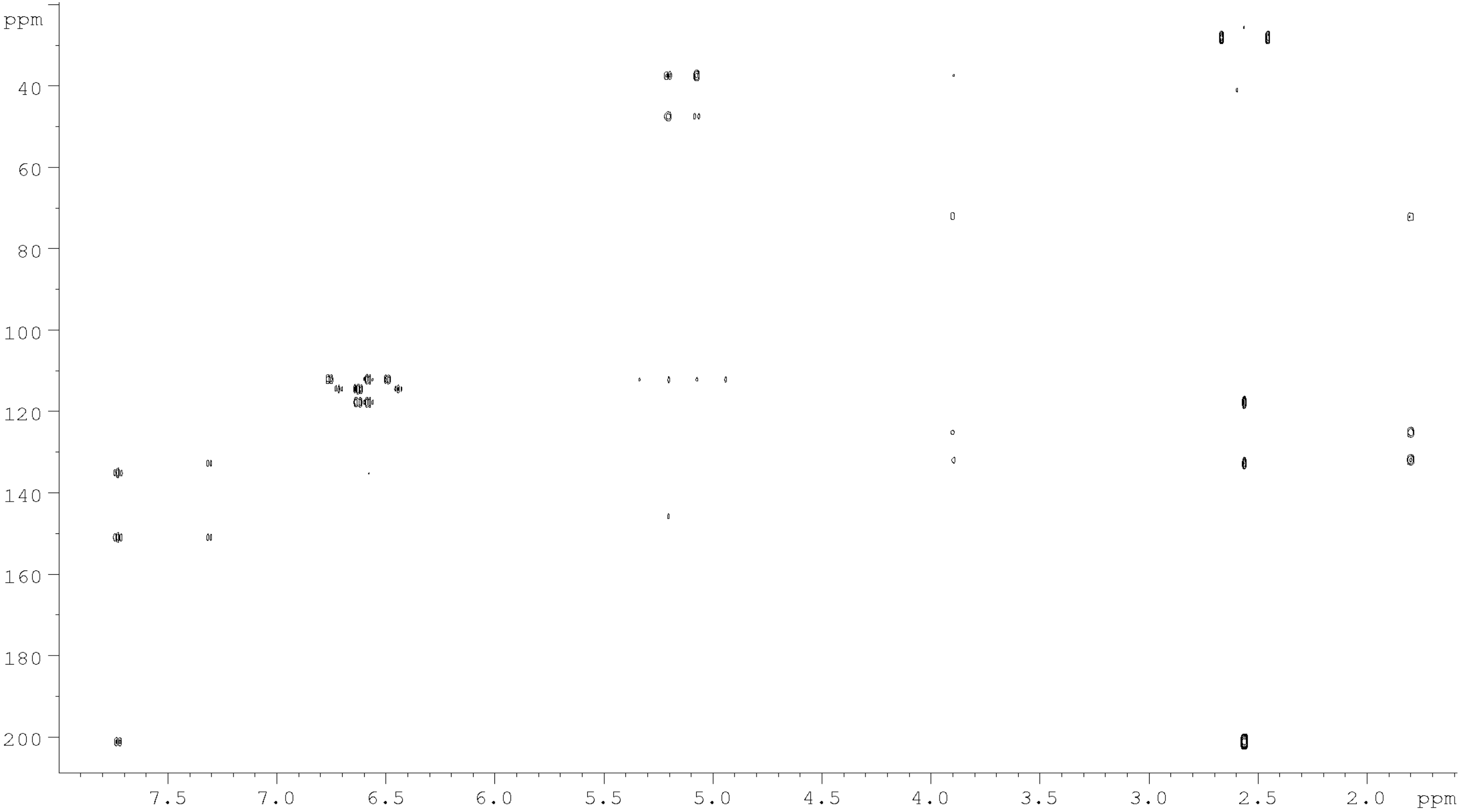

NOESY spectrum of 1-(2-(2-((1S,5R,6R)-5,6-dihydroxy-4-methylcyclohex-3-enyl)allylamino)phenyl)ethanone (**30**)

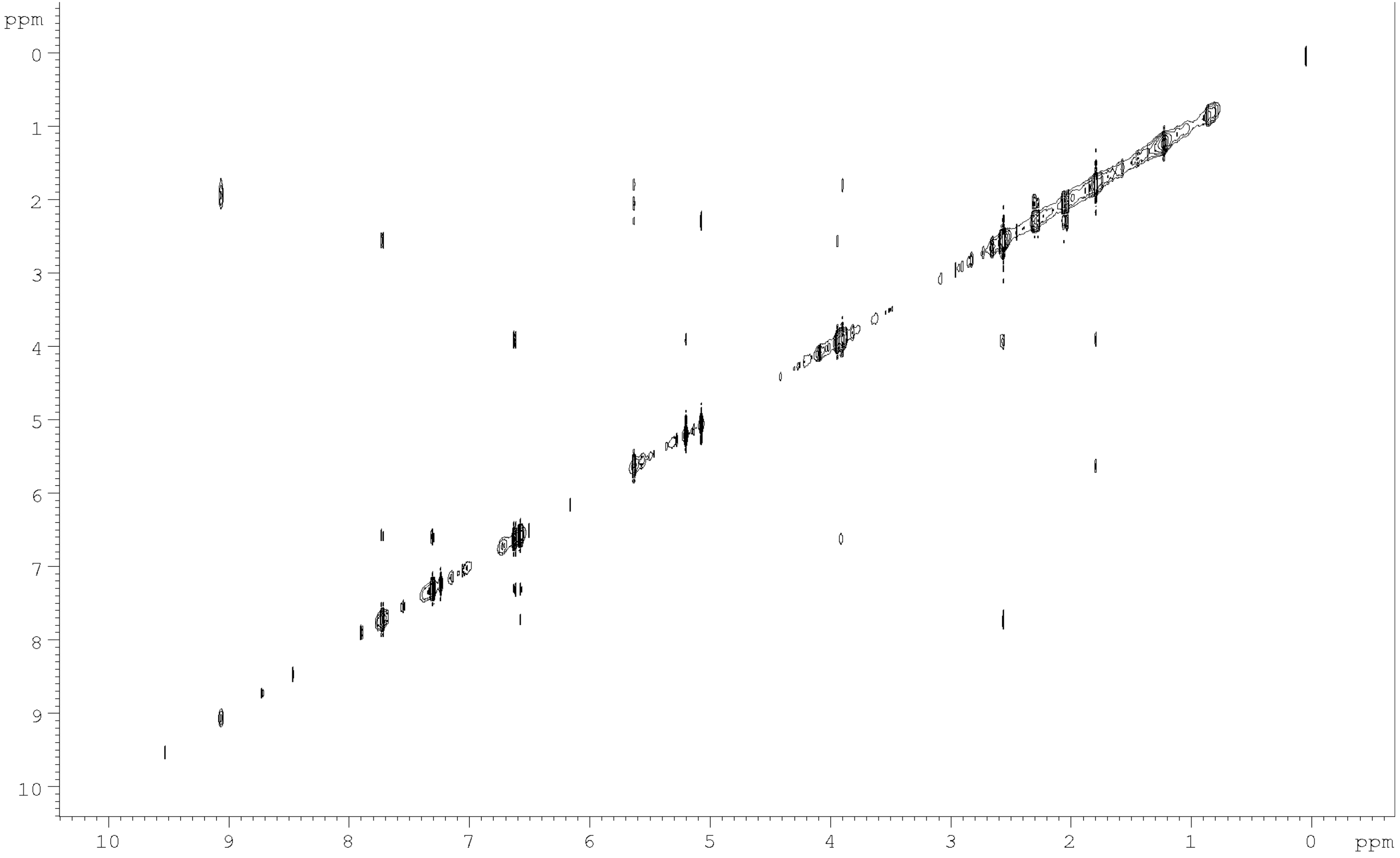

^{1}H NMR spectrum of 1-(3-(2-((1S,5R,6R)-5,6-dihydroxy-4-methylcyclohex-3-enyl)allylamino)phenyl)ethanone (**31**)

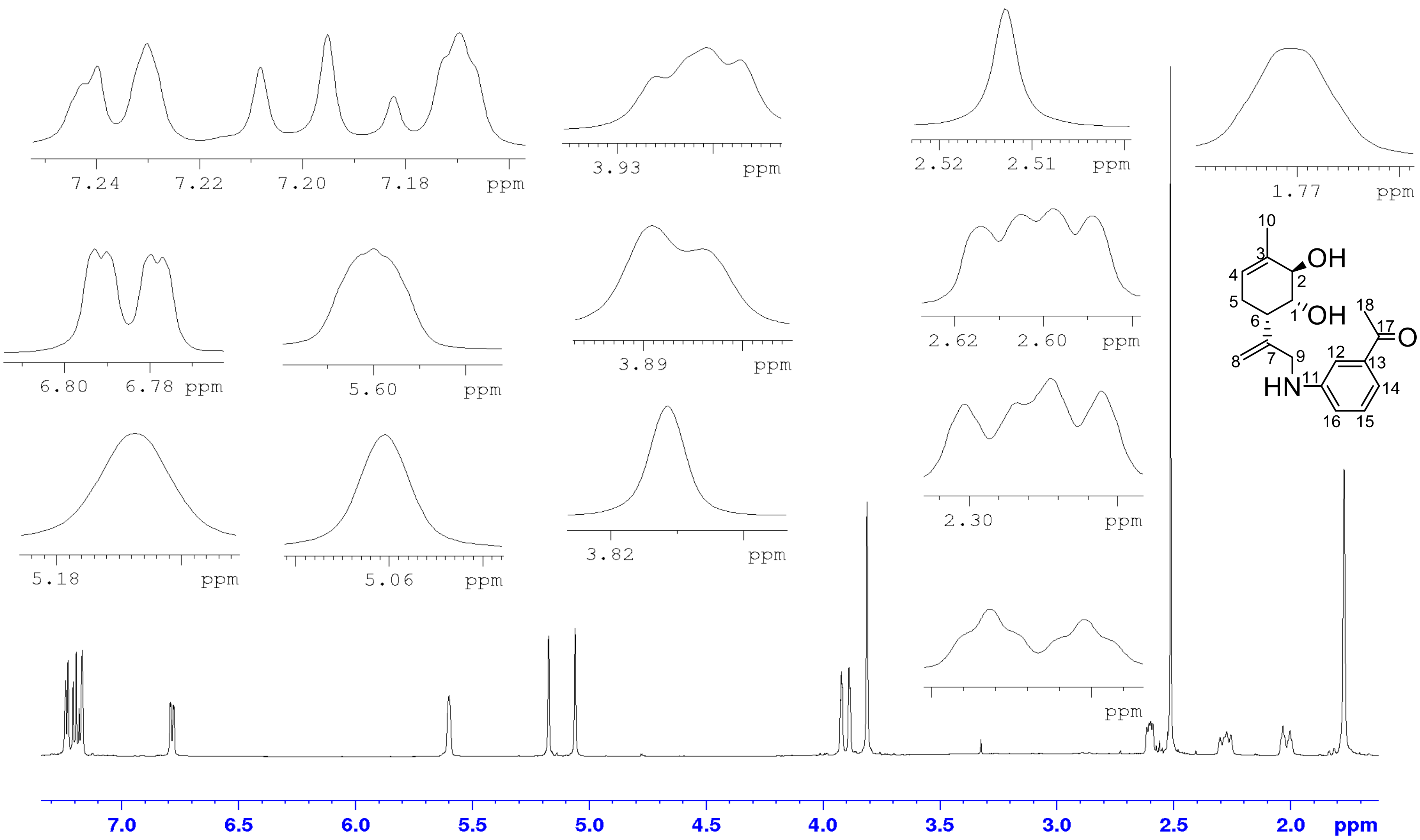

J-modulated ^{13}C NMR spectrum of 1-(3-(2-((1S,5R,6R)-5,6-dihydroxy-4-methylcyclohex-3-enyl)allylamino)phenyl)ethanone (**31**)

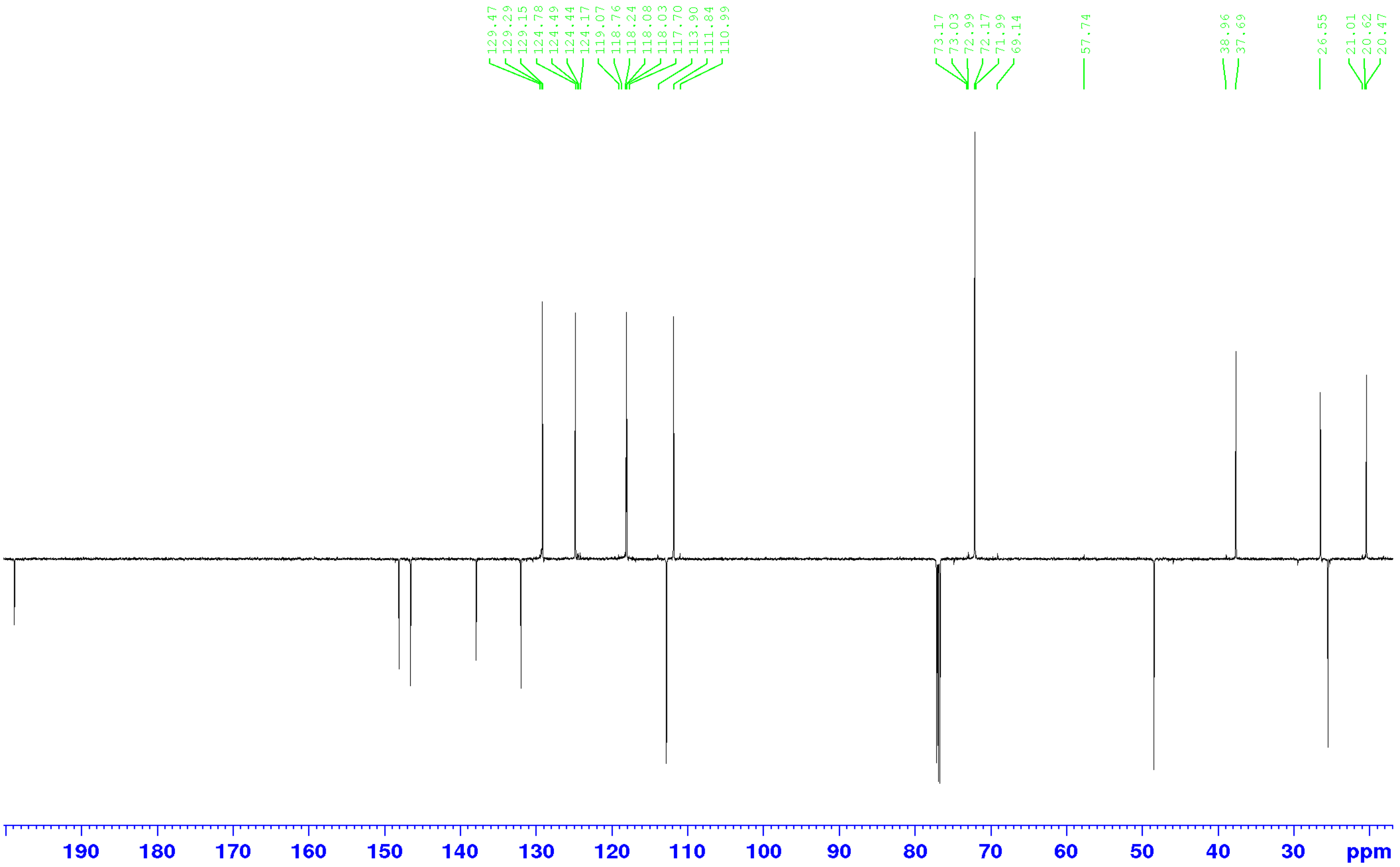

^{1}H-^{1}H 2D homonuclear correlation (COSY) spectrum of 1-(3-(2-((1S,5R,6R)-5,6-dihydroxy-4-methylcyclohex-3-enyl)allylamino)phenyl)ethanone (**31**)

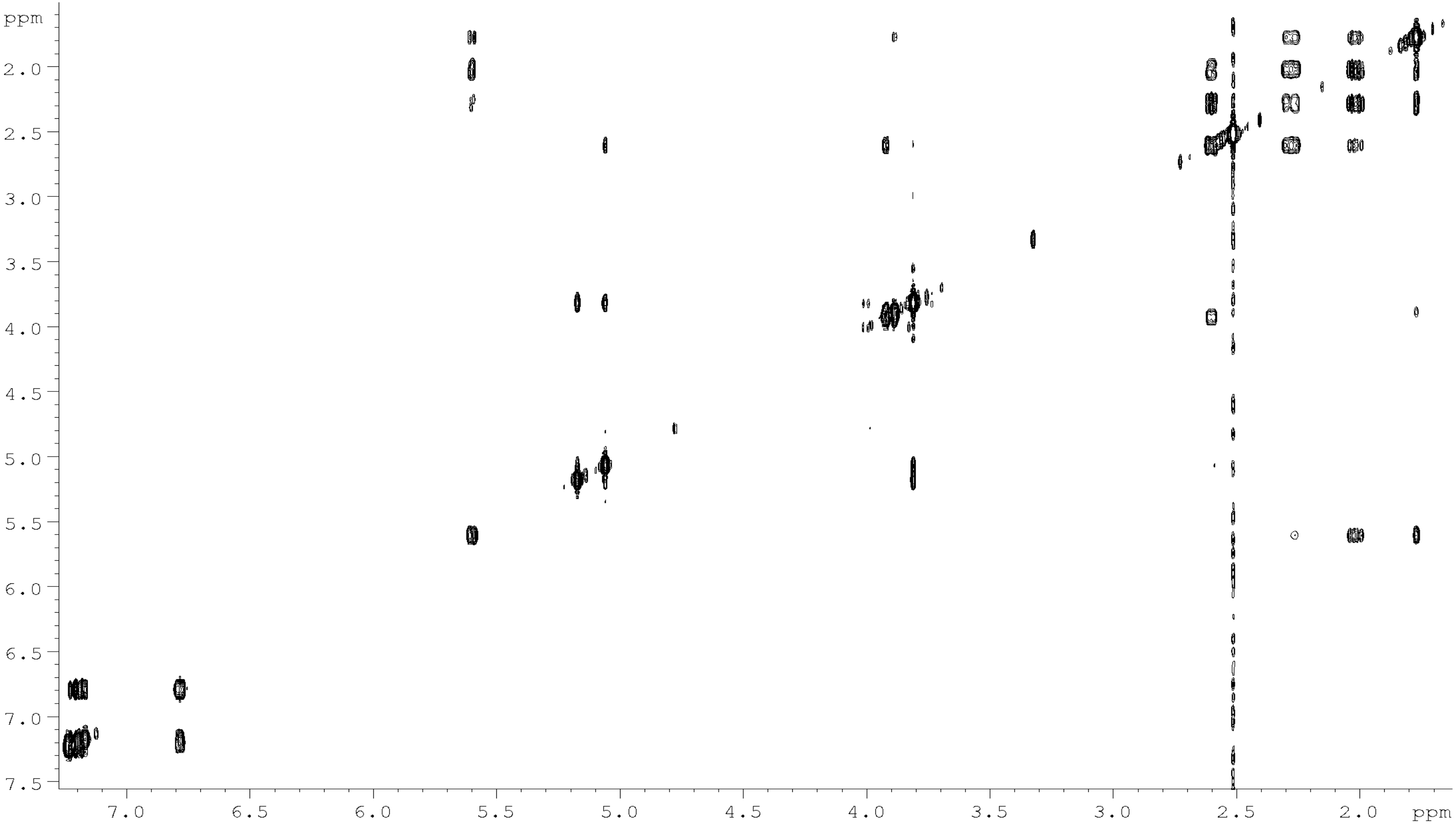

HSQC ^{13}C-^{1}H 2D heteronuclear correlation (C-H COSY) spectrum of 1-(3-(2-((1S,5R,6R)-5,6-dihydroxy-4-methylcyclohex-3-enyl)allylamino)phenyl)ethanone (**31**)

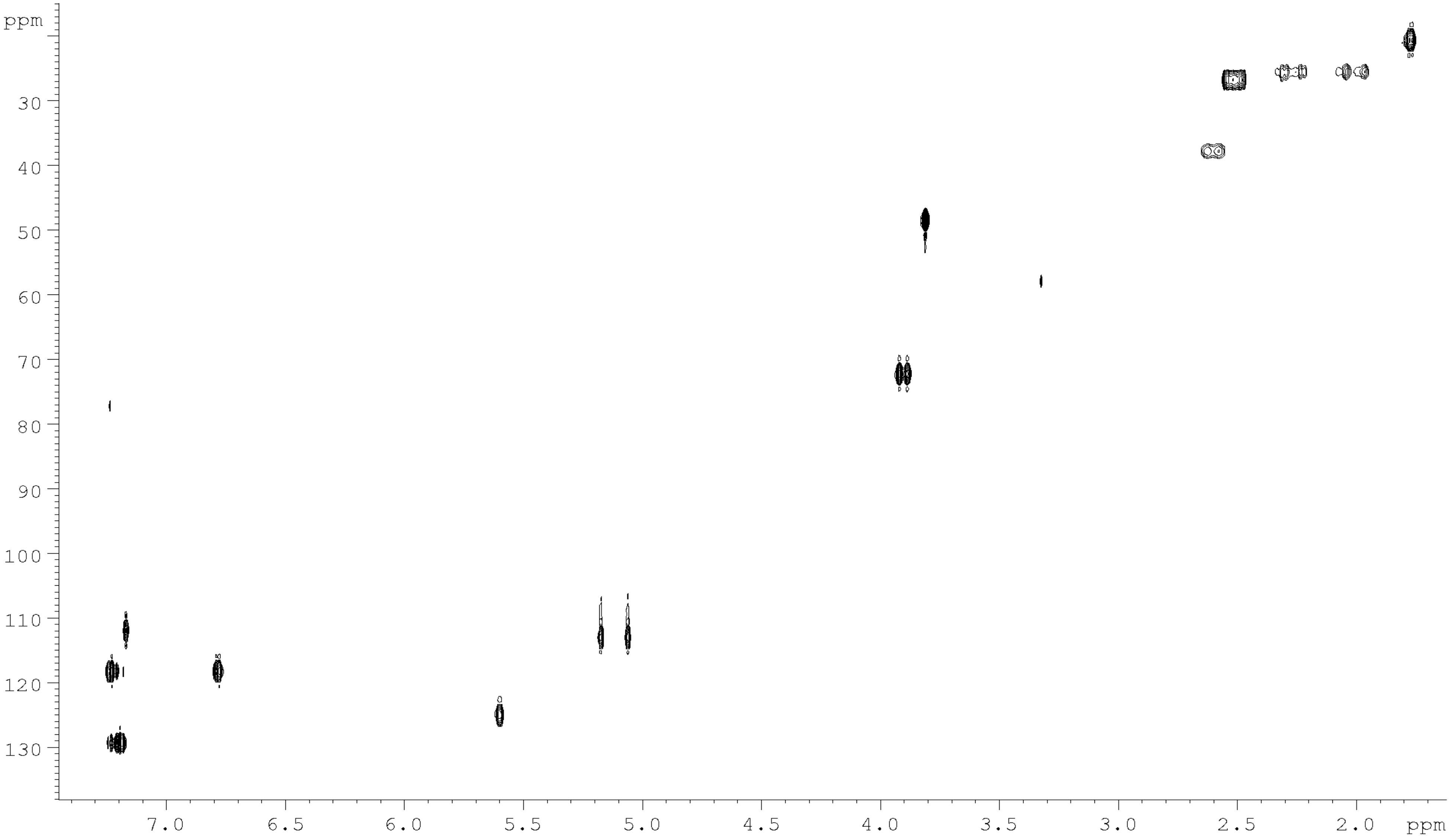

HMBC ^{13}C-^{1}H 2D heteronuclear correlation (C-H COSY) spectrum of 1-(3-(2-((1S,5R,6R)-5,6-dihydroxy-4-methylcyclohex-3-enyl)allylamino)phenyl)ethanone (**31**)

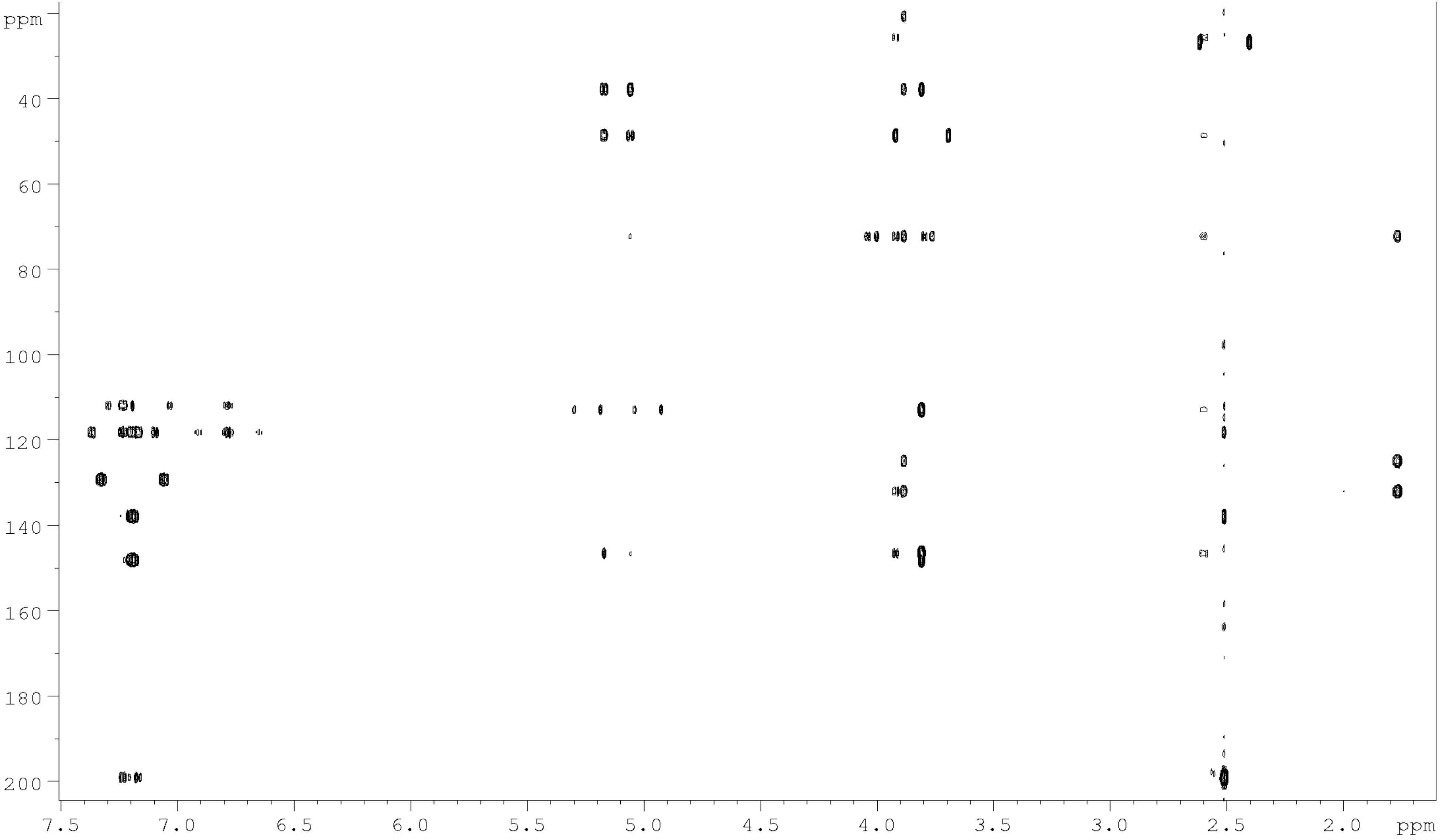

NOESY spectrum of 1-(3-(2-((1S,5R,6R)-5,6-dihydroxy-4-methylcyclohex-3-enyl)allylamino)phenyl)ethanone (**31**)

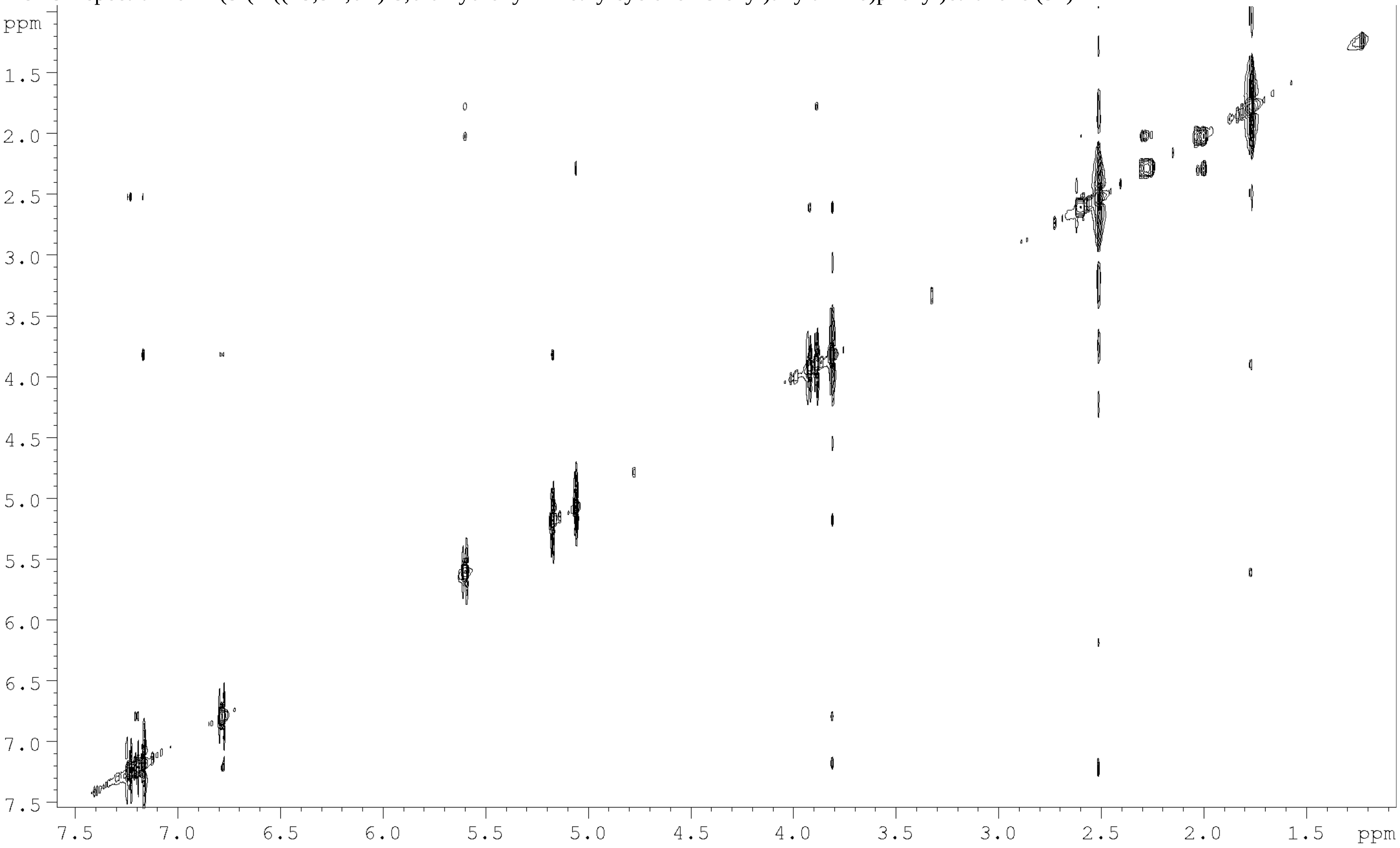

Supplement: Supplementary file 1 [file ijms-26-00097-s001.zip › ijms-3354016-supplementary.pdf]
